# Cognitive Control Challenge Task Across the Lifespan

**DOI:** 10.3389/fpsyg.2021.789816

**Published:** 2022-02-09

**Authors:** Vida Ana Politakis, Anka Slana Ozimič, Grega Repovš

**Affiliations:** ^1^Faculty of Medicine, University of Ljubljana, Ljubljana, Slovenia; ^2^Department of Psychology, Faculty of Arts, University of Ljubljana, Ljubljana, Slovenia

**Keywords:** stable cognitive control, flexible cognitive control, cognitive control challenge task, development, aging, task set switching, lifespan

## Abstract

Meeting everyday challenges and responding in a goal-directed manner requires both the ability to maintain the current task set in face of distractors—stable cognitive control, and the ability to flexibly generate or switch to a new task set when environmental requirements change—flexible cognitive control. While studies show that the development varies across individual component processes supporting cognitive control, little is known about changes in complex stable and flexible cognitive control across the lifespan. In the present study, we used the newly developed Cognitive Control Challenge Task (C3T) to examine the development of complex stable and flexible cognitive control across the lifespan and to gain insight into their interdependence. A total of 340 participants (229 women, age range 8–84 years) from two samples participated in the study, in which they were asked to complete the C3T along with a series of standard tests of individual components of cognitive control. The results showed that the development of both stable and flexible complex cognitive control follows the expected inverted U-curve. In contrast, the indeces of task set formation and task set switching cost increase linearly across the lifespan, suggesting that stable and flexible complex cognitive control are subserved by separable cognitive systems with different developmental trajectories. Correlations with standard cognitive tests indicate that complex cognitive control captured by the C3T engages a broad range of cognitive abilities, such as working memory and planning, and reflects global processing speed, jointly suggesting that the C3T is an effective test of complex cognitive control that has both research and diagnostic potential.

## 1. Introduction

Cognitive control is a general term that encompasses a variety of top-down processes that enable us to direct our thoughts and behaviour in accordance with current goals and environmental demands, and that form the basis for controlled processing of information. Key elements of cognitive control are the construction, stable maintenance, and flexible switching between relevant task sets (Dosenbach et al., 2007). Stable cognitive control, the ability to establish and robustly maintain the set of cognitive processes and information relevant to the efficient completion of an ongoing task and to protect them from interference by irrelevant stimuli and events (Lustig and Eichenbaum, 2015), is crucial for achieving set goals. Stable cognitive control, however, must be counterballanced by flexible cognitive control, that is, the ability to switch between a wide range of mental operations and adjust the selection and integration of information to what is most relevant at a given moment (Cole et al., 2013). Flexible cognitive control thus enables us to adapt to changing environmental conditions and corresponding task demands, and to prevent the perseveration of behavioural patterns that have become irrelevant or inappropriate (Dosenbach et al., 2008). The dual requirements of cognitive control—stability and flexibility—lead to the question of its foundations. Are they realised by a common or separable system, or should cognitive flexibility be regarded as a general property of the cognitive system rather than as a separable ability (Ionescu, 2012).

Studies of the neural bases of cognitive control have identified a number of distinct, functionally connected cognitive control networks (CCNs) (Cabeza and Nyberg, 2000; Duncan and Owen, 2000; Schneider and Chein, 2003; Chein and Schneider, 2005; Braver and Barch, 2006). Dosenbach et al. (2008) have linked flexible task set creation to the fronto-parietal network and stable task set maintenance to the cingulo-opercular network, suggesting that stable and flexible cognitive control are supported by different brain systems. However, the ballance between stable and flexible cognitive control has been linked to complementary effects of dopamine on the prefrontal cortex and basal ganglia (e.g., van Schouwenburg et al., 2012; Fallon et al., 2013; Cools, 2016). These results suggest that stable and flexible cognitive control, even if enabled by distinct brain systems, may be closely linked, rather than function as two independent systems.

At the behavioural level, a range of strategies and research paradigms can be used to delineate cognitive systems, from dual-task paradigms (e.g., Sala et al., 1995; Logie et al., 2004) to exploring the variance of individual differences (e.g., Engle and Kane, 2003). Most studies of cognitive control focus on its decomposition into component processes, often at the expense of the ecological validity of the instruments used. In contrast, in this paper we present and validate a novel task for assessing complex cognitive control. We use it to investigate the developmental trajectories of stable and flexible cognitive control across the lifespan and to address the question of whether stable and flexible control reflect a function of a unitary or a separable system.

### 1.1. Cognitive Control Through the Lifespan

Research across the lifespan shows that the development of cognitive abilities is subject to profound changes (Craik and Bialystok, 2006), sometimes involving the interdependence of cognitive functions. Cognitive abilities and their capacity increase during development in childhood and adolescence, peak in young adulthood, and decline with age, typically described as an inverted U-curve (Cepeda et al., 2001; Zelazo and Müller, 2002; Craik and Bialystok, 2006). Studying the development of different cognitive abilities across the lifespan can give us insights into the interdependence and possible common foundations of cognitive processes. For example, research in working memory has shown that binding and top-down control processes undergo profound changes across the lifespan (e.g., Sander et al., 2012; Brockmole and Logie, 2013; Swanson, 2017), and that declines in the capacity of visual working memory are due to both a reduced ability to form independent representations and a reduced ability to actively maintain those representations in the absence of external stimuli (Slana Ozimič and Repovš, 2020).

Because of the complexity of cognitive control, studies of cognitive control across the lifespan have focused primarily on its constituent cognitive processes and abilities, such as processing speed (Kail and Salthouse, 1994), inhibitory control (e.g., Williams et al., 1999; Christ et al., 2001), interference control (Gajewski et al., 2020), task coordination (Krampe et al., 2011), and working memory (e.g., Blair et al., 2011; Sander et al., 2012; Alloway and Alloway, 2013; Brockmole and Logie, 2013). All component processes show the expected inverted U development curve—they improve into adolescence (Anderson et al., 2001) and decline with age (e.g., Cepeda et al., 2001; Zelazo et al., 2004)—however, specific developmental timelines differ from component to component (Diamond, 2013).

Although many studies have examined changes in specific components of cognitive control across the lifespan, to our knowledge there are no studies that examine the development of complex cognitive control or that focus on the comparison between stable maintenance and flexible switching between complex task sets. Some information can be derived from studies of working memory and task switching, respectively. Stable cognitive control is most closely associated with working memory, which has been proposed as the fundamental process that enables cognitive control and “prevents the tyranny of external stimuli” (p. 354 Goldman-Rakic, 1994). Flexible cognitive control, on the other hand, is closely related to task-switching paradigms in which participants have to rapidly switch between two simple task rules. It is most directly indexed by the local switch cost, defined as the difference in performance on switch and repeat trials within mixed blocks, rather than the global switch cost, defined as the difference in performance between pure and mixed blocks, as the latter also reflect the additional load on working memory when multiple task sets must be kept online (Wasylyshyn et al., 2011). Studies of working memory (e.g., Cabbage et al., 2017) should therefore provide some information about the development of stable cognitive control, and studies of task switching (e.g., Wasylyshyn et al., 2011; Holt and Deák, 2015) should inform us about the development of flexible cognitive control. However, each of these studies in isolation cannot fully capture the complex nature of flexible and stable cognitive control, nor inform us about their interdependence.

### 1.2. Measuring Cognitive Control

Cognitive control is a construct that is difficult to measure because by definition, its effects can only be observed indirectly, e.g., through its influence on perception, integration of information, resolution of stimulus-response conflicts, task switching, planning, etc. Many standard tests of cognitive control therefore tap into a range of processes that are outside their primary purpose, which can lead to increased measurement error due to task contamination when measuring cognitive control (Burgess, 1997; Burgess and Stuss, 2017).

Furthermore, due to the complexity of cognitive control, standard tests of cognitive control have focused primarily on measuring single constituent abilities or processes of cognitive control (e.g., task switching, inhibition, verbal fluency, planning). Classic tests of cognitive control, such as the WCST (Berg, 1948) or the Stroop colour-word test (Stroop, 1935), have provided many important insights into changes across the lifespan in performance monitoring and stimulus-response conflict resolution, respectively (Braver and Ruge, 2001; Chan et al., 2008). Constitutive cognitive control functions can be precisely operationalised and objectively quantified, but they can individually measure only a small facet of cognitive control (Burgess, 1997) and do not provide a complete understanding of the development of cognitive control.

To address these problems, there have been calls for the development of tasks with better ecological validity that measure cognitive control in complex, unstructured situations where the rules of the task are not clear (Burgess and Stuss, 2017). Despite some progress in developing more naturalistic tests (e.g., Schwartz et al., 2002; Schmitter-Edgecombe et al., 2012), both researchers and practicing neuropsychologists continue to require new methods for measuring cognitive control.

### 1.3. The Cognitive Control Challenge Task

To contribute to the assessment of complex cognitive control, we developed the Cognitive Control Challenge Task (C3T). Unlike most other standardised tests of executive function, which are highly structured and constrained by specific task rules, participants in the C3T receive only general instructions on how to perform the task. The formation and implementation of specific strategies—an important function of complex cognitive control (Botvinick et al., 2001)—is left to the participants themselves.

The C3T was explicitly designed to assess the ability to create, maintain, and flexibly switch between complex task sets that support the processing and integration of information from multiple modalities and domains and that require the engagement and coordination of multiple cognitive processes and systems (e.g., selective attention, working memory, deduction, behavioural inhibition, decision making).

In C3T, participants complete several trials consisting of two parts. In the second part, the response part, two visual stimuli, a picture and a written word, and two auditory stimuli, a sound and a spoken word, are presented simultaneously. The visual stimuli are each presented on one side of the screen, while the auditory stimuli are presented separately to each ear. The participant is asked to evaluate the stimuli using complex rules (e.g., indicate, which of the stimuli represents a smaller animal; see also **Table 2**) and answer by pressing the left or right button as quickly as possible. The rule to be applied is presented in the first, preparatory part of the trial. The participant is instructed to process the rule and proceed to the response part only once they understand the rule and are ready to apply it. In this way, the trial structure of C3T allows for separate estimates of the time required to set up a task set (preparation time), the time required to apply it (response time), and the accuracy of the response.

C3T is performed in two modes, each consisting of blocks of trials. First, in the stable task mode, each of the rules is used throughout a block. This allows observing the time required to construct a new task set when a participant first encounters a rule, as well as the time required to refresh the task set on subsequent trials. Next, in flexible task mode, the rules change from trial to trial. Since the rules are well-learned in advance, the time required to switch between several previously encoded complex task sets can be observed. The separation and fixed order of the task modes also allows different types of training to be observed. Improvement over trials in the stable task mode provides information on task set acquisition, optimisation, and progress in its execution, while the flexible task mode provides specific information on improvement in task set switching.

To the best of our knowledge, C3T is the first task that specifically examines complex stable and flexible cognitive control. It measures (i) the formation of complex task sets, (ii) their maintenance, and (iii) flexible switching between them. Compared to simple switching tasks, it requires the formation and switching between complex task sets that require the integration of multiple aspects and modalities of task stimuli and involve multiple cognitive systems. This also distinguishes it, in part, from other more complex cognitive control tasks such as the Wisconsin Card Sorting Task (WCST; Somsen, 2007), and the Dimensional Change Card Sort (DCCS; Zelazo, 2006). While both WCST and DCCS focus primarily on reasoning, rule discovery, and perseveration, C3T provides a measure of efficiency in encoding, maintaining, and switching between complex task sets. C3T is suitable for use from the time children acquire reading and basic numerical skills (counting and number comparison) through late adulthood, and can therefore provide insights into the development of stable and flexible cognitive control and their potential interdependence across the lifespan.

### 1.4. The Aims of the Study

The main aim of the study is to evaluate the performance on C3T across the lifespan in order to (i) assess the properties of the newly developed C3T, (ii) investigate changes in different modes of complex cognitive control across the lifespan, and (iii) investigate the extent to which stable and flexible control reflect the functioning of separable and shared systems, respectively, by observing the extent to which the two aspects of cognitive control follow the same or different developmental curves.

We expect C3T to distinguish between time to set up new task sets, performance during stable use of task sets (stable task mode), and performance during task set switching (flexible task mode), providing estimates of task set encoding, stable and flexible cognitive control.

Next, we expect that C3T will prove sensitive to lifespan changes in cognitive control processes. Given previous findings on the development of cognitive abilities in general and cognitive control specifically, we expect an inverted U-shaped relationship of C3T performance with age.

Finally, we expect that the development of stable and flexible cognitive control differs across the lifespan. In particular, based on previous studies showing rigidity and perseverations on tests of cognitive control (Head et al., 2009) along with a reduced ability to maintain and coordinate two task sets in working memory (Wasylyshyn et al., 2011), we expect that in aging, the ability to switch between task sets, as reflected in preparation time in the flexible task mode, will decline more rapidly than the ability to maintain task sets in the stable task mode. This should lead to an increase in the estimated switching cost, indexing the difference between preparation time in stable and flexible task mode.

In contrast, studies in children suggest a reverse pattern. Whereas even 4-year-olds are already able to switch between abstract rules (e.g., Diamond, 1996; Bub et al., 2006), children are often unable to maintain appropriate task set (Deák et al., 2004; Carroll et al., 2016). Working memory (Huizinga et al., 2006) and the ability to suppress task-irrelevant information (Anderson et al., 2001) develop more slowly compared to cognitive switching and inhibition. Thus, we expect children to have relatively more difficulty with task maintenance than with task switching compared to young adults. This should translate into smaller differences between preparation times in the flexible compared to the stable task mode, and thus lower switching cost.

In summary, we predict that due to the earlier maturation of flexible compared to stable cognitive control, the switching cost index should increase throughout the observed lifespan, even if individual lifespan development for stable and flexible control follows a U-shaped curve. This result would support the hypothesis that stable and flexible cognitive control depend on separable systems with different specific developmental trajectories.

## 2. Method

### 2.1. Participants

One hundred and ninety-three participants were recruited in the initial sample (IS), of whom 37 were excluded, 5 because of head injury, 2 because Slovene was not their first language, 4 because of missing data and/or failure to complete the task, and 26 because of low task accuracy (less than 57.5%)^1^. Results from the 156 remaining participants (103 females, mean age 30.2 years, range 10–83 years) were analysed. 247 participants were recruited for the replication sample (RS), of whom 63 were excluded, 9 due to head injury, 12 because Slovene was not their first language, 28 due to missing data and/or failure to complete the task, 14 due to low accuracy (less than 57.5%). The results of the 184 remaining participants (126 females, mean 32.6 years, range = 8–84 years) were included in the analysis. (See Table 1) for the composition of the samples, and Supplementary Table 1 for age distribution of participant excluded due to low task accuracy.

**Table 1:** Participants.

		**Initial sample**		**Replication sample**		**Together**	
**Developmental stage**	**Age (years)**	***N***	**F (%)**	***N***	**F (%)**	***N***	**F (%)**
Late childhood	8–12	4	2 (50)	14	6 (43)	18	8 (44)
Adolescence	13–17	27	15 (56)	26	11 (42)	53	26 (49)
Emerging adulthood	18–30	79	54 (68)	81	61 (75)	160	115 (72)
Young adulthood	31–45	9	7 (78)	10	7 (70)	19	14 (74)
Middle adulthood	46–64	19	11 (58)	25	16 (64)	44	27 (61)
Late adulthood	65–84	18	14 (78)	28	25 (89)	46	39 (85)

Data collection was carried out as part of a Cognitive Psychology laboratory course in two phases. First, all students completed the C3T and standard cognitive tests themselves. Next, each student was asked to recruit and test four neurotypical participants from four different age groups. In this way, the data collection protocol was designed to recruit a heterogeneous sample of participants of different ages. Besides completing the tests themselves, students received detailed written instructions and hands-on training on the use of the instruments, the study protocol, and the importance and practice of obtaining informed consent. We emphasised that even if potential participants were interested in performing the task, they were in no way required to sign an informed consent form and that their data would not be used in this case. The final sample size reflects the number of participants who met the exclusion criteria, where all relevant data were properly collected and participants gave their signed informed consent.

To address the possibility of confounding neuropsychological disorders in the older adults, we have checked the cognitive status of participants in the Late Adulthood group by assessing their profile across the cognitive tests and self reported measures of cognitive and memory failures. No outlier was identified that would merit exclusion (see Supplementary Material).

The study was approved by the Ethics Committee of the Faculty of Arts, Ljubljana, Slovenia.

### 2.2. Materials and Procedures

Each participant performed the C3T task and a series of standard psychological–cognitive tests in one or two sessions after signing an informed consent form. Students completed the testing during their laboratory course and were asked to bring their PCs to participate in the study. Additional participants were tested outside the laboratory, usually in their home environment.

#### 2.2.1. Cognitive Control Challenge Task (C3T)

**Figure 1:**
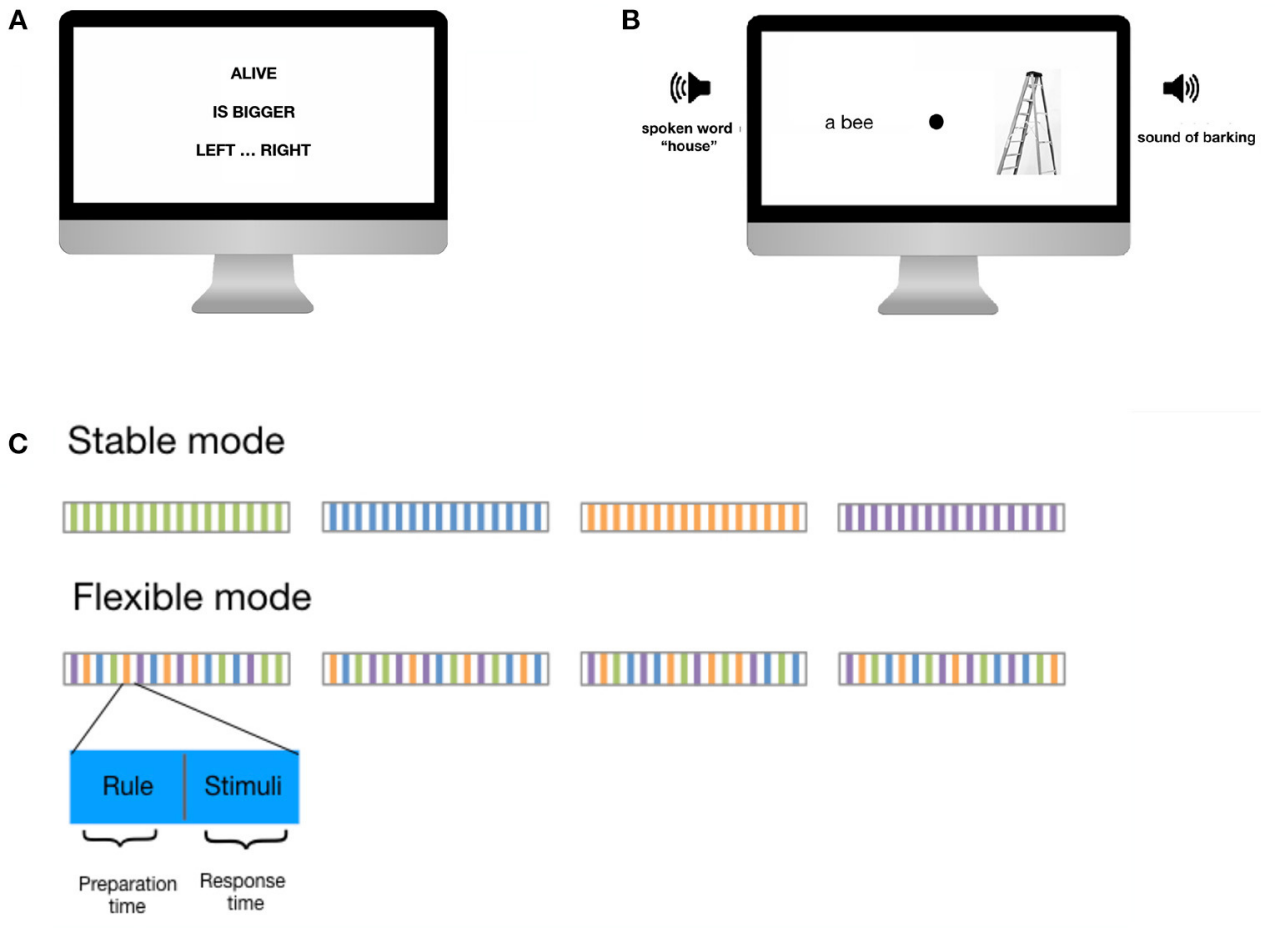
Example trial of the C3T. **(A)** First a three-element rule is presented. **(B)** After pressing a button, a set of visual and auditory stimuli is presented to which the participant must respond. In the example shown, the participants had to focus on those stimuli relating to living creatures (in this case a bee and a dog), compare which of the two is larger, and press the left button if the larger animal was presented on the left, or the right button if the larger animal was presented on the right. Since a dog is bigger than a bee and the barking of the dog was presented on the right side, the correct answer was to press the right button. **(C)** Time course of the task in stable and flexible mode. Preparation time is the time from the presentation of the rule until the button is pressed to have the stimuli presented. Response time is the time from the presentation of the stimuli to the response.

The C3T asks participants to evaluate and respond to a series of simultaneously presented visual and auditory stimuli based on previously presented complex task rules. The rules are either stable over a block of trials (stable task mode) or change pseudo-randomly from trial to trial (flexible task mode). More specifically, each trial of the task follows the same structure (Figure 1). Initially, one of four different task rules is displayed on the screen. Each rule consists of three elements: Information about which stimuli to focus on, information about how to evaluate the stimuli, and instructions about how to provide the answer. Participants are asked to fully encode the rules and press a button when they are ready to have the stimuli presented. At this point, four stimuli are presented simultaneously: two visual stimuli, one on each side of the screen (a written word and an picture) and two auditory stimuli, one to each ear (a sound and a spoken word). Participants are asked to provide their answers as quickly as possible by pressing the left or right key on a keyboard, based on the provided rule. The next trial begins after a fixed inter-trial interval of 2 s.

The progression from the rule to the presentation of the stimuli is self-paced, so that (i) the preparation time required to activate a relevant task set and (ii) the response time and accuracy in performing a task set are recorded separately. These times can then be examined in three contexts. First, the case in which participants are confronted with a particular rule for the first time. The times in this case reflect the initial creation of complex task sets that require the integration (or, if necessary, inhibition) of multiple cognitive modalities and domains. We call this the setup time. Second, the stable mode trials, where participants only need to maintain or possibly update and reactivate the current task set. Third, the flexible mode trials, where participants must switch between task sets by inhibiting the task set that was relevant to the previous trial and reestablishing the task set that is relevant to the current trial.

In the stable task mode, participants completed 12 (16 in RS) consecutive trials of each of the four rules. In the flexible task mode, participants again completed 12 (16 in RS) trials with each of the four rules, but the specific rule to be followed changed pseudo-randomly from trial to trial. In the replication sample, two of the more difficult rules were replaced with two slightly simpler rules (Table 2).

**Table 2:** Rules of the C3T in the initial and the replication sample (^*^ grammatical gender).

**Rule**				**Sample**	
**No**.	**Focus on**	**Evaluate**	**Response**	**Initial**	**Replication**
1	Left	Sum	Even | odd	x	.
2	Word	Noun	Left | right	x	.
3	Image	Fits together	Yes | no	x	x
4	Alive	Smaller	Left | right	x	x
5	Right	Same valence	Yes | no	.	x
6	Visual	Female^*^	Left | right	.	x

The flexible task mode always followed the stable task mode, using the same rules but different stimuli. The fixed order of stable and flexible task modes allowed us to separately observe first the dynamics of task set acquisition in stable mode and then, once the rules were well-learned, the cost of switching and the dynamics of optimising the switching of task sets over the course of the task, without the confound of concurrent task set learning.

**Figure 2:**
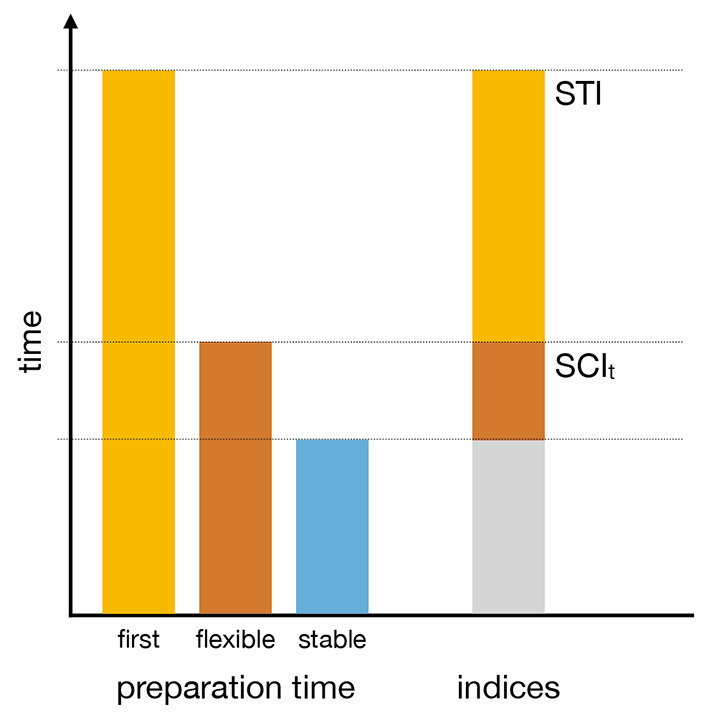
Schematic representation of the derived time performance indices. The left side of the figure illustrates the estimates of preparation times when faced with a rule for the first time (*first*), during performance in flexible task mode (*flexible*), and during performance in stable task mode (*stable*). The right side of the figure shows that the Switching Cost Index (*SCI*_*t*_) reflects the additional time required to switch between complex task rules compared to stable task rule maintenance. The Setup Time Index (*STI*) reflects the additional time required to set up complex task rules when they are encountered for the first time.

This task design allowed us to compute three derived performance indices that serve as direct measures of the specific processes of interest. Two indices are based on preparation times (see Figure 2). The Setup Time Index (*STI*) reflects the additional preparation time required to set up a complex task set compared to the preparation time required to switch between known complex task sets. The time-based switching cost index (*SCI*_*t*_) reflects the additional preparation time required to switch between known task sets compared to maintaining or refreshing an already active task set. The third index is an error-based switching cost index (*SCI*_*e*_) that reflects the additional performance difficulty of switching between task sets compared to using the already active task set.

In both samples, participants went through a short practice before performing the core task. During practice, the principle of solving the task was explained on two separate rules that were then not used in the actual task. Depending on the participant's pace, the exercise and task performance took between 20 and 30 min.

The task was performed on a personal computer. The experimental task, stimulus presentation, and recording of behavioural responses were implemented in PsychoPy2 version 1.78.01 (Peirce et al., 2019). The task was designed to run on a variety of computers with different screen sizes and resolutions. Visual stimuli were presented in the center 800 × 600*px* (IS) and 1000 × 600*px* (RS) of the screen on a white background. The center of the screen was indicated by a dark grey circle with a radius of 10*px*. Task stimuli were presented in the center of the left and right halves of the task display, 200*px* (IS) and 300*px* (RS) to the left and right of the central fixation point, respectively. For IS, the images were selected to fit within a square of 250 × 250*px*; for RS, they were scaled to a uniform size of 400 × 400*px*. Auditory stimuli were processed so that they did not exceed 1*s* in duration and were prepared as 44.1*kHz* stereo waveform files, with the signal present only in the relevant channel (left or right). To ensure spatial separation of the auditory stimuli, they were presented with headphones.

Both the visual and auditory stimuli were selected to represent clearly identifiable inanimate objects (e.g., a car, a house, a piano, a number of squares or circles), animals (e.g., a horse, a cat, a tiger, a snake), people (e.g., a person crying, a baby smiling), or events (e.g., clapping, a siren, a person singing). The visual and auditory material for the task was obtained from freely available Internet databases with appropriate licences to use the material (FreeImages.com, Creative Commons Attribution, CC0 Public Domain, Commons) or created by the authors of the task. A list of attributions can be found in the Supplementary Material (Supplementary Tables 23, 24).

#### 2.2.2. Testing Protocol

Participants completed a series of cognitive tests that focused primarily on cognitive control and fluid intelligence. Specifically, they first completed a set of paper-pencil tests: A digit and letter span test of working memory that included forward and backward digit span, alphabetic letter span, and even-odd position digit span; a verbal fluency test with lexical, semantic, and category switching tasks; and a publicly available version of the Trail making (TM) test (TM; Reitan and Wolfson, 1985) with an additional sensorimotor control condition (*TM*_*C*_). Next, they performed a set of computerised test: the C3T task and either a computerised version of the Tower of London test (TOL; Shallice, 1982) (IS only) or an automated computerised version of operational span (ospan) based on the original test by Unsworth et al. (2005) (RS only). When administering the computer-based tests, participants were asked to sit comfortably in front of the computer so that the screen was clearly visible and they could easily give the required responses. For details on the tests used, see “Standard Tests of Cognitive Control” in the Supplementary Material. The tests were always performed in the same order, however, they did not have to be completed in the same sitting, if the participant felt tired. If the testing was split into two sessions, they were completed either within the same day or within a span of a few days. Lastly, the participants also completed a computerised version of the Cognitive Failures Questionnaire (CFQ; Broadbent et al., 1982) and Prospective and Retrospective Memory Questionnaire (PRMQ; Smith et al., 2000).

Though detailed comparison of the C3T with other tests of cognitive control was outside of the scope of this study, we included them as a coarse external validity test. To limit the burden on the participants we selected the tests that were short to administer and indexed aspects of stable and flexible cognitive control. Working memory tests were selected to provide estimates of the ability for stable maintenance of information. Of these span tasks were included to measure the ability for active maintenance of verbal information, whereas operational span was included as a measure of working memory that loads more on the executive control and correlates with fluid intelligence. Trail making test and verbal fluency tests were included as measures of general speed of processing and cognitive flexibility. Tower of London was included as a test of complex cognition and planning. We were specifically interested in correlation with TOL reaction times as they should reflect speed of processing when confronted with task that require complex integration and manipulation of information. We have not included WCST and simple tasks switching test due to their length and difference in focus, as described in the introduction.

CFQ and PRMQ were not included in the analysis but were used as an indicator of subjective cognitive complaints by older adults (see Supplementary Material for details).

### 2.3. Analysis

#### 2.3.1. Reaction Times

The initial analyses of reaction times required estimates of the average time for each participant, for each task mode, and for each trial number separately. Because there were only four trials with the same trial number in each task mode, to minimise the effects of outliers, we computed the median as the average time of trials with a correct response and used it for these analyses.

In further analyses of reaction times, the averages across all trials were used, which enabled computation of more robust reaction time estimates. For these analyses we identified and excluded outlier reaction times separately for each participant and each task mode. First, we excluded all trials with response times shorter than 200 ms or the preparation or response times longer than 60 s. Next, we calculated the interquartile range (*IQR*) and excluded all reaction times that fell outside 1.5 × *IQR* from the second or fourth quartile. On average, we excluded between 10–13% of trials using this procedure. Because the procedure for removing outliers can potentially affect the results, we repeated all analyses by excluding all trials in which reaction times deviated more than 2.5 SD from the mean, and by using the median instead of the mean to compute the average reaction time. In all cases, the analyses yielded the same pattern of results.

#### 2.3.2. Derived Measures

The three performance indices, *STI*, *SCI*_*t*_, and *SCI*_*e*_ were computed using the following equations:


(1) $$\begin{array}{r} STI={\bar{t}}_{i}-{\bar{t}}_{f} \end{array}$$

(2) $$\begin{array}{r} SCI_{t}={\bar{t}}_{f}-{\bar{t}}_{s} \end{array}$$

(3) $$\begin{array}{r} SCI_{e}=e_{f}-e_{s} \end{array}$$

where ${\bar{t}}_{i}$ is the median preparation time on trials where the participant is confronted with a new rule for the first time, ${\bar{t}}_{f}$ and ē_*f*_ are the mean preparation time and error rate, respectively, for flexible trials, and ${\bar{t}}_{s}$ and ē_*s*_ are the mean preparation time and error rate, respectively, for stable trials. The preparation times at first presentation of each rule are excluded from the computation of ${\bar{t}}_{f}$ and ${\bar{t}}_{s}$.

#### 2.3.3. Statistical Analyses

##### 2.3.3.1. Regression Analyses

To investigate the effects of factors in models that included continuous predictor variables, such as trial order and the age of participants, we used regression analyses. In the analysis of effects on response accuracy, we used binomial logistic regression. When models included within-subject repeated measures predictors (trial order and task mode), participants were modelled as a random effect. The estimates of statistical significance and effect size for individual predictors and their interactions were obtained by comparing full model with the model without the effect of interest (a reduced model) and testing for a significant difference using a χ^2^ test. The *R*^2^ statistics for the model comparison were calculated using the function *r.squaredGLMM* from the *MuMIn* library (Barton, 2017), which enabled computation of Nakagawa and Schielzeth's *R*^2^ for mixed models (Nakagawa and Schielzeth, 2013).

##### 2.3.3.2. Robust Regression Analyses

To reduce the effects of reaction time outliers, especially in relatively sparsely represented age groups such as children and older adults, we used robust regression. Because calculating the statistical significance of regression parameters in mixed linear models, and even more so in robust regression, is still somewhat controversial, we employed three strategies to assess the significance of the effects. First, we followed a recently used strategy (e.g., Geniole et al., 2019; Sleegers et al., 2021; Yiotis et al., 2021) and calculated *p* values based on *t* values estimated in robust regression and degrees of freedom estimated by regular regression. Second, we used recently evaluated wild bootstrap resampling (Mason et al., 2021) to estimate 0.95% confidence intervals for regression coefficients and considered those that did not contain 0 to be significant. Finally, we calculated Δ*R*^2^, *d*, and *f*^2^ to estimate the effect size of the factors of interest.

##### 2.3.3.3. Correlations With Cognitive Tests

To explore correlations of C3T measures with results of cognitive tests, we computed Pearson's correlations. To account for multiple comparisons, we adjusted and reported *p*-values using the FDR correction Benjamini and Yekutieli (2001) within each sample.

#### 2.3.4. Simulations

To better understand the nature of performance differences between stable and flexible task modes across the lifespan, we computed a series of numerical models that simulate possible causes of differences between the two task modes. As a starting point, we created a predictive model of the following form:


(4) $$\begin{array}{r} t_{p}=\alpha +\beta_{1}log\left(age\right)+\beta_{2}log{\left(age\right)}^{2} \end{array}$$

which roughly reflects the observed preparation times across the lifespan in the stable task mode. Next, we calculated the estimated preparation times and *SCI*_*t*_ by simulating the following possible drivers of change and their combinations: (i) a constant increase in time in the flexible task mode, (ii) a relative increase in preparation time in the flexible task mode, (iii) an earlier or later development (i.e., peak performance) of cognitive systems underlying flexible cognitive control compared to stable cognitive control.

All analyses and simulations were performed in R 4.1.0 (RCoreTeam, 2014), using the *lmer* and *glmer* functions of the *lme4* library (v4.1.1; Bates et al., 2015) for the analysis of linear and generalised linear mixed models, respectively, *lmrob* function from the *robustbase* library (v0.93-8; Maechler et al., 2021) and *rlmer* function from the *robustlmm* library (v2.4-4 Koller, 2016) to compute robust linear and robust linear mixed models, respectively. We used *CIrobustLMM* code (Mason et al., 2021) to compute bootstrap confidence intervals for coefficient estimates and *ez* library (v4.4-0; Lawrence, 2013) for computing analysis of variance. We visualised the results using the *ggplot2* library (v3.3.5; Wickham, 2009) and used TidyVerse (Wickham et al., 2019) set of libraries for data manipulation.

The full reproducible code and data are available in the Cognitive Control Challenge Task Open Science Foundation repository.

## 3. Results

To address the research questions, we divided the analyses and results into three sections. First, we examined the properties of the C3T to evaluate it as a test of stable and flexible cognitive control. Next, we used the results of the C3T to investigate the development of cognitive control across the lifespan. Finally, to validate the use of the C3T to assess change in cognitive control across the lifespan and to gain additional information about the cognitive processes involved in the task, we compared performance on the C3T with a number of standard tests of cognitive control.

### 3.1. C3T Differentiates Between Task-Set Formation, Maintenance, and Switching

#### 3.1.1. Accuracy Is Higher in Stable Compared to Flexible Task Mode

**Figure 3:**
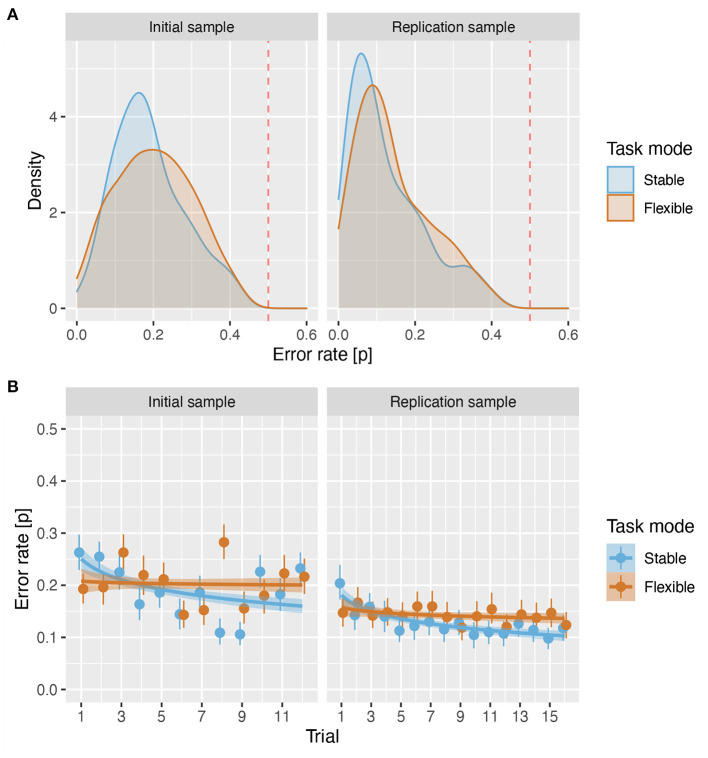
Error rates. **(A)** Density plot of error rates for both samples in stable and flexible task modes. The red line shows the error rate of 0.5. **(B)** Proportion of errors across all participants and rule types for each trial number. The circles show the mean error rate and the handles show the 95% confidence intervals. The lines show the predicted values based on a linear regression with trial and mode as predictors, and the shading shows the standard error of the predicted values.

First, we examined the distribution of error rates in initial and replication samples to determine how successful participants were in performing the task. The distributions of mean error rates per participant across all C3T trials (Figure 3A) showed that in both samples, the majority of participants performed the task well above chance both in the stable (IS: $\bar{err}=0.19$, *sd* = 0.093; RS: $\bar{err}=0.13$, *sd* = 0.099) as well as in the flexible (IS: $\bar{err}=0.20$, *sd* = 0.101; RS: $\bar{err}=0.14$, *sd* = 0.098) task mode, even suggesting a floor effect in the RS.

In the following analysis, we addressed two questions. First, whether task mode (stable vs. flexible) affects accuracy. Second, whether participants improved their accuracy over the course of the trials. To answer these two questions, we constructed a logistic regression model in which errors were predicted by task mode (stable vs. flexible) as a dichotomous variable and trial order as a continuous variable. To account for the general finding that the training effect is larger on initial trials and then reaches a plateau, we modelled the training effect as the natural logarithm of the trial number within each rule type. We also included task mode × trial order interaction in the model to account for differences in the training effect related to rule acquisition and application in the stable task mode and task set switching in the flexible task mode.

In both samples, the analysis revealed a significant effect of mode (IS: β = −0.187, *z* = −2.61, *p* = 0.009, *OR* = 0.83; RS: β = −0.144, *z* = −2.035, *p* = 0.042, *OR* = 0.87), reflecting slightly lower error rates in stable than in flexible task mode (Figure 3B), a significant overall effect of trial order (IS: β = −0.146, *z* = −5.00, *p* < 0.001, *OR* = 0.86; RS: β = −0.192, *z* = 7.12, *p* < 0.001, *OR* = 0.82), and a significant trial order × task mode interaction (IS: β = 0.152, *z* = 3.80, *p* < 0.001, *OR* = 1.16; RS: β = 0.133, *z* = 3.798, *p* < 0.001, *OR* = 1.14), which together reflect a robust effect of training on stable trials (IS: β = −0.250, *z* = −6.16, *p* < 0.001, *OR* = 0.78; RS: β = −0.293, *z* = −7.211, *p* < 0.001, *OR* = 0.75), which was absent on flexible trials (IS: β = −0.033, *z* = −0.802, *p* = 0.422, *OR* = 0.97), or significantly reduced (RS: β = −0.084, *z* = −2.38, *p* = 0.017, *OR* = 0.92). For details, (see Supplementary Tables 2–4).

#### 3.1.2. Preparation Times Reflect Task Set Formation and Task-Set Switching

In analyses of reaction times, we sought to answer three questions. First, is there evidence of task set formation when a participant is first confronted with a new task rule. Second, does the task allow for separate estimates of task set activation and task performance. Third, is there any evidence of task set switching cost. We answered these questions by reviewing and analysing preparation, response, and total reaction times. In addition, to control for and to examine the effects of different types of training—encoding and optimising the task set in the stable task mode and task switching efficiency in the flexible task mode—we observed changes in response times across progression of the task. In all analyses in this section, we used the median reaction times across all task rules at each trial number for the stable and flexible task modes separately.

**Figure 4:**
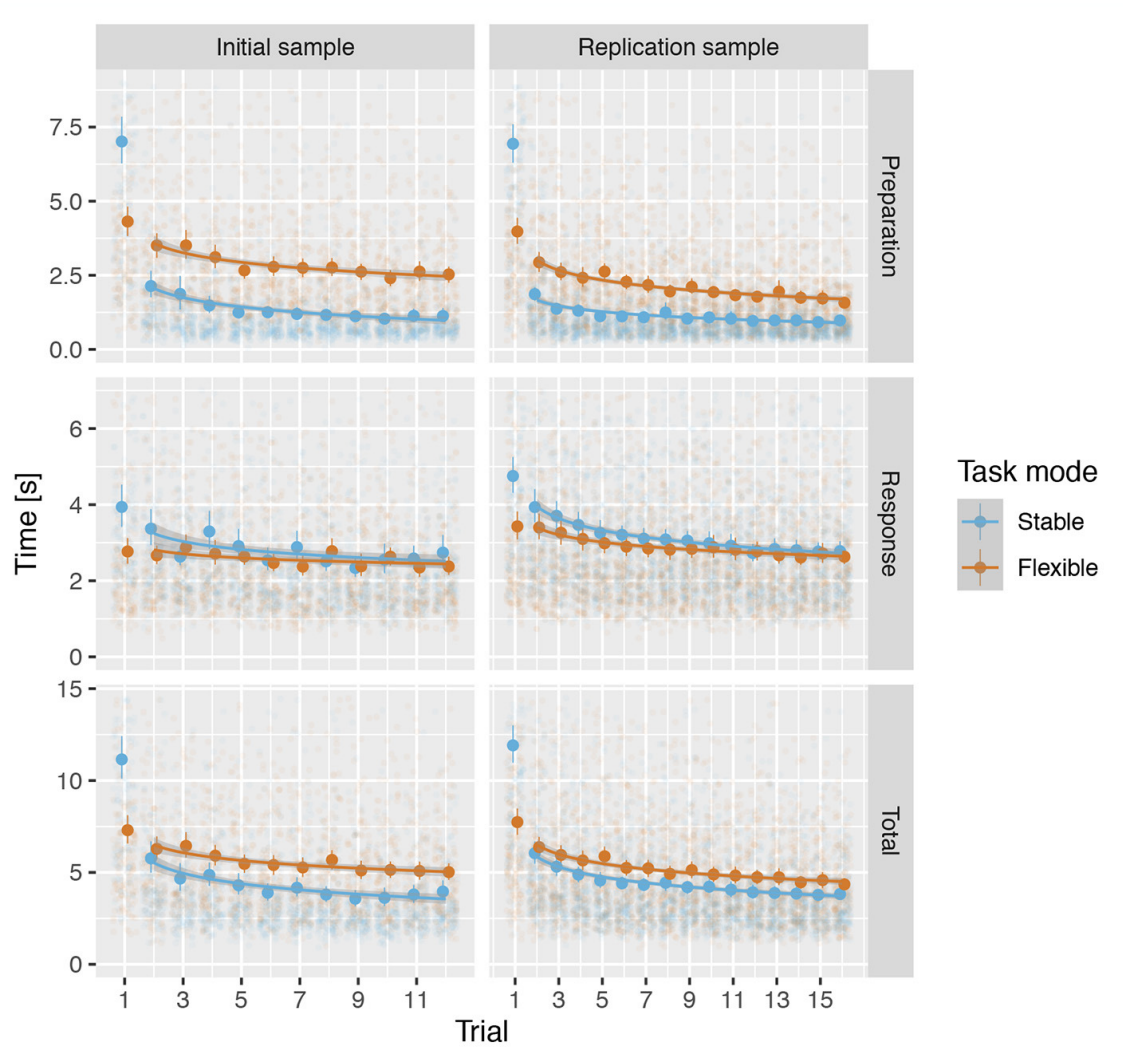
Median reaction times across all rules for each trial for both samples in the stable and the flexible task modes. The circles show the median reaction times and the handles show the 95% confidence intervals. The lines show the predicted values based on a mixed linear regression model and the shading shows the standard error of the predicted values.

Visual inspection of reaction times across trials indicated a robust effect of initial exposure to a task rule in the stable but not flexible task mode (Figure 4). To address the first question—is there evidence for task set formation when participant is first confronted with a new task rule—we used a mixed-model linear regression analyses with predictors trial (first vs. second), task mode (stable vs. flexible) and their interaction to predict preparation and response times. The analyses of preparation times revealed significant trial × task mode interaction in both IS: β = 2.890, *t*_(467.0)_ = 10.1, *p* < 0.001, *d* = 0.788, *f*^2^ = 0.078 and RS: β = 2.853, *t*_(547.9)_ = 10.7, *p* < 0.001, *d* = 0.910, *f*^2^ = 0.104. The effect was much less pronounced in response times, failing to yield a significant interaction in IS, β = 0.340, *t*_(467.1)_ = 1.67, *p* = 0.096, *d* = 0.128, *f*^2^ = 0.002, but still significant in RS: β = 0.561, *t*_(547.7)_ = 3.05, *p* = 0.002, *d* = 0.195, *f*^2^ = 0.005 (see Supplementary Tables 5–7 for details).

Due to the pronounced difference in reaction times to the first occurrence of a rule, we used and analysed it separately as setup time. We based all further analyses of reaction times in both stable and flexible task modes on trials two and more.

To assess the information provided by preparation, response, and total times, we used mixed-model linear regression analyses to obtain estimates of the effects of task mode (stable vs. flexible) and trial on median reaction time across task rules, separately for preparation, response, and total times. As with the accuracy analyses, we included trial in the models to control for and examine the effect of training. To account for the effect of training decreasing with time, we modelled the trial with a natural logarithm of the trial number (IS: 2–12, RS: 2–16). Again, to account for differences in the type of training in stable and flexible mode, we also included a regressor for task mode × trial interaction.

For preparation times, analyses revealed a significant main effect of mode in both IS and RS (see Table 3 and Supplementary Tables 8, 9), reflecting shorter preparation times in stable mode compared to flexible task mode (Figure 4). Moreover, in both IS and RS, the analysis revealed a significant main effect of trial, confirming a decellerated reduction in preparation time with each new trial of the same rule. In RS, the analyses also revealed a mode × trial interaction, reflecting a stronger effect of training in the flexible task mode than in the stable task mode, a difference that was absent in IS.

**Table 3:** Summary of hierarchical linear modeling analyses for preparation time, response time, and total time.

**Predictor**	**β**	**df**	**t-value**	**p-value**	***CI*_*lo*_**	***CI*_*hi*_**	**d**	***f* ^2^**	**sig**.
**Preparation time**								
**Initial sample**								
mode	1.077	2879.3	12.4	<0.001	0.765	1.369	0.454	0.089	^***^
trial	−0.457	141.3	−9.05	<0.001	−0.595	−0.342	−0.193	0.032	^***^
mode × trial	−0.010	2879.1	−0.200	0.841	−0.136	0.132	−0.004	0.000	
**Replication sample**								
mode	0.945	4864.7	15.3	<0.001	0.760	1.132	0.598	0.088	^***^
trial	−0.383	208.0	−14.8	<0.001	−0.454	−0.319	−0.242	0.054	^***^
mode × trial	−0.140	4864.4	−4.55	<0.001	−0.221	−0.068	−0.088	0.002	^***^
**Response time**								
**Initial sample**								
mode	−0.300	3082.3	−4.01	<0.001	−0.508	−0.133	−0.142	0.002	^***^
trial	−0.230	186.9	−6.78	<0.001	−0.310	−0.172	−0.109	0.006	^***^
mode × trial	0.104	3082.1	2.43	0.015	0.022	0.205	0.049	0.001	^*^
**Replication sample**								
mode	−0.403	5079.4	−7.31	<0.001	−0.562	−0.234	−0.160	0.002	^***^
trial	−0.366	182.7	−8.43	<0.001	−0.461	−0.292	−0.145	0.011	^***^
mode × trial	0.129	5079.3	4.71	<0.001	0.058	0.195	0.051	0.001	^***^
**Total time**								
**Initial sample**								
mode	0.693	3008.8	5.80	<0.001	0.347	0.997	0.166	0.023	^***^
trial	−0.715	153.7	−9.51	<0.001	−0.893	−0.566	−0.172	0.017	^***^
mode × trial	0.147	3008.6	2.15	0.031	−0.017	0.326	0.035	0.000	
**Replication sample**								
mode	0.440	5054.8	4.98	<0.001	0.174	0.700	0.124	0.010	^***^
trial	−0.764	182.9	−13.3	<0.001	−0.901	−0.644	−0.215	0.026	^***^
mode × trial	0.048	5054.6	1.08	0.280	−0.063	0.162	0.013	0.000	

*Degrees of freedom and p-values were established using Satterthwaite's method, CIs were estimated using wild bootstrap procedure, f^2^ was estimated using reduced models (see methods for details). Sig. codes are*

* *p <0.05*,

** *p <0.01*,

*** *p <0.001. When CI includes zero, the estimates were not considered significant*.

Analysis of response times similarly revealed a significant main effect of mode in both IS and RS (see Table 3 and Supplementary Tables 10, 11), this time reflecting somewhat longer response times in stable task mode than in flexible task mode (Figure 4). A significant effect of the trial again reflected a decellerated decrease in response times on successive trials in both IS and RS. Significant mode × trial interactions reflected a slightly stronger effect of training in stable task mode than in flexible task mode in both IS and RS.

The analysis of total times reflected the sum of preparation and response times. Linear mixed modelling confirmed a significant effect of mode (see Table 3 and Supplementary Tables 12, 13), reflecting the overall longer time required to complete trials in flexible task mode than in stable task mode (Figure 4). The significant effect of trial confirmed the overall increase in the speed at which trials were completed, which showed no significant interaction with mode in either IS or RS.

The observed pattern of results across preparation, response and total times supported the expectation that the task would (i) allow separate estimation of the preparation and response components of trial performance times, (ii) that preparation times would better reflect differences in task mode performance, and thus (iii) provide a more direct estimate of complex task set switching cost.

#### 3.1.3. Time-Based Derived Task Setup and Task Switching Measures Enable Robust Individual Level Performance Estimates

For a task to be useful as an instrument for assessing individual differences, it should include measures that provide direct estimates of key processes of interest. Moreover, such measures should not only show the expected group-level differences, but the effects should also be robust at the individual level. To assess the performance of C3T as a diagnostic tool, we computed and evaluated three derived measures, *STI*, *SCI*_*t*_, and *SCI*_*e*_ (see section 2 for details), and examined each of them to determine whether they show the expected effects at the individual level.

**Figure 5:**
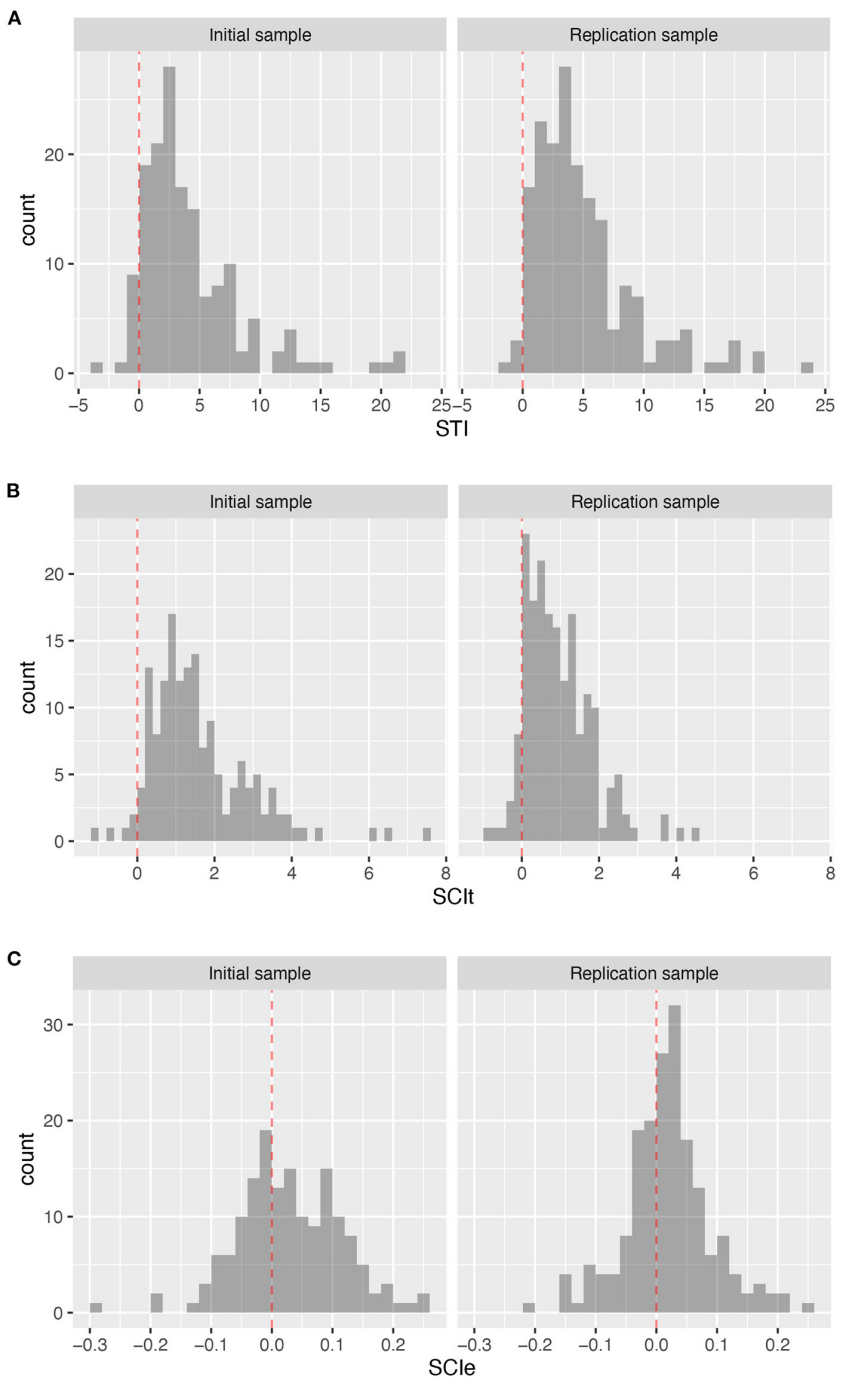
Distribution of derived measures at the individual level. **(A)** Distribution of the estimate of the setup time *STI*. **(B)** Distribution of preparation time based *SCI*_*t*_. **(C)** Distribution of error-based *SCI*_*e*_.

The results showed that *STI* provided a robust individual-level estimate of initial task set setup time (Figure 5A; SI, *Mdn* = 2.98, *CI* = [−0.82, 16.47]; RS, *Mdn* = 3.90, *CI* = [0.07, 17.21]), with only 7.0% and 2.2% of participants (IS and RS, respectively) having a task setup time estimate equal to or less than 0.

The *SCI*_*t*_ also provided a robust estimate of switching costs at the individual level (Figure 5B; SI, *Mdn* = 1.35, *CI* = [−0.09, 4.39]; RS, *Mdn* = 0.78, *CI* = [−0.28, 2.81]), with only 3.3 and 7.6% of participants (IS and RS, respectively) showing shorter average reaction times in flexible compared to stable task performance.

Finally, the analysis of *SCI*_*e*_ suggests that whereas group-level error rates are significantly higher in the flexible task mode than in the stable mode, this is not consistently the case at the individual level (Figure 5C; SI, *m* = 0.029, *sd* = 0.086; RS *m* = 0.020, *sd* = 0.072), with 36 and 33% of participants (IS and RS, respectively) showing the opposite pattern, namely higher error rates during stable rather than flexible task mode performance.

### 3.2. C3T Indicates Changes in Cognitive Control Components Across Lifespan

Having examined the internal validity of the C3T as a measure of stable and flexible cognitive control, we focused on investigating changes in cognitive control across the lifespan, more specifically from late childhood to late adulthood. We first explored task performance as indexed by accuracy and reaction times. Next, we examined derived measures of complex task set setup time and switching cost.

#### 3.2.1. C3T Performance Increases in Childhood and Gradually Declines in Adulthood

First, we examined the change in accuracy across the lifespan using logistic regression on correct vs. incorrect responses with the predictors age, task mode (stable vs. flexible), and their interaction as fixed effect variables and participants as random effect variables. Importantly, to account for the inverted U relationship between age and cognitive ability, characterised by a relatively faster increase in childhood and a slower decline with age, age was modelled as a second-degree polynomial of a natural logarithm of completed years of age.

**Figure 6:**
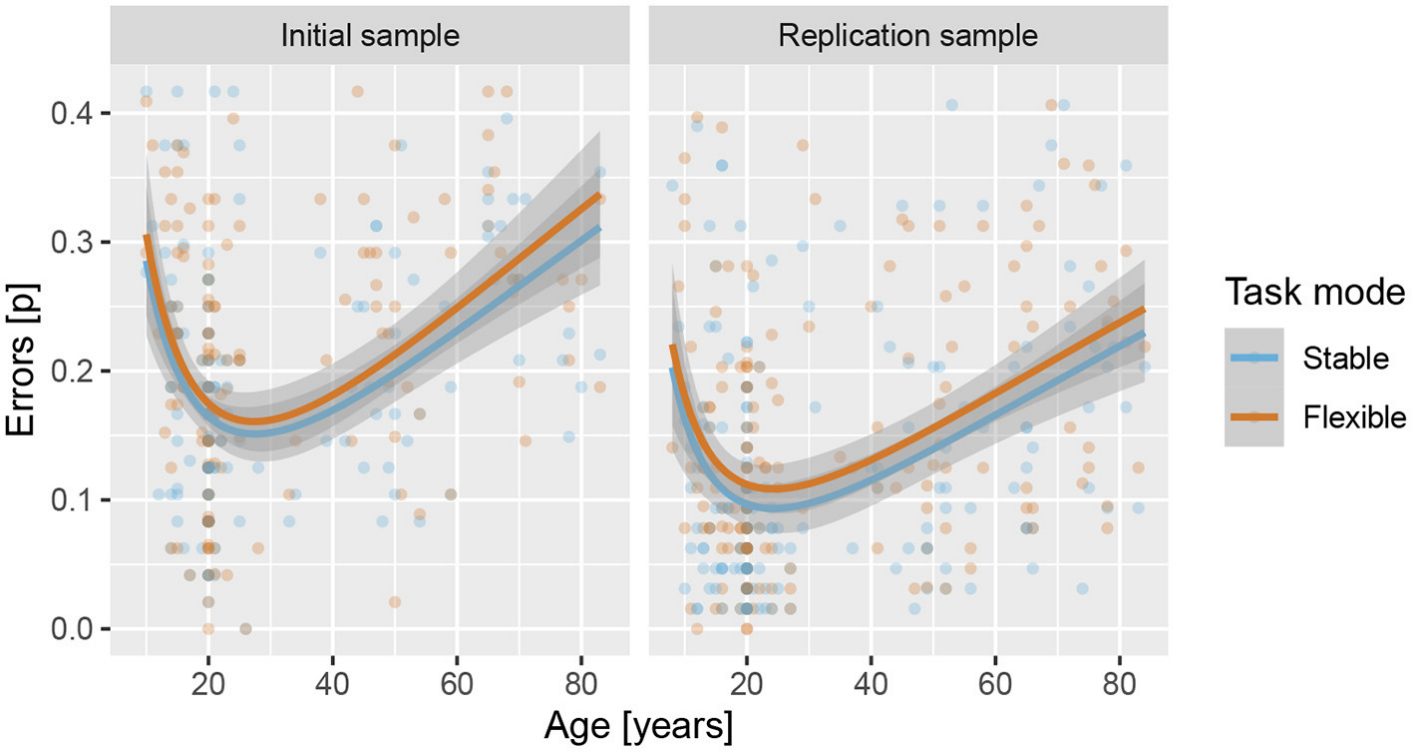
Error rates across lifespan for the stable and flexible task modes for both samples. Lines show predicted values based on linear regression, with age modelled as a second-degree polynomial of the logarithm of age in years, and shading shows the standard error of predicted values.

Results showed that adding regressors for age significantly improved the logistic regression model in IS, $\chi_{\left(2\right)}^{2}=33.4$, *p* < 0.001, *f*^2^ = 0.02, and RS, $\chi_{\left(2\right)}^{2}=34.2$, *p* < 0.001, *f*^2^ = 0.03, with significant β estimates for both linear and quadratic components (see Supplementary Tables 17–19 for details), reflecting an increase in task performance from late childhood to emerging adulthood and then a slower decline throughout adulthood (see Figure 6). Results also showed a significant effect of task mode in both IS, β = 0.059, *Z* = 1.96, *p* = 0.050, *d* = 0.12, *OR* = 0.11 and RS, β = 0.106, *Z* = 3.71, *p* < 0.001, *d* = 0.15, *OR* = 0.11, reflecting lower error rates for the stable than the flexible task mode. There was no indication of age × task mode interaction in either IS, $\chi_{\left(2\right)}^{2}=0.087$, *p* = 0.957 or RS, $\chi_{\left(2\right)}^{2}=0.376$, *p* = 0.828.

**Figure 7:**
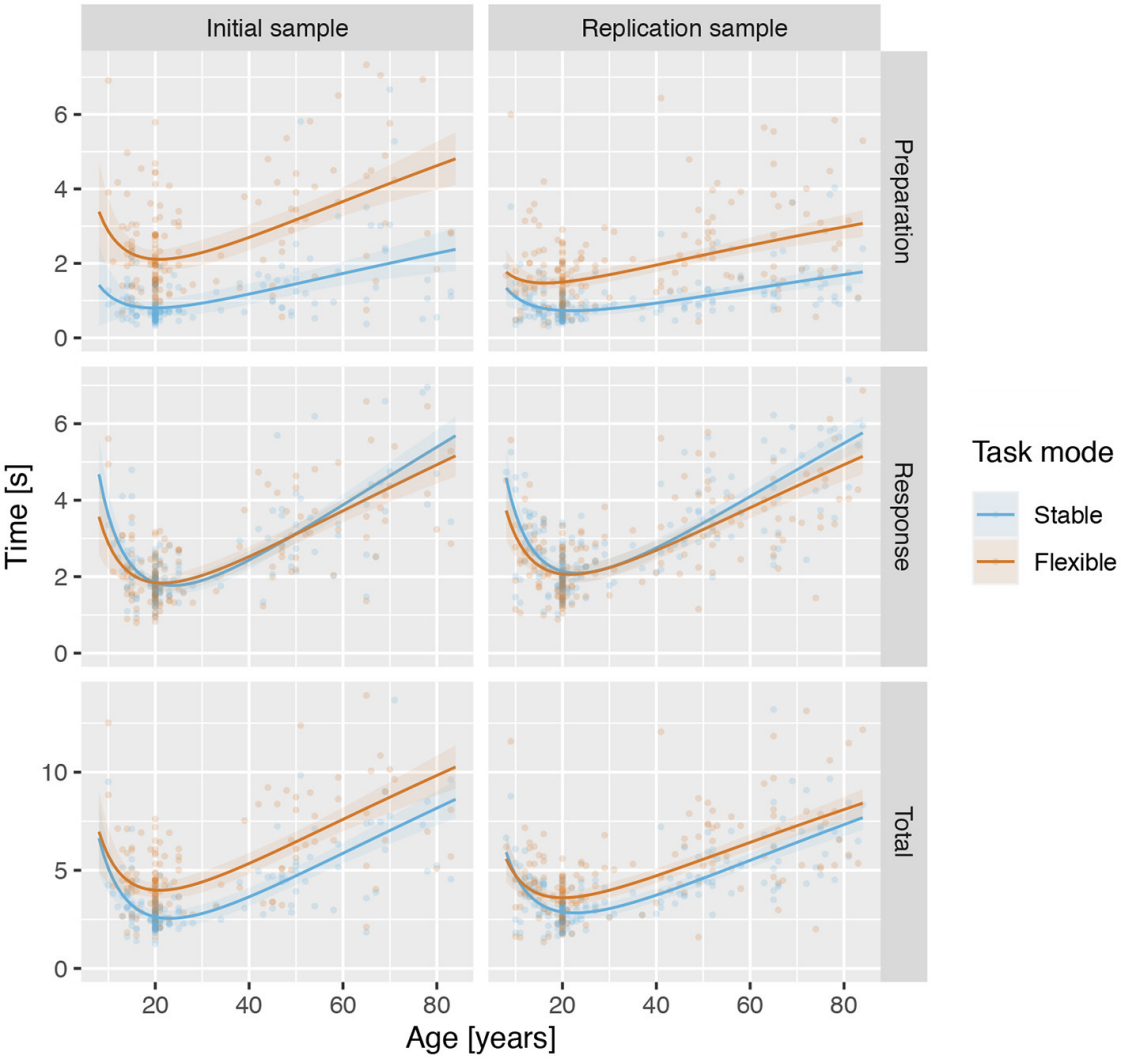
Reaction times across lifespan for the stable and flexible task modes for both samples. Lines show predicted values based on robust linear regression, with age modelled as a second-degree polynomial of the logarithm of age in years, and shading shows the standard error of predicted values.

Next, we explored the changes in preparation, response and total times across the lifespan using robust linear regression with age, task mode (stable vs. flexible), and their interaction as fixed variables and participant as a random-effect variable. For all three measures of reaction times in both samples, the analysis revealed a significant effect of both the linear and quadratic components of age (see Table 4), again reflecting a U-shaped relationship between task performance and age across the lifespan, with a decrease in reaction times from late childhood to emerging adulthood, followed by a consistent increase throughout adulthood (see Figure 7). Results also confirmed significantly longer preparation and total times in the flexible task mode, whereas response times were slightly longer in the stable task mode. In RS, there was also evidence of age × mode interaction. Specifically, the difference between preparation times in the stable and flexible task modes increased linearly with age, whereas differences in response times were associated with the quadratic component of age—they were smallest during emerging and young adulthood and more pronounced in both younger (late childhood and adolescence) and older (middle and late adulthood) participants.

**Table 4:** Summary of robust hierarchical linear modeling analyses for preparation time, response time, and total time across lifespan.

**Predictor**	**β**	**df**	**t-value**	**p-value**	***CI*_*lo*_**	***CI*_*hi*_**	**d**	***f* ^2^**	**sig**.
**Preparation time**								
**Initial sample**								
age	8.762	153	7.13	<0.001	4.422	13.487	7.674	0.307	^***^
age^2^	4.863	153	4.28	0.001	1.519	8.516	4.260		^**^
mode	1.062	153	16.1	<0.001	0.932	1.203	0.930	0.235	^***^
age × mode	2.985	153	2.46	0.004	−0.573	6.604	2.615	0.018	
age^2^× mode	2.054	153	2.87	0.045	−1.267	6.240	1.799		
**Replication sample**								
age	6.509	181	6.76	<0.001	3.780	9.078	8.706	0.307	^***^
age^2^	3.742	181	3.89	<0.001	1.274	6.990	5.006		^***^
mode	0.618	181	16.8	<0.001	0.531	0.714	0.826	0.191	^***^
age × mode	3.063	181	4.33	<0.001	1.159	5.029	4.097	0.045	^***^
age^2^× mode	0.019	181	0.026	0.979	−1.773	1.850	0.025		
**Response time**								
**Initial sample**								
age	13.072	153	10.3	<0.001	8.451	19.082	14.291	1.178	^***^
age^2^	9.618	153	7.56	<0.001	5.441	14.705	10.515		^***^
mode	−0.062	153	−2.05	0.042	−0.209	0.060	−0.068	−0.003	
age × mode	−0.072	153	−0.136	0.892	−4.470	3.191	−0.079	−0.010	
age^2^× mode	−2.153	153	−4.04	<0.001	−5.702	1.251	−2.353		
**Replication sample**								
age	13.730	181	9.46	<0.001	9.726	18.371	13.222	1.038	^***^
age^2^	12.364	181	8.52	<0.001	8.975	16.127	11.906		^***^
mode	−0.140	181	−4.73	<0.001	−0.215	−0.063	−0.134	0.013	^***^
age × mode	−0.568	181	−1.00	0.318	−2.600	1.405	−0.547	0.009	
age^2^× mode	−2.361	181	−4.16	<0.001	−3.978	−0.721	−2.273		^***^
**Total time**								
**Initial sample**								
*age*	23.355	153	9.47	<0.001	14.969	33.024	12.761	0.789	^***^
age^2^	15.613	153	6.33	<0.001	8.693	23.773	8.531		^***^
mode	1.000	153	12.7	<0.001	0.751	1.224	0.547	0.201	^***^
age × mode	2.750	153	1.98	0.049	−4.387	9.067	1.502	0.008	
age^2^× mode	−1.265	153	−0.911	0.364	−7.132	6.377	−0.691		
**Replication sample**								
age	21.108	181	9.99	<0.001	15.214	27.624	13.670	0.907	^***^
age^2^	16.476	181	7.79	<0.001	11.061	22.990	10.670		^***^
mode	0.505	181	9.26	<0.001	0.373	0.637	0.327	0.067	^***^
age × mode	2.439	181	2.33	0.021	−0.861	5.518	1.579	0.029	
age^2^× mode	−2.220	181	−2.12	0.035	−4.955	0.783	−1.438		

*Degrees of freedom were established using Satterthwaite's method for the regular regression and applied to compute p-values based on t-values established using robust regression, 95% CIs were estimated using wild bootstrap procedure, f^2^ was estimated using reduced models (see methods for details). Sig. codes are*

* *p <0.05*,

** *p <0.01*,

*** *p <0.001. When CI includes zero, the estimates were not considered significant*.

#### 3.2.2. Complex Task Set Setup Time and Switching Cost Increase From Late Childhood Throughout Lifespan

To examine changes in the ability to set up and switch between complex task sets across the lifespan, we investigated three derived measures, the Setup Time Index (*STI*), the error-based Switching Cost Index (*SCI*_*e*_), and the preparation-time-based Switching Cost Index (*SCI*_*t*_). In all three cases, we used robust linear regression with age as the predictor. As before, we modelled age as a second-degree polynomial of the logarithm of age in years.

**Figure 8:**
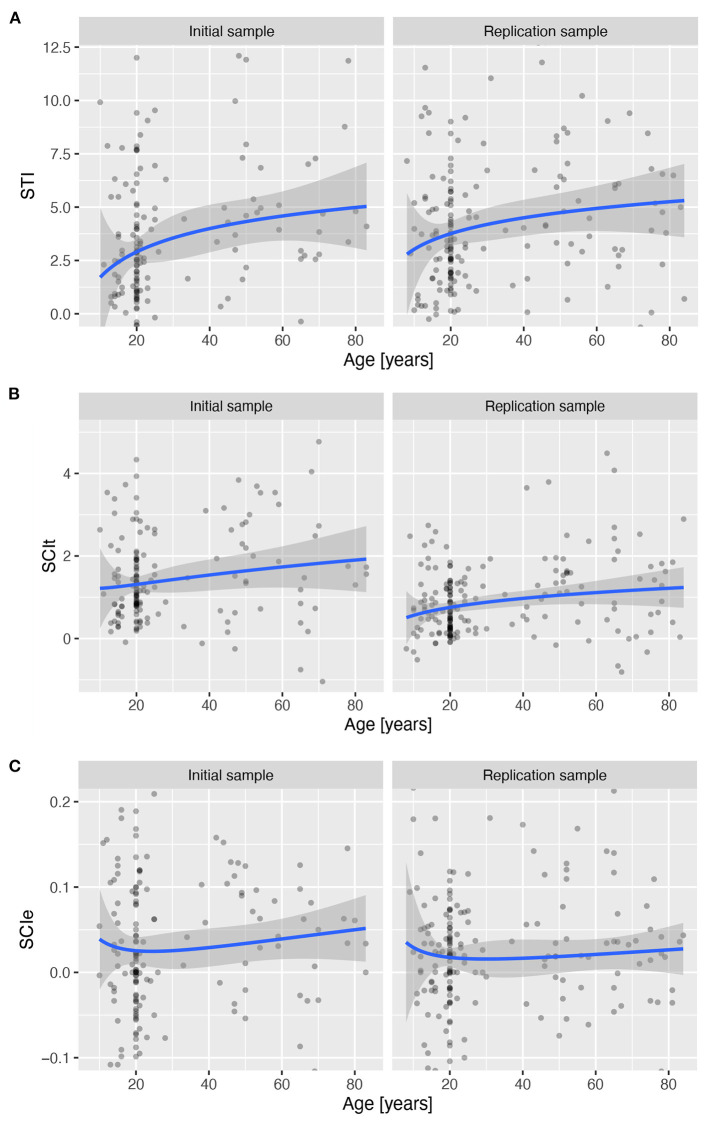
Three derived measures across lifespan for the stable and flexible task mode over age for both samples. Lines show the predicted values based on a robust linear regression with age modelled as a second-degree polynomial of the log of age in years, and shading the standard error of the predicted values. **(A)** Setup Time Index (*SCI*_*t*_). **(B)** Error based Switching Cost Index (*SCI*_*e*_). **(C)** Preparation time based Switching Cost Index (*SCI*_*t*_).

Investigation of the *STI* revealed a significant linear increase with age in both IS, β = 10.4, *t*_(153)_ = 2.92, *p* = 0.004, and RS, β = 8.82, *t*_(177)_ = 2.24, *p* = 0.027, whereas the quadratic component was not statistically significant in either IS, β = −0.34, *t*_(153)_ = −0.076, *p* = 0.939, or RS, β = 0.055, *t*_(177)_ = 0.014, *p* = 0.989, which together indicate an increase in the time required to establish a complex task set from late childhood throughout lifespan (see Figure 8A and Supplementary Table 20).

Investigation of the *SCI*_*t*_ suggested a slow linear increase in switching cost across the lifespan (see Figure 8B), which was significant in RS, β = 2.63, *t*_(153)_ = 2.483, *p* = 0.014, but not in IS, β = 2.415, *t*_(181)_ = 1.576, *p* = 0.117. However, there was no evidence of a quadratic relationship with age (see Supplementary Table 21 for details).

The *SCI*_*e*_ did not show a reliable pattern of change across the lifespan with either a linear or a quadratic component of the age predictor (see Figure 8C and Supplementary Table 22).

We compared the observed pattern of differences in *STI* and *SCI*_*t*_ with simulations of different possible causes of differences between performance in stable and flexible task modes. The empirical results agreed best with the simulation that assumed both an absolute and relative increase in preparation time in the flexible task mode, as well as an earlier development of peak performance in the flexible task mode (see Supplementary Figure 6 for details).

### 3.3. C3T Relates to Other Measures of Cognitive Control

The last topic we addressed in the results is the extent to which the measures obtained in C3T are related to other tasks and tests of cognitive control. To this end, we computed correlations between performance times and accuracy in stable and flexible modes and the three derived measures (*STI*, *SCI*_*t*_, and *SCI*_*e*_) with participants' results on tests of working memory span (WM), trail making test (TM), verbal fluency (VF), Tower of London (TOL), and operational span (OSPAN).

**Figure 9:**
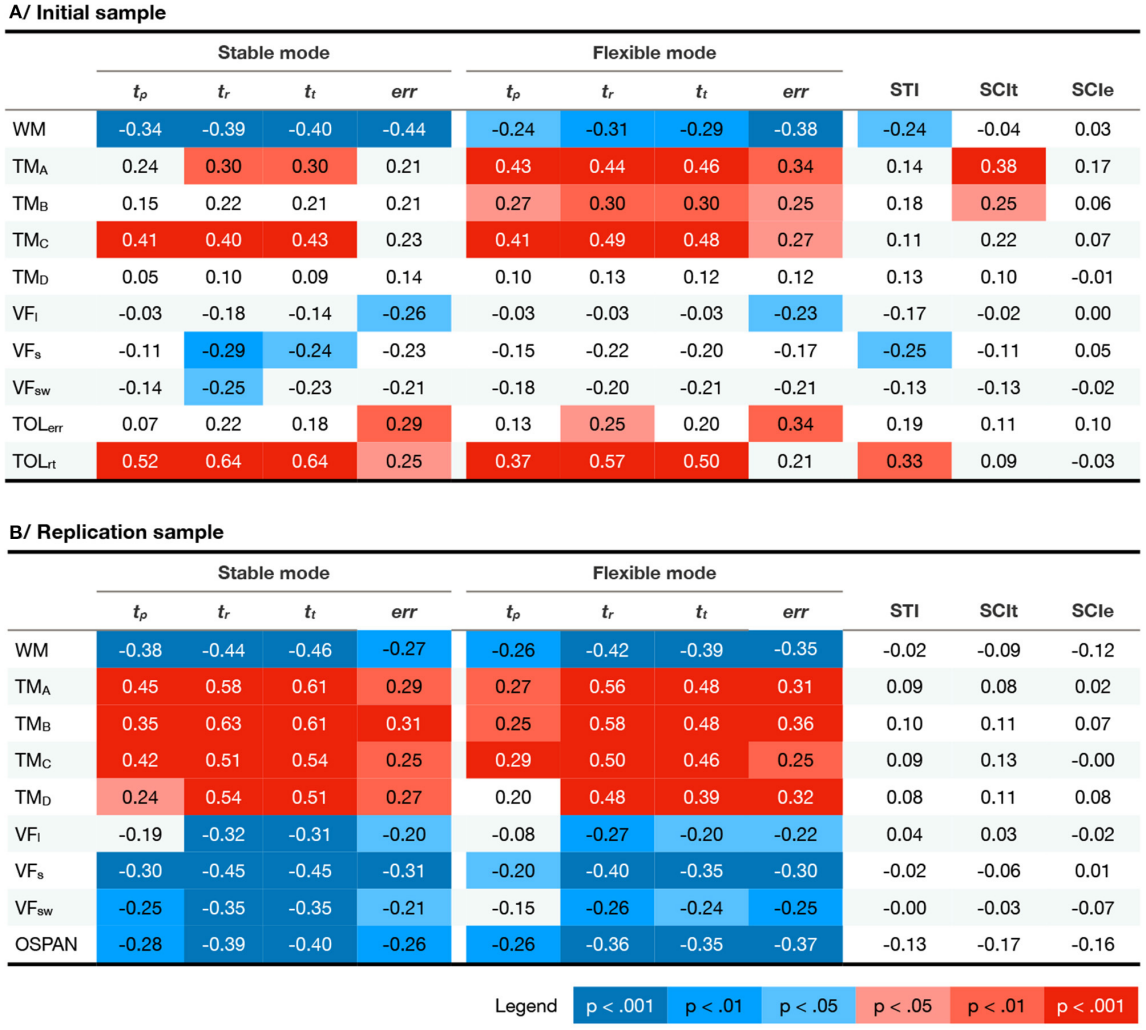
Correlations between C3T measures and standard tests of cognitive control in the stable and flexible modes for both samples. Legend: *t*_*p*_, preparation time; *t*_*r*_, response time; *t*_*t*_, total time; *err*, error rate; *STI*, Setup Time Idex; *SCI*_*t*_, preparation-time-based Switching Cost Index; *SCI*_*e*_, error-rate-based Switching Cost Index; *WM*, working memory span; *TM*_*A*_, time to complete Trail Making task part A; *TM*_*B*_, time to complete Trail Making task part B; *TM*_*C*_, time to complete Trail Making task part C; *TM*_*D*_, time to complete Trail Making task part B subtracted by time to complete Trail Making task part A; *VF*_*L*_, lexical verbal fluency; *VF*_*S*_, semantic verbal fluency; *VF*_*S*_*W*, category switching verbal fluency; *TOL*_*e*_*rr*, Tower of London error rate; *TOL*_*r*_*t*, Tower of London average response time; *OSPAN*, operational span. The colors denote significant positive (red) and negative (blue) correlations controlled for multiple comparisons using FDR correction.

Results (see Figure 9 and Supplementary Figures 7–11) showed significant correlations of working memory measures, both simple (WM) and complex span (OSPAN) with C3T performance times and accuracy in both samples, reflecting shorter reaction times and lower error rates in individuals with higher working memory capacity. Results also indicated significant associations with TM measures. In the IS, significant correlations were mostly limited to parts A and C for the performance measures in the stable task mode and also to part B for the measures in the flexible task mode. In RS, significant correlations were found with TM for both stable and flexible task mode performance. In all cases, the relationship was as expected: individuals with faster TM performance also showed faster and more accurate performance on the C3T.

Measures of verbal fluency showed a varied pattern of significant correlations. All VF variants showed the expected direction of the relationship, namely that individuals with higher VF performed the C3T faster and more accurately. However, in IS significant correlations were observed mainly between stable task mode measures and the semantic version of the VF. In RS, again, the relationship between semantic VF and stable task mode performance was most robust, although it was also observed between switching and lexical versions of the VF task and C3T reaction times.

Finally, C3T performance measures showed robust moderate correlations with TOL. Participants who were faster on TOL were also faster on C3T, and those who were more accurate on TOL also had higher accuracy on C3T.

Interestingly, the significant correlations were limited to the pure performance measures. Of the derived C3T measures, only *STI* in IS showed the expected pattern of significant correlations with cognitive test scores, and *SIC*_*t*_ correlated significantly with *TM*_*A*_ and *TM*_*B*_. In RS, the derived measures were not correlated with the results of the cognitive tests, indicating that they provide unique information.

To enable more detailed comparison with the C3T we have also plotted robust regression with age across the lifespan for all cognitive measures. The results are available in the Supplementary Material.

## 4. Discussion

In this study, we examined the performance of the newly developed Cognitive Control Challenge Task (C3T), first, to validate it as a measure of cognitive control, second, to investigate the development of the ability to encode, maintain and flexibly switch between complex rules and related task sets across the lifespan, and third, to investigate to what extent the two aspects of complex cognitive control—stable maintenance and flexible switching—reflect the capabilities of separable vs. common systems.

### 4.1. C3T Captures Task Performance and Task Set Encoding, Maintenance, and Switching

The observed pattern of results suggests that C3T enables separate estimation of the time required for participants to initially encode the task set, to update the task set, and to switch to another previously encoded task set. During the stable task mode, preparation time decreased significantly from the first to the second trial and improved only slowly on subsequent trials, suggesting improvement in the encoding of the rule (Figure 4, top row). Because participants were familiarised with the task and trained on the examples of different rules before testing began, we believe that the preparation time for the first trial of a rule reflects the formulation of the task set. Impressively, a single encounter with the rule was sufficient to encode the task. Preparation time on subsequent trials was often not long enough to completely reread the rule, suggesting that participants were able to stably maintain the encoded task set across trials.

Preparation times in the flexible task mode were much longer, even though the rules were already well-practiced. This suggests the presence of a switching cost, similar to the results of traditional switching tasks (Kray and Lindenberger, 2000; Kray et al., 2004; Dibbets and Jolles, 2006). Part of the time increase could be due to reading the rules to identify them. However, because only four well-practiced rules were used, they was easy to recognise without thoroughly reading the instructions. To distinguish more clearly between the time required to identify a rule and the time required to switch to the correct task set, we would need to interleave trials with repeated and changed rules—a way to further improve the task. The slow but steady decrease in average preparation time across trials in flexible mode most likely reflects both greater ease of rule identification and an effect of practice on the effectiveness of task set switching.

The response times showed a different pattern of results (Figure 4, middle row). Although there was a small but consistent improvement during repeated task execution from the first trial on, indicating an improvement in rule execution, there was no indication of significantly increased task execution time when the rule was first introduced. Furthermore, only a minimal difference was observed between the stable and flexible task modes, with response times being slightly shorter in the flexible task mode than in the stable task mode, even though the latter required a change in the task set at each trial. In our opinion, this is a strong indication that C3T successfully distinguishes between the times for encoding the task set, refreshing the task set, or switching between task sets, as indexed by preparation time, and the time for processing the stimuli and executing the task set, as indexed by response time. In other words, participants only continued to stimulus presentation after establishing the relevant task set.

To further validate C3T measures, we also checked for possible confunding influence of a speed-accuracy trade-off or a preparation-performance trade-off. We did not find evidence of either of these trade-offs either across or within individuals (for details see Supplementary Material).

Based on these findings, we computed and tested three derived measures of cognitive control. A setup time index *STI*, and two switching cost indices based on preparation time *SCI*_*t*_ and error rate *SCI*_*e*_. While investigation of *SCI*_*e*_ suggests that it is not a reliable measure of switching cost (see Figure 5C), *STI* and *SCI*_*t*_ can be considered measures reflecting the ability to establish complex task sets and the ability to flexibly switch between them, respectively.

Taken together, we believe that these results suggest that C3T can serve as an instrument to measure complex task set encoding, maintenance, and switching.

### 4.2. Task Performance on C3T Through Lifespan Follows a U-Shaped Developmental Curve

Lifespan studies frequently show systematic, age-related improvements in childhood and adolescence and declines in ageing in a variety of basic cognitive measures, such as processing speed and short-term memory (Zelazo et al., 2004), the ability to adopt and modify a problem-solving set (Jurado and Rosselli, 2007), and switching cost (Cepeda et al., 2001). We have observed the same pattern of development of cognitive abilities on all standard tests of cognition in our samples (see Supplementary Material). The basic performance measures on the C3T task show a similar progression throughout the lifespan. Specifically, the C3T measures of task accuracy, preparation, response, and total times both in stable and flexible task modes all follow the U-shaped developmental curve. These results suggest that the C3T provides a measure of complex cognitive control that is sensitive to developmental changes across the lifespan.

### 4.3. Stable and Flexible Cognitive Control Follow Different Developmental Paths

Although the mechanisms supporting stable and flexible cognitive control have been associated with different brain systems, the question remains whether these are two separable and independent abilities or whether flexible cognitive control should be considered a general property of cognitive processes (Ionescu, 2012). We approached this question by examining the extent to which lifespan changes in C3T measures of flexible and stable task performance follow the same developmental trajectory. Since the results showed that response times primarily reflect task set execution and do not distinguish between stable and flexible task modes, we focused our analyses primarily on preparation times and their derived measures.

While performance in stable and flexible task modes follows similar developmental trajectories (Figure 7), a more detailed comparison of the developmental trajectory of performance in different task conditions allows further investigation of the interdependence of stable and flexible cognitive control. Indeed, developmental studies have provided compelling evidence that the development of cognitive control functions is a multistep process in which different components develop at different times, beginning in infancy and continuing through adolescence and beyond (Welsh and Pennington, 1988). Similarly, our data suggest differences in development of stable and flexible cognitive control across the lifespan. More specifically, differences between performance in stable and flexible cognitive control continuously increase from late childhood onward. This is true for both the switching cost index (*SCI*_*t*_), and the setup time index (*STI*).

The nature of the differences can be better understood by comparing the empirical results with numerical simulations of the various possible causes. If stable and flexible cognitive control reflect a function of a common system, then we would expect the differences between performance under the different task conditions to be additive, multiplicative, or a combination of both. If the difference were only additive, that is, if preparation in the flexible task mode and construction of a new task set required a fixed amount of additional time, then we would expect the indices to be flat across the lifespan (Supplementary Figure 6A). More realistically, the difference would not be constant but would vary across the lifespan due to changes in processing speed. This would be reflected in an increase in preparation time that is linearly related to preparation time in stable task mode. In this case, the change in observed indeces across the lifespan should follow the shape of the developmental trajectory of preparation time (Supplementary Figure 6B). However, this is not the case. Both *SCI*_*t*_ and *STI* show a slightly decelerated increase throughout the lifespan.

The observed changes in the two flexible cognitive control indices can be predicted by assuming that the flexible cognitive control performance, such as task set switching in the flexible task mode and task set construction in the first trial, reflects the functioning of separable cognitive systems with somewhat different developmental characteristics across the lifespan. Specifically, the observed changes can be simulated by a combination of a relative increase in preparation time and earlier development of the cognitive control processes underlying flexible cognitive control compared to stable maintenance of cognitive control (Supplementary Figure 6E).

Another possibility would be that flexible cognitive control indices reflect an engagement of a separate cognitive process or ability with a specific developmental trajectory. Given our empirical data, we find this highly unlikely, as such an ability would have to reach its peak performance and start declining before late childhood. This would be substantially different from all other cognitive processes observed in this study (see Supplementary Figures 7–11).

Considering previous findings that 4-year-olds can already switch between abstract rules (e.g., Diamond, 1996; Bub et al., 2006), whereas children struggle to hold on to a relevant task set (Deák et al., 2004; Carroll et al., 2016), we believe that the assumption that the observed differences between flexible and stable task performance reflect the operation of two separable cognitive control systems with different developmental trajectories provides the most plausible explanation for the empirical data.

The observed delayed development of stable vs. flexible cognitive control is consistent with the developmental progression from reactive to proactive mode of cognitive control (Braver, 2012). Namely, proactive cognitive control depends on the ability to stably maintain a proactively activated task set. If task set maintenance skills develop more slowly, children may be limited in their ability to exercise proactive cognitive control. This would reduce the advantage of the stable task mode and lead to the observed lower switching cost in the youngest participants. Future studies could provide further insight into the extent to which the delayed development of proactive vs. reactive control observed in children (Chatham et al., 2009) and adolescents (Andrews-Hanna et al., 2011) may reflect the same developmental processes underlying the delayed development of stable vs. flexible complex cognitive control observed in this study.

### 4.4. Task Switching Costs Measures Across the Lifespan

The complex task switching cost observed in C3T and its changes across the lifespan compare interestingly with the previous literature on task switching. In particular, a meta-analysis of simple task switching tasks by Wasylyshyn et al. (2011) showed a significant U-shaped effect of age on global switching cost, defined as the difference in performance in pure vs. mixed blocks, but no age effects on local switching cost, defined as the difference in performance on switch and repeat trials within mixed blocks. The effect of age on global switching cost could not be attributed to global processing speed (Span et al., 2004), but to the ability to retain and coordinate two task sets in working memory (e.g., Kray and Lindenberger, 2000; Wasylyshyn et al., 2011).

Given the study design, we can assume that our data primarily capture the global switching cost and should therefore show a U-shaped developmental trajectory. However, in contrast to the literature on simple task switching and switching cost calculated using the TM data collected in our study (Supplementary Figure 7, bottom row), the complex task switching cost obtained using C3T (*SCI*_*t*_) show only a linear increase in late childhood and thereafter. In our opinion, this discrepancy can be attributed to differences in task complexity and associated cognitive demands. In particular, the simple switching tasks used in previous studies required minimal working memory demands to support task performance on stable trials and provide an advantage over switch trials as early as late childhood. In this context, the global switching cost can be largely explained by the different demands on working memory under the switch and stable conditions, as proposed in the existing literature (e.g., Kray and Lindenberger, 2000; Wasylyshyn et al., 2011).

In our simulation, the observed developmental trajectory of the cost of switching between simple tasks is congruent with two models. First, with a model that assumes that switching cost reflects a single capacity (Supplementary Figure 6B), e.g., working memory, which is more taxed in switching than in stable task blocks. A second possibility is that switching cost reflects both the capacity for flexible cognitive control required to switch between two task sets and a somewhat developmentally delayed working memory capacity. The combination of both would lead to a more pronounced U-shaped relationship with age (Supplementary Figure 6F).

In comparison, a task set in C3T consisted of three rules, which already placed a considerable load on working memory in the stable task mode. Moreover, in the flexible task mode, participants had to switch between four different task sets, which practically eliminates the possibility of retaining them all in working memory. In effect, all participants in the flexible task mode had to rely on some form of episodic retrieval, long-term working memory strategies, or task set recall. This leads to two consequences. First, due to the high load on working memory in the stable condition and the late development of working memory capacity, the stable task mode offered little advantage over the flexible task mode, which reduced the overall switching cost. Second, the difference in performance between the flexible and stable task modes, as indexed by *SCI*_*t*_, does not reflect differences in working memory capacity as otherwise assumed for simple switching cost. This is also supported by the presence of a significant correlation between working memory and performance measures in the flexible and stable task modes, and by the absence of a significant correlation between *SCI*_*t*_ and working memory (see Figure 9).

In summary, the design of the C3T allowed us to explicitly separate preparation times from execution times, so that neither stimulus ambiguity nor response conflict could be reflected in or influence the obtained estimate of the effect of task switching on preparation time. It could be argued that because of the explicit separation of preparation and execution time, the increased load on working memory in both stable and flexible task modes, and the absence of repeat trials in flexible mode, the comparison of preparation times between the two task modes yields an estimate of the cost of complex task switching that better fits the estimates of local rather than global switching costs in previous studies. However, this would need to be explicitly tested by the introduction of repeat trials in flexible task mode.

### 4.5. C3T Engages Multiple Systems and Provides a Measure of Complex Cognitive Control

While cognitive control is often defined as “our capability for directing thoughts and actions in accordance with internal goals, and for flexibly readjusting these goals when necessary” (Braver and Ruge, 2001), classic tests of cognitive control are paradoxically highly structured and require rigid test conditions (Burgess, 1997; Chan et al., 2008). This minimises the requirements for development and flexible switching between task sets, which is arguably the essence of cognitive control. The newly developed C3T task measures cognitive control in a less structured and thus more ecologically valid manner. Correlations of C3T measures with standard tests of cognitive control indicate that C3T performance across the lifespan relates to multiple processes, including working memory, planning, selective attention, inhibition and integration of information, semantic retrieval and task switching aspects of cognitive control, suggesting its use as a complex measure of these cognitive control abilities.

Although C3T makes it more difficult to assess exactly which processes are affected in a particular individual, group, or—as in this study—across the lifespan, it provides a measure of complex cognitive control that better reflects the ability to adapt and integrate individual cognitive control processes when faced with complex tasks in everyday situations.

### 4.6. Limitations and Relevance

For a comprehensive evaluation of C3T, some specific characteristics and limitations need to be considered. First, because the task is not very intuitive and requires the formation of complex task sets, administering the task requires potentially time-consuming explanation and practice of the task. However, once the general framework is understood, the C3T is completed without additional experimenter engagement. Furthermore, due to the different stimuli and complexity of the rules, participants consistently report that the task is fun and engaging. Nevertheless, a number of participants had to be excluded from the analysis due to low task accuracy. In the initial sample, the youngest and oldest participants had the most problems with the task (see Supplementary Table 1). The proportion of excluded participants decreased significantly in the replication sample, and differences between age groups were much less pronounced, likely due to a change in two of the rules and improved visual presentation of stimuli. For studies focusing on children and older adults, additional changes to the task design and increased attention to participants' understanding of the task could be considered.

The version of the C3T task studied makes a clear distinction between the stable and flexible task modes. In the stable mode, participants perform a longer series of trials with the same rule, while in the flexible mode the rules change from trial to trial. Moreover, the order of stable and flexible task mode blocks is held constant. This allowed us to focus on the process of task set setup and optimisation in the stable blocks and to obtain pure estimates of the time required to switch between well-learned and practiced task sets. The fixed block order, however, introduces potential problems and limitations related to the possible effects of practice and fatigue. The effect of practice could lead to relatively poorer performance in the stable task mode, leading to an underestimation of the switching cost compared to the flexible task mode. This possibility can be observed in the decrease in error rates in stable task mode trials compared to relatively constant error rates across flexible task mode trials (Figure 3B). Indeed, this may have resulted in lower average response times in flexible mode than in stable mode (Figure 4) and *SCI*_*e*_ being an unreliable derived measure (Figure 5C). Because of the relative strength of the effects of training and task mode, the problem is less pronounced when comparing preparation times and the resulting *SCI*_*t*_.

The design of the task could be improved by adding mixed mode blocks or changing flexible mode blocks to mixed mode blocks with pseudorandomly interspersed repeat and switch trials, as this would allow a more direct comparison of accuracy, preparation, and execution times between switch and repeat trials. In addition, the comparison of switch and repeat trials would allow a clearer distinction between the time to identify a rule and the time to switch to the correct task set. A mixed block of repeat and switch trials would also provide a better estimate of local and global switch costs (Wasylyshyn et al., 2011).

Although both the original and replication samples are relatively large, the uneven distribution of age, education, and gender in each sample and the relatively small number of participants in the late childhood group are important limitations of the study. However, the number of participants in the age groups is comparable to similar studies investigating specific cognitive processes such as the binding of information in working memory (15 participants per age group; Peich et al., 2013), inhibitory efficiency in working memory (30, and 28 younger and older adults, respectively; Blair et al., 2011), and mechanisms and limitations of visual working memory capacity (Slana Ozimič and Repovš, 2020), which included a similar sample of participants. The late adulthood age group is an additional concern, as the age effect may be confounded by underlying neuropsychological disorders. While we did not use screening tests, the absence of participants with marked and consistently below-average performance on psychological tests (see Supplementary Material for details) makes it unlikely that the results in this age group are confounded by undiagnosed neuropsychological disorders. Importantly, the reliability of the results is supported by their consistency across the two samples despite minor differences in task rules and the size of visual stimuli. This leads us to believe that while the results do not provide normative information, they do offer valuable insight into the development of flexible and stable cognitive control across the lifespan. Future studies with larger samples and better representation of different age groups would allow for a more focused investigation of complex cognitive control at different developmental stages and its relationship to other cognitive abilities.

Another consideration when using C3T with children is the extent to which it might interact with different levels of literacy. The words used in the task are common and well-known, so the results should not be affected by vocabulary. However, preparation and response times could be affected by reading speed. An additional challenge could also be rules that require linguistic judgments. For this reason, we replaced a rule that required a judgment about whether a word is a noun (in IS) with a rule that queries whether a word has a feminine grammatical gender (in RS).

We are placing the C3T in the public domain with CC-BY-SA 4.0 International license. In terms of further use and development, the following should be noted. First, there are currently a limited number of specific, appropriately balanced trials for each rule and with varying degrees of difficulty. To enable repeated use of the C3T (e.g., for use in a longitudinal study), a larger set of stimuli and rules would need to be prepared. Second, the current version of the C3T is prepared and available only in Slovenian. Some rules require an assessment of the linguistic properties of words (e.g., gender), which may not be present in other languages. Adaptation to another language would therefore require careful selection of verbal stimuli beyond their direct translation. Although this might play a negligible role in the performance of the task, it might be useful to match the frequency of the words used as well as their orthographic and semantic neighbourhoods in the different languages. Of course, the spoken words would also need to be recorded by a native speaker and prepared for monaural presentation.

## 5. Conclusions

In conclusion, to explore complex stable and flexible cognitive control across the lifespan, we developed a Cognitive Control Challenge Task (C3T). The results show that the C3T captures complex task set formation, task set activation, task set switching, and task set execution. Furthermore, examination of complex stable and flexible cognitive control across the lifespan has confirmed the expected U-shaped developmental curve. Specifically, C3T performance improves in childhood, peaks in emerging adulthood, and declines with further aging. In contrast, derived measures of complex task set formation and task set switching cost increase linearly across the lifespan. This result is best explained by the proposition that stable and flexible cognitive control are supported by separable cognitive systems.

## Data Availability Statement

The datasets presented in this study, the data analysis script and the C3T tasks used in this study can be found in Open Science Fundation online repository: https://osf.io/7tk9r/.

## Ethics Statement

The study involving human participant was reviewed and approved by the Ethics Committee of the Faculty of Arts, Ljubljana, Slovenia. Written informed consent to participate in this study was provided by each participant and, when required, co-signed by the participant's parent, legal guardian or next of kin.

## Author Contributions

VAP, ASO, and GR contributed to conception and design of the study. VAP and ASO prepared the materials for the task and organised and contributed to data collection. GR programmed the task. VAP and GR performed statistical analyses and wrote sections of the manuscript. VAP wrote the initial draft of the manuscript. All authors contributed to manuscript revision, read and approved the submitted version.

## Funding

This research was funded by grants of Slovenian Research Agency (Nos. J7-6829, P3-0338, and P5-0110) and Young Researcher Programme.

## Conflict of Interest

GR consults for and holds equity with Neumora Therapeutics. The remaining authors declare that the research was conducted in the absence of any commercial or financial relationships that could be construed as a potential conflict of interest.

## Publisher's Note

All claims expressed in this article are solely those of the authors and do not necessarily represent those of their affiliated organizations, or those of the publisher, the editors and the reviewers. Any product that may be evaluated in this article, or claim that may be made by its manufacturer, is not guaranteed or endorsed by the publisher.

## Footnotes

^1^The accuracy criterion was chosen to ensure that for both samples (IS and RS) the probability that the participant was guessing rather than performing the task was less than 10%.
