# Supplementary material for: Cognitive Control Challenge Task Across the Lifespan
Source: Front Psychol. 2022 Feb 9;12:789816. doi: 10.3389/fpsyg.2021.789816 (PMC8864288; doi:10.3389/fpsyg.2021.789816)
Supplement: Supplementary file 1 [file Data_Sheet_1.PDF]

Age distribution of excluded participants

Due to low accuracy, 26 and 14 participants were excluded from the initial and replication samples, respectively. To determine whether some age groups found the task more difficult than others, we examined the number of participants excluded due to low accuracy by age group for each of the samples and across the two samples. The results show that in the initial sample, a significantly higher proportion of participants in the late childhood and late adulthood age groups had to be excluded for poor performance than in the other age groups (see Table S1). In the replication sample, the proportion of excluded participants from the adolescent and emerging adulthood age groups was still significantly lower than in the other age groups, but the differences were much less pronounced.

Table S1. *The number and percentage of participants excluded due to low accuracy in each of the samples and in both samples. N - number of participants with complete C3T data, E - number of participants excluded due to low accuracy in the task, (%) percentage of participants excluded due to low accuracy in the task.*

		Initial sample			Replication sample			Together		
**Developmental stage**	**Age (years)**	**N**	**E**	**(%)**	**N**	**E**	**(%)**	**N**	**E**	**(%)**
Late Childhood	8–12	6	2	(33)	15	1	(7)	21	3	(14)
Adolescence	13–17	28	2	(7)	27	1	(4)	55	3	(5)
Emerging Adulthood	18–30	81	2	(2)	83	2	(2)	164	4	(2)
Young Adulthood	31–45	11	2	(18)	12	2	(17)	23	4	(17)
Middle Adulthood	46–64	24	5	(21)	28	3	(11)	52	8	(15)
Late Adulthood	65–85	31	13	(42)	31	5	(16)	62	18	(29)
Together		181	26	(14)	196	14	(7)	377	40	(11)

Cognitive status of older adults

For studies that include older adults, there is an increased likelihood that the sample will include individuals with neuropsychological and/or neurodegenerative disorders, which could significantly affect the results in this age group. There are a number of ways to ensure that the sample of older adults represents healthy aging individuals. Due to the specifics of participant recruitment and testing, we did not use clinical screening instruments such as the Mini-Mental State Exam (MMSE). Instead, to identify individuals with likely cognitive decline beyond the effects of healthy aging, we examined the pattern of individual scores on the cognitive tests used (digit and letter span tests, verbal fluency test, trail making test, Tower of London test, and operational span test), as well as the results of the Cognitive Failures Questionnaire (CFQ) and Prospective and Retrospective Memory Questionaire (PRMQ). Specifically, we checked whether any individual consistently exhibited decreased performance on a range of cognitive tests and self-report measures.

Because individuals in the individuals from initial (IS) and the replication sample (RS) completed the same tests-with the exception of Towers of London (IS) and Operational span (RS) -we combined all participants from the Late Adulthood group into the same sample for this analysis.

First, we looked for participants for which the results were outside of 1.5 × IQR (interquartile range) on any of the measures when compared to the group. No participant was identified as an outlier on any of the measures.

Next, we standardized the results within each measure and expressed each participant's performance or questionnaire score as a z-score relative to the entire sample. We identified any scores that differed by more than 2 SD from the group mean and reflected substantially worse performance than average (i.e., longer reaction times, lower accuracy, or higher scores on CFQ and PRMQ). We then checked whether any of the individuals with such scores showed a consistent pattern of decreased performance across multiple measures. We assumed that any participant with an underlying neuropsychological or neurodegenerative disorder should have notably poorer performance on a range of measures. We identified 11 (IS: 4, RS: 7) participants with at least one deviant z-score. No participant had consistently poor scores on multiple measures (see Figure S1). Based on these results, we are confident that the older adults' scores were not affected by underlying neuropsychological or neurodegenerative disorders.

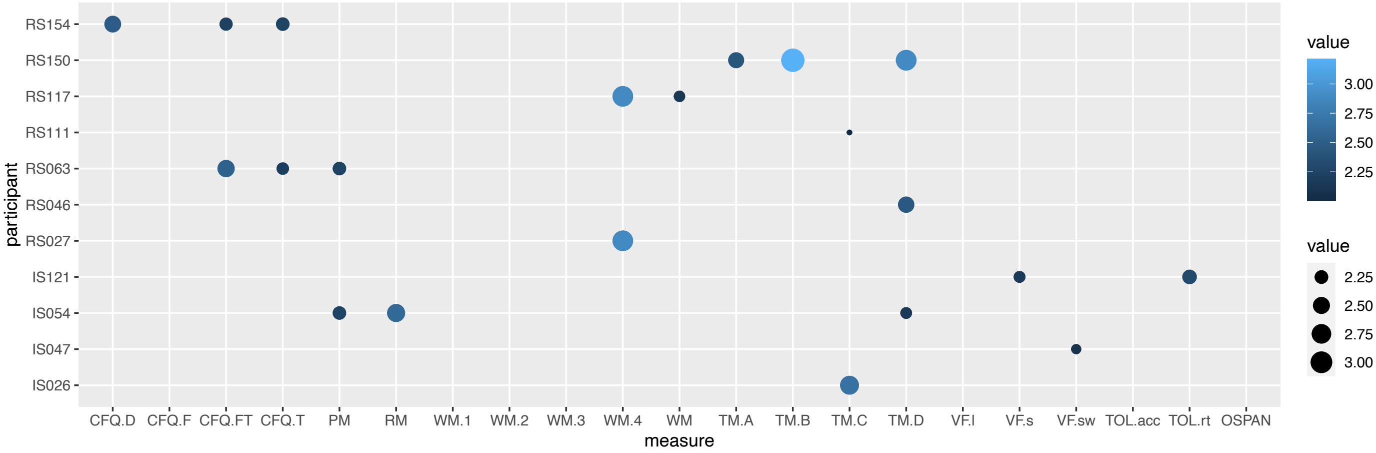

Figure S1. Z-scores on cognitive tests, CFQ and PRMQ that deviated more than 2 SD from the group mean, for Late Adulthood participants with at least one such z-score. Circle colors and sizes denote z-score values. CFQ.D, CFQ.F, CFQ.FT, and CFQ.T are distractibility, forgetefulness, false triggering and total scores on CFQ, respectively. PM and RM are prospective and retrospective memory scores on PRMQ, respectively. WM-1 thoroug WM-4 are individual digit and number span scores. WM is average span score. TM.A, TM.B, TM.C and TM.D are part A, part B, part C, and part B - part A difference scores on trail making test respectively. VF.l, VF.s, VF.sw, are lexical, semantic and category switching scores on verbal fluency test, respectively. TOL.acc and TOL.rt are accuracy and reaction time measures on the Tower of London test, respectively. OSPAN is the score on the operational span test.

Detailed results: Testing the C3T

The following tables provide detailed results of logistic and linear regressions used to test the properties of the C3T. Details on the methods are provided in the main manuscript.

Table S2

Results of the logistic regression model for the effects of task mode and trial order on accuracy for initial and replication samples. Trial order effect was modeled as a natural logarithm of the trial number, and task mode as a categorical variable.

Initial sample

Fixed effects	β	Standard Error	z-value	*p*-value	OR
intercept	-1.24	0.06	-20.46	< .001***	0.29
trial order	-0.15	0.03	-5.00	< .001***	0.87
task mode (flexible)	-0.19	0.07	-2.61	.009**	0.83
trial × mode interaction	0.15	0.04	3.80	< .001***	1.16

Random effects

Groups	Name	Variance	SD	Corr
Subject	intercept	0.17	0.42	
	trial order	0.01	0.08	1.00

Replication sample

Fixed effects	β	Standard Error	z-value	*p*-value	OR
intercept	-1.70	0.07	-23.60	< .001***	0.18
trial order	-0.19	0.03	-7.12	< .001***	0.83
task mode (flexible)	-0.14	0.07	-2.035	.042*	0.87
trial × mode interaction	0.13	0.04	3.798	< .001***	1.14

Random effects

Groups	Name	Variance	SD	Corr
Subject	intercept	0.48	0.69	
	trial order	0.02	0.13	0.35

Table S3

Results of comparisons of logistic regression models for the effects of task mode, trial order, and task mode × trial order on ***accuracy*** *for the* ***initial sample****. Trial order was modeled as the natural logarithm of the trial number and task mode was modeled as a categorical variable. For each effect, a full model is compared to a reduced model without the effect of interest. Full models for individual effects do not include the interaction term.*

Task mode × trial order interaction

model	npar	AIC	BIC	logLik	deviance	X^2	Df	*p*-value	R^2
reduced	6	14372	14418	-7180.2	14360				.004
full	7	14360	14413	-7172.9	14346	14.57	1	< .001***	.005

Trial order

model	npar	AIC	BIC	logLik	deviance	X^2	Df	*p*-value	R^2
reduced	5	14395	14433	-7192.6	14385				< .001
full	6	14372	14418	-7180.2	14360	24.74	1	< .001***	.004

Task mode

model	npar	AIC	BIC	logLik	deviance	X^2	Df	*p*-value	R^2
reduced	5	14374	14413	-7182.3	14364				.003
full	6	14372	14418	-7180.2	14360	4.15	1	.042*	.004

Table S3

*Summary of hierarchical linear modelling analyses for testing the change in preparation time and response time from first to second trial in stable and flexible mode. Degrees of freedom and p-values were established using Satterthwaite's method, CIs were estimated usinhg wild bootstrap procedure, f^2 was estimated using reduced models. Sig. codes are * p < .05, ** p < .01, *** p < .001. When CI inludes zero, the estimates were not considered significant.*

predictor	β	df	*t*-value	*p*-value	CI_{lo}	CI_{hi}	d	f2	sig.
**Preparation time**									
**Initial sample**									
trial	-2.831	467.0	-14.1	< .001	-3.293	-2.425	-0.772	0.139	***
mode	-4.800	466.9	-10.7	< .001	-5.714	-3.933	-1.310	0.008	***
trial × mode	2.891	467.0	10.1	< .001	2.401	3.436	0.789	0.078	***
**Replication sample**									
trial	-3.070	548.0	-16.3	< .001	-3.451	-2.703	-0.979	0.216	***
mode	-4.956	548.3	-11.7	< .001	-5.838	-4.103	-1.581	0.021	***
trial × mode	2.853	547.9	10.7	< .001	2.368	3.330	0.910	0.104	***
**Response time**									
**Initial sample**									
trial	-0.332	467.1	-2.31	0.021	-0.571	-0.093	-0.125	0.004	*
mode	-1.168	467.1	-3.63	< .001	-1.753	-0.524	-0.441	0.031	***
trial × mode	0.340	467.1	1.67	0.096	-0.060	0.668	0.128	0.002	
**Replication sample**									
trial	-0.436	547.7	-3.35	< .001	-0.655	-0.225	-0.152	0.006	***
mode	-1.508	547.8	-5.18	< .001	-2.086	-0.909	-0.525	0.027	***
trial × mode	0.561	547.7	3.05	0.002	0.191	0.896	0.195	0.005	**

Table S4

Results of comparisons of linear regression models for the effects of trial (first, second), task mode, and task mode × trial interaction on ***preparation time*** *for the* ***initial sample****. Task mode was modeled as a categorical variable. For each effect, a full model is compared to a reduced model without the effect of interest. Full models for individual effects do not include the interaction term.*

Task mode × trial interaction

model	npar	AIC	BIC	logLik	deviance	X^2	Df	*p*-value	R^2
reduced	5	3285.3	3307.5	-1637.6	3275.3				.128
full	6	3194.3	3220.9	-1591.2	3182.3	93.0	1	< .001	.191

Trial

model	npar	AIC	BIC	logLik	deviance	X^2	Df	*p*-value	R^2
reduced	4	3422.6	3440.4	-1707.3	3414.6				.006
full	5	3285.3	3307.5	-1637.6	3275.3	139.4	1	< .001	.128

Task mode

model	npar	AIC	BIC	logLik	deviance	X^2	Df	*p*-value	R^2
reduced	4	3292.0	3309.7	-1642.0	3284.0				.121
full	5	3285.3	3307.5	-1637.6	3275.3	8.7	1	0.003	.128

Table S5

Results of comparisons of linear regression models for the effects of trial (first, second), task mode, and task mode × trial interaction on ***preparation time*** *for the* ***replication sample****. Task mode was modeled as a categorical variable. For each effect, a full model is compared to a reduced model without the effect of interest. Full models for individual effects do not include the interaction term.*

Task mode × trial interaction

model	npar	AIC	BIC	logLik	deviance	X^2	Df	*p*-value	R^2
reduced	5	3761.3	3784.3	-1875.6	3751.3				.191
full	6	3659.6	3687.2	-1823.8	3647.6	103.6	1	< .001	.267

Trial

model	npar	AIC	BIC	logLik	deviance	X^2	Df	*p*-value	R^2
reduced	4	3941.8	3960.2	-1966.9	3933.8				.016
full	5	3761.3	3784.3	-1875.6	3751.3	182.5	1	< .001	.191

Task mode

model	npar	AIC	BIC	logLik	deviance	X^2	Df	*p*-value	R^2
reduced	4	3779.6	3798.0	-1885.8	3771.6				.174
full	5	3761.3	3784.3	-1875.6	3751.3	20.3	1	< .001	.191

Table S6

Results of comparisons of linear regression models for the effects of trial (first, second), task mode, and task mode × trial interaction on ***response time*** *for the* ***initial sample****. Task mode was modeled as a categorical variable. For each effect, a full model is compared to a reduced model without the effect of interest. Full models for individual effects do not include the interaction term.*

Task mode × trial interaction

model	npar	AIC	BIC	logLik	deviance	X^2	Df	*p*-value	R^2
reduced	5	2780.8	2803.0	-1385.4	2770.8				.034
full	6	2780.1	2806.7	-1384.0	2768.1	2.78	1	0.095	.036

Trial

model	npar	AIC	BIC	logLik	deviance	X^2	Df	*p*-value	R^2
reduced	4	2784.1	2801.9	-1388.1	2776.1				.030
full	5	2780.8	2803.0	-1385.4	2770.8	5.29	1	0.021	.034

Task mode

model	npar	AIC	BIC	logLik	deviance	X^2	Df	*p*-value	R^2
reduced	4	2818.8	2836.6	-1405.4	2810.8				.004
full	5	2780.8	2803.0	-1385.4	2770.8	40.0	1	0.003	.034

Table S7

Results of comparisons of linear regression models for the effects of trial (first, second), task mode, and task mode × trial interaction on ***response time*** *for the* ***replication sample****. Task mode was modeled as a categorical variable. For each effect, a full model is compared to a reduced model without the effect of interest. Full models for individual effects do not include the interaction term.*

Task mode × trial interaction

model	npar	AIC	BIC	logLik	deviance	X^2	Df	*p*-value	R^2
reduced	5	3291.2	3314.2	-1640.6	3281.2				.031
full	6	3284.0	3311.6	-1636.0	3272.0	9.23	1	0.00238	.036

Trial

model	npar	AIC	BIC	logLik	deviance	X^2	Df	*p*-value	R^2
reduced	4	3300.1	3318.4	-1646.0	3292.1				.026
full	5	3291.2	3314.2	-1640.6	3281.2	10.8	1	.001	.031

Task mode

model	npar	AIC	BIC	logLik	deviance	X^2	Df	*p*-value	R^2
reduced	4	3338.5	3356.8	-1665.2	3330.5				.005
full	5	3291.2	3314.2	-1640.6	3281.2	49.2	1	< .001	.031

Table S8

*Results of comparisons of linear regression models for the effects of task mode, trial order, and task mode × trial order on **preparation time** for the **initial sample**. Trial order was modeled as the natural logarithm of the trial number and task mode was modeled as a categorical variable. For each effect, a full model is compared to a reduced model without the effect of interest. Full models for individual effects do not include the interaction term.*

Task mode × trial order interaction

model	npar	AIC	BIC	logLik	deviance	X^2	Df	*p*-value	R^2
reduced	6	13025	13061	-6506.3	13013				.099
full	7	13027	13070	-6506.3	13013	0.04	1	.842	.099

Trial order

model	npar	AIC	BIC	logLik	deviance	X^2	Df	*p*-value	R^2
reduced	5	13088	13118	-6538.8	13078				.071
full	6	13025	13061	-6506.3	13013	64.9	1	< .001***	.099

Task mode

model	npar	AIC	BIC	logLik	deviance	X^2	Df	*p*-value	R^2
reduced	5	13803	13834	-6896.5	13793				.019
full	6	13025	13061	-6506.3	13013	780.4	1	< .001***	.099

Table S9

*Results of comparisons of linear regression models for the effects of task mode, trial order, and task mode × trial order on **preparation time** for the **replication sample**. Trial order was modeled as the natural logarithm of the trial number and task mode was modeled as a categorical variable. For each effect, a full model is compared to a reduced model without the effect of interest. Full models for individual effects do not include the interaction term.*

Task mode × trial order interaction

model	npar	AIC	BIC	logLik	deviance	X^2	Df	*p*-value	R^2
reduced	6	18485	18525	-9236.5	18473				.109
full	7	18466	18513	-9226.2	18452	20.68	1	< .001***	.111

Trial order

model	npar	AIC	BIC	logLik	deviance	X^2	Df	*p*-value	R^2
reduced	5	18628	18661	-9309.0	18618				.061
full	6	18485	18525	-9236.5	18473	145.02	1	< .001***	.109

Task mode

model	npar	AIC	BIC	logLik	deviance	X^2	Df	*p*-value	R^2
reduced	5	19286	19318	-9637.7	19276				.031
full	6	18485	18525	-9236.5	18473	802.39	1	< .001***	.109

Table S10

Results of comparisons of linear regression models for the effects of task mode, trial order, and task mode × trial order effect on ***response time*** *for the* ***initial sample****. Trial order effect was modeled as a natural logarithm of the trial number and task mode as a categorical variable. For each effect, a full model is compared to a reduced model without the effect of interest. Full models for individual effects do not include the interaction term.*

Task mode × trial order interaction

model	npar	AIC	BIC	logLik	deviance	X^2	Df	*p*-value	R^2
reduced	6	11940	11977	-5964.2	11928				.008
full	7	11936	11980	-5961.2	11922	5.88	1	.015*	.009

Trial order

model	npar	AIC	BIC	logLik	deviance	X^2	Df	*p*-value	R^2
reduced	5	11979	12010	-5984.7	11969				.002
full	6	11940	11977	-5964.2	11928	40.97	1	< .001***	.008

Task mode

model	npar	AIC	BIC	logLik	deviance	X^2	Df	*p*-value	R^2
reduced	5	11958	11988	-5973.8	11948				.006
full	6	11940	11977	-5964.2	11928	19.27	1	< .001***	.008

Table S11

Results of comparisons of linear regression models for the effects of task mode, trial order, and task mode × trial order effect on ***response time*** *for the* ***replication sample****. Trial order effect was modeled as a natural logarithm of the trial number and task mode as a categorical variable. For each effect, a full model is compared to a reduced model without the effect of interest. Full models for individual effects do not include the interaction term.*

Task mode × trial order interaction

model	npar	AIC	BIC	logLik	deviance	X^2	Df	*p*-value	R^2
reduced	6	17805	17844	-8896.4	17793				.012
full	7	17785	17831	-8885.3	17771	22.17	1	< .001***	.013

Trial order

model	npar	AIC	BIC	logLik	deviance	X^2	Df	*p*-value	R^2
reduced	5	17863	17896	-8926.4	17853				.002
full	6	17805	17844	-8896.4	17793	60.17	1	< .001***	.012

Task mode

model	npar	AIC	BIC	logLik	deviance	X^2	Df	*p*-value	R^2
reduced	5	17863	17896	-8926.7	17853				.011
full	6	17805	17844	-8896.4	17793	60.66	1	< .001***	.012

Table S12

Results of comparisons of linear regression models for the effects of task mode, trial order, and task mode × trial order on ***total time*** *for the* ***initial sample****. Trial order was modeled as the natural logarithm of the trial number and task mode was modeled as a categorical variable. For each effect, a full model is compared to a reduced model without the effect of interest. Full models for individual effects do not include the interaction term.*

Task mode × trial order interaction

model	npar	AIC	BIC	logLik	deviance	X^2	Df	*p*-value	R^2
reduced	6	15340	15377	-7664.1	15328				.036
full	7	15338	15381	-7661.8	15324	4.63	1	.031*	.036

Trial order

model	npar	AIC	BIC	logLik	deviance	X^2	Df	*p*-value	R^2
reduced	5	15409	15440	-7699.7	15399				.019
full	6	15340	15377	-7664.1	15328	71.22	1	< .001***	.036

Task mode

model	npar	AIC	BIC	logLik	deviance	X^2	Df	*p*-value	R^2
reduced	5	15681	15711	-7835.3	15671				.014
full	6	15340	15377	-7664.1	15328	342.43	1	< .001***	.036

Table S13

Results of comparisons of linear regression models for the effects of task mode, trial order, and task mode × trial order on ***total time*** *for the* ***replication sample****. Trial order was modeled as the natural logarithm of the trial number and task mode was modeled as a categorical variable. For each effect, a full model is compared to a reduced model without the effect of interest. Full models for individual effects do not include the interaction term.*

Task mode × trial order interaction

model	npar	AIC	BIC	logLik	deviance	X^2	Df	*p*-value	R^2
reduced	6	22872	22911	-11430	22860				.033
full	7	22872	22919	-11429	22858	1.17	1	.280	.033

Trial order

model	npar	AIC	BIC	logLik	deviance	X^2	Df	*p*-value	R^2
reduced	5	22993	23026	-11492	22983				.008
full	6	22872	22911	-11430	22860	123.69	1	< .001***	.033

Task mode

model	npar	AIC	BIC	logLik	deviance	X^2	Df	*p*-value	R^2
reduced	5	23116	23149	-11553	23106				.023
full	6	22872	22911	-11430	22860	246.44	1	< .001***	.033

Speed-accuracy trade-off

In cognitive tasks that require both fast and accurate responses, participants may attach different importance to accuracy and speed of response. This can occur both within an individual as well as across participants. Within an individual, a participant may decide to take more time to increase their accuracy on some trials and risk a higher probability of an incorrect response to respond faster on other trials. Similarly, some participants may value accuracy more at the cost of reduced speed of processing, while others may trade accuracy for increased speed of responses. The presence of such a speed-accuracy trade-off could present a confound when comparing task conditions or groups of participants. To ensure the validity of the results and to determine whether a speed-accuracy trade-off could be a potential problem when using C3T, we examined the potential presence of a speed-accuracy trade-off both participants and within individuals.

To estimate the speed-accuracy trade-off across participants, we computed a correlation between participants' error rates and reaction times, separately for the flexible and stable task modes. Since the error rates are not normally distributed, we calculated Spearman ρ as a measure of correlation. To estimate the speed-accuracy trade-off within participants, we converted reaction times on incorrect trials into z-scores standardised relative to the mean and standard deviation of correct trials. The average z across the incorrect trials was used as a measure of the participant- and mode-specific speed-accuracy trade-off SAT_w. Specifically, the following equation was used:

$$SAT_w = \frac{\sum_{i=1}^{N_e} \frac{rt_i - \bar{rt}_c}{sd_c}}{N_e}$$

where, N_e is the number of error trials, rt_i is the reaction time on the i incorrect trial, $\bar{rt}_c$ is the average reaction time on correct trials, and sd_c is the standard deviation of reaction times on correct trials. A negative SAT_w shows that errors primarily occured when participants were fast, indicating a possible trade-off between speed and accuracy trade-off. A positive SAT_w, on the other hand, shows that errors occured on trials that also took longer to process, which suggest that there was no trade-off between speed and accuracy.

Results showed that across participants, for all time measures, correlations with error rates were positive and ranged between [.08, .34] (IS) and [.12, .34] (RS) for both stable and flexible task performance (see Table S14 and Figure S2 for details), indicating that preparation time, response time, total time, and accuracy reflect task difficulty without a speed-accuracy trade-off. The examination of trade-off between speed and accuracy within subjects showed that the mean z-scores across participants and time measures were positive with a single exception, ranging from [-0.067, 0.372] and [0.014, 0.307] (IS and RS, respectively; see Table S15 and Figure S3 for details). The 95% of participant's z-scores across time measures and task modes ranged between [-0.966, 1.879] and [-0.756, 1.467] (IS and RS, respectively). Taken together, these results indicate a lack of within-subject speed-accuracy trade-off.

Table S14

Speed-accuracy trade-off across participants for each time measure. Spearman ρ, a measure of correlation between participants' error rates and reaction times, for flexible and stable mode separately.

Sample	Task mode	Time	Spearman ρ	*p*-value
Initial sample	Stable	Response	0.32	< .001***
		Preparation	0.32	< .001***
		Total	0.34	< .001***
	Flexible	Response	0.28	< .001***
		Preparation	0.09	0.254
		Total	0.18	.022*
Replication sample	Stable	Response	0.28	< .001***
		Preparation	0.38	< .001***
		Total	0.34	< .001***
	Flexible	Response	0.31	< .001***
		Preparation	0.12	0.099
		Total	0.25	< .001***

Table S15

Speed-accuracy trade off within participants for each time measure. Reaction times on incorrect trials were converted to z-scores standardized relative to mean and standard deviation of correct trials. The average z-score across the incorrect trials was used as a measure of participant's mode specific speed-accuracy trade-off.

Sample	Task mode	Time	z-score	*sd*
Initial sample	Stable	Response	0.17	0.59
		Preparation	0.37	0.70
		Total	0.37	0.68
	Flexible	Response	-0.07	0.50
		Preparation	0.28	0.52
		Total	0.11	0.54
Replication sample	Stable	Response	0.10	0.46
		Preparation	0.28	0.54
		Total	0.28	0.52
	Flexible	Response	0.01	0.39
		Preparation	0.30	0.54
		Total	0.25	0.46

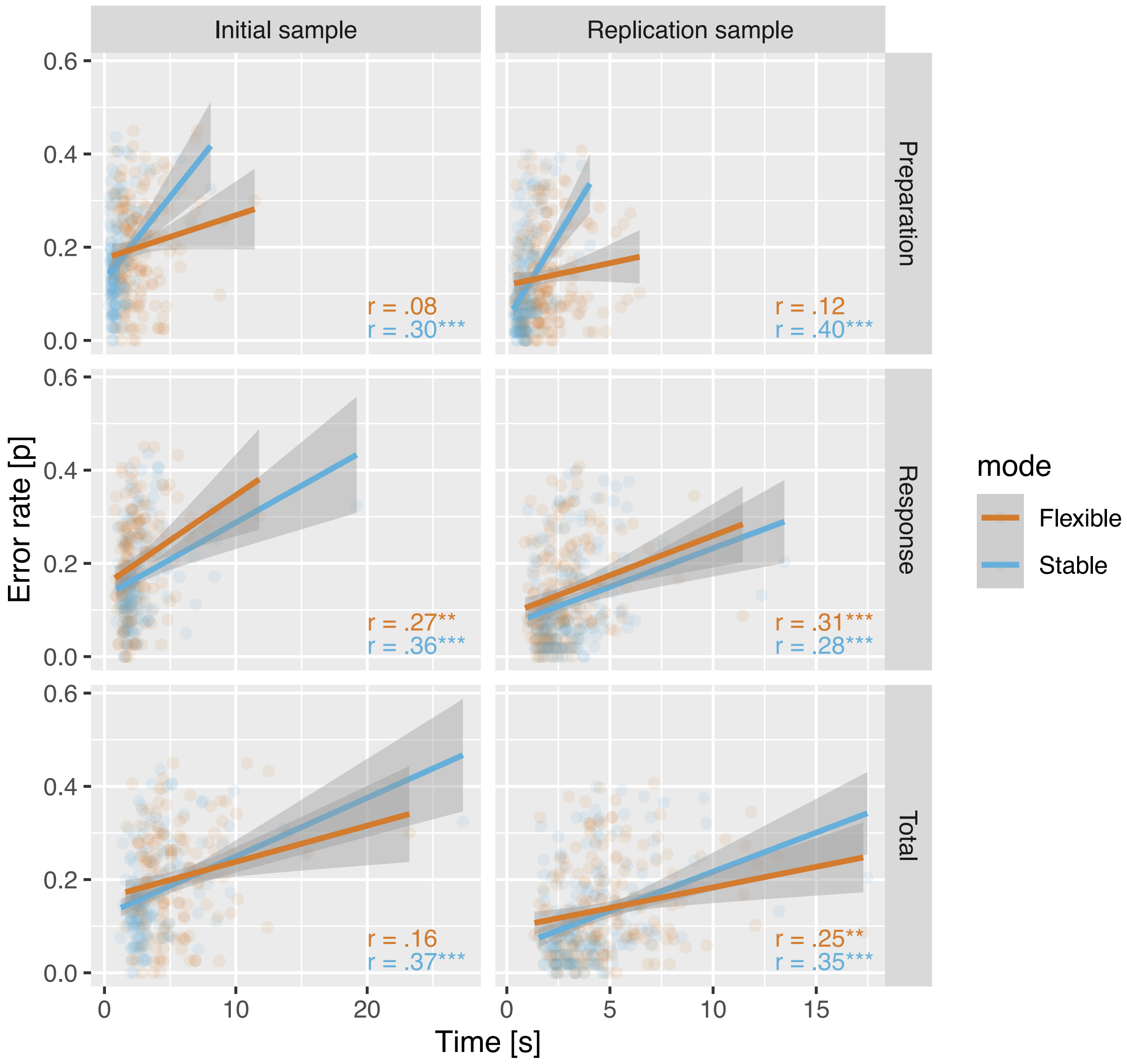

Figure S2. Speed-accuracy trade-off acrross participants for flexible and stable task modes in the initial and replication samples for preparation, response and total times. The colored dots are individual participants' results, the colored lines show the prediction of the mean based on linear regression, the gray area denotes the standard error of the prediction. Spearman ρ as a measure of correlation between participants' error rates and reaction times is shown embedded. Statistical significance of the correlations is designated using * $p < .05$, ** $p < .01$, *** $p < .001$.

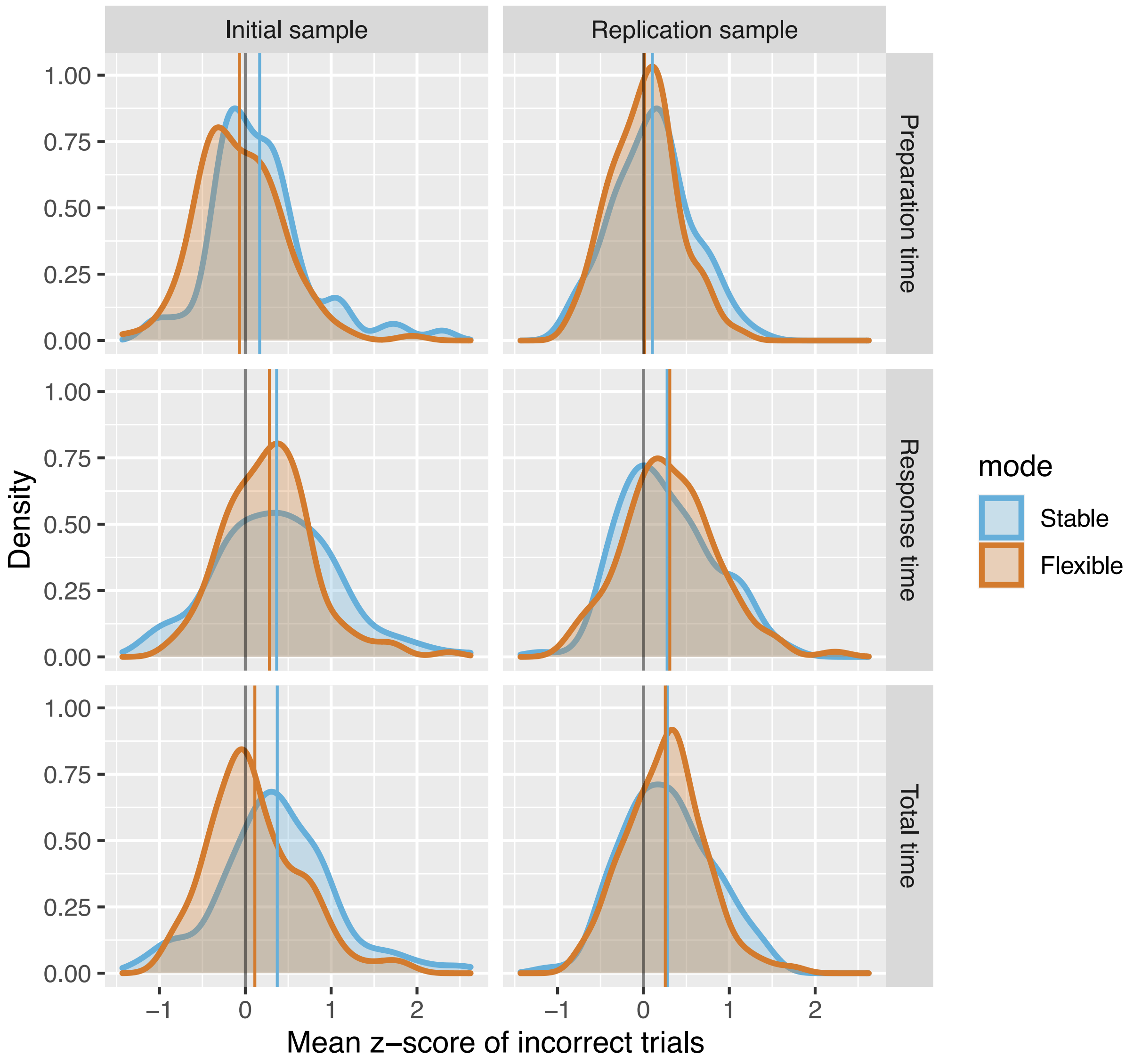

Figure S3. Distribution of within participant speed-accuracy trade-off estimates for flexible and stable task modes in the initial and replication samples for preparation, response and total times. The colored areas show the distribution density of mean z-scores of times on incorrect trials standardized on correct trials. The colored vertical lines show the distribution mean.

Preparation-response trade-off

Similarly to speed-accuracy trade-off, participants could trade-off between time to refresh, reactivate or switch between task-sets with the speed of providing a response. Participants could on some trials decide to progress from rule presentation to stimulus presentation before they have finished preparing their task set and could use some of the reponse time to finalize task set preparation before starting to process the stimuli already shown. In such cases preparation would bleed into reponse period leading to underestimation of preparation time and overestimation of response time. If the presence or extent of such preparation-response trade-off differs systematically across conditions within individuals or across individual, this could present a counfound for the obtained results.

To investigate the presence of such trade-off in our data and the potential for the trade-off in C3T we have examined the trade-off between preparation and response at two levels. To determine the trade-off between preparation and response across participants, we calculated a correlation between participants' average preparation and response times for each task mode. To examine the trade-off between preparation and response within participants, we calculated the correlation between preparation and response times across all trials for each participant and task mode (stable and flexible). A negative correlation would indicate a possible trade-off between the time spent preparing or refreshing a task rule and the time spent applying that rule.

Analysis across participants revealed high positive correlations between preparation and response times in both stable (IS: $\rho = .61$, RS: $\rho = .69$) and flexible (IS: $\rho = .64$, RS: $\rho = .63$) task modes (see Figure S4 for details), indicating a lack of preparation-response trade off. In other words, the participant who took longer to prepare also took longer to respond to the trial. The within-subjects analysis also indicated positive average correlations between preparation and response times for both the stable (IS: $m_\rho = .17$, RS: $m_\rho = .12$) and flexible (IS: $m_\rho = .03$, RS: $m_\rho = .07$) task modes. The 95% od participant's correlations across task modes ranged between [-.37, .56] and [-.24, .42] (IS and RS, respectively; see Table S16 and Figure S5 for details). Taken together, these results indicate a lack of preparation-response trade-off in the majority of participants, which in some individuals might reach a weak relationship.

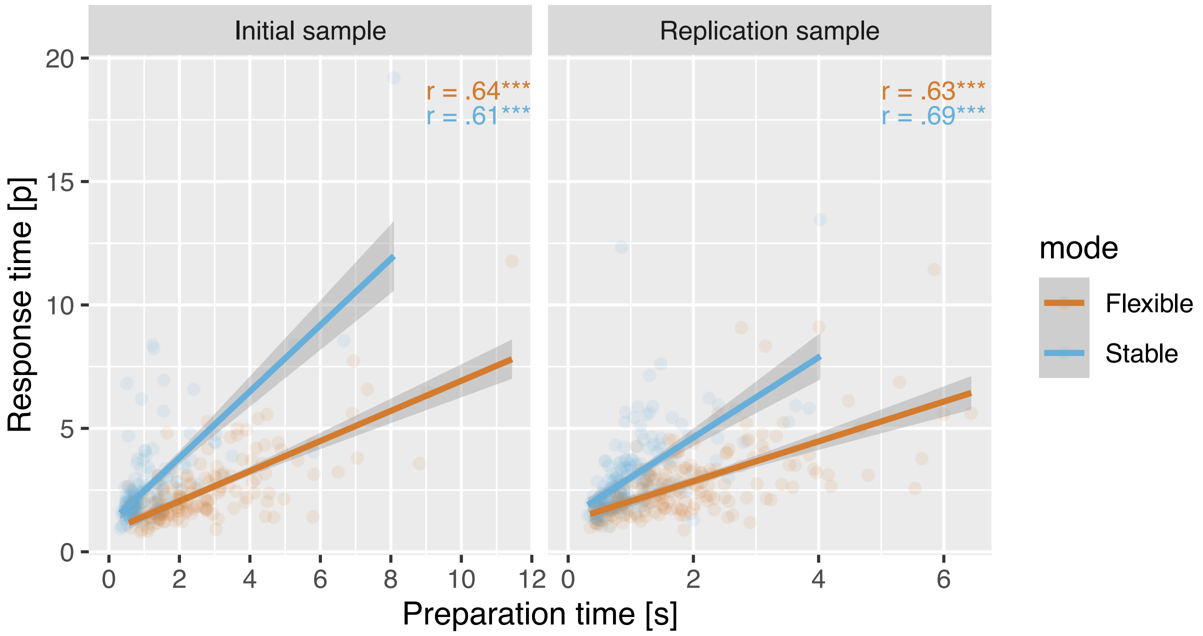

Figure S4. Response-preparation trade-off across participants for flexible and stable task modes in the initial and replication samples. The colored dots are individual participants' results, the colored lines show the prediction of the mean based on linear regression, the gray area denotes the standard error of the prediction. Spearman ρ as a measure of correlation between participants' preparation and response times is shown embedded. Statistical significance of the correlations is designated using * p < .05, ** p < .01, *** p < .001.

Table S16

Preparation-response time trade off within participants. Spearman ρ correlation coefficients between preparation and response times across all trials for each participant and task mode were calculated and transformed to Fisher z values to compute mean correlation.

Sample	Task mode	mean ρ	mean Fz	sd(Fz)
Initial sample	Stable	0.17	0.18	0.23
	Flexible	0.03	0.03	0.22
Replication sample	Stable	0.12	0.12	0.18
	Flexible	0.07	0.07	0.18

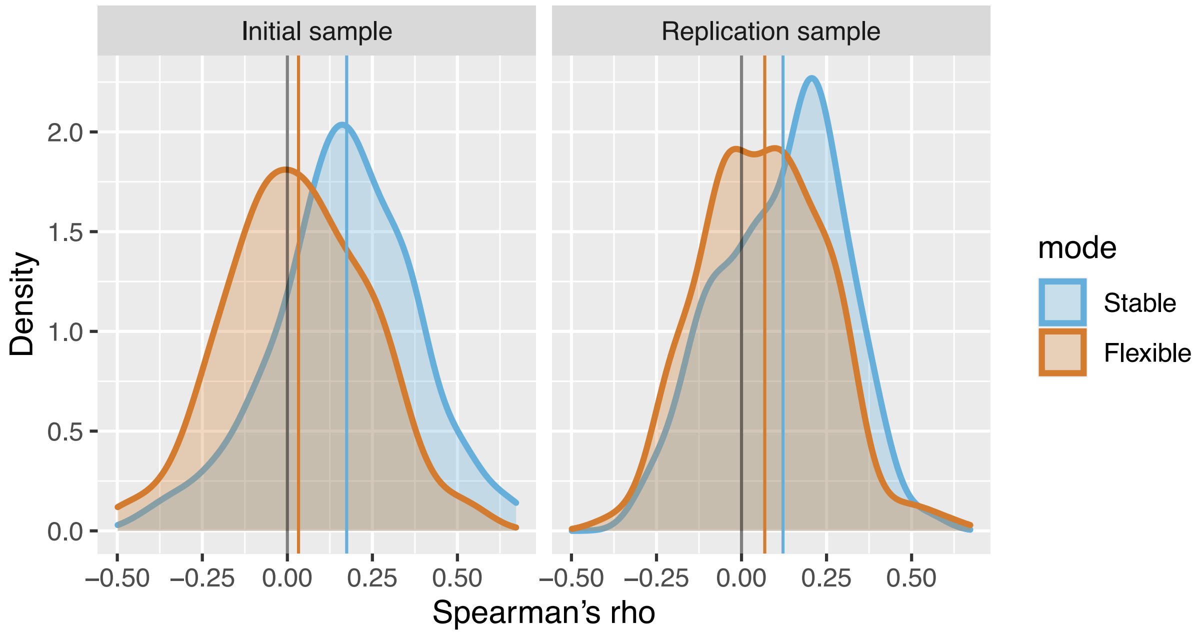

Figure S5. Distribution of within participant preparation-response trade-off estimates for flexible and stable task modes in the initial and replication samples. The colored areas show the distribution density of Spearman's ρ correlation coefficients between preparation and response times computed for each individual. The colored vertical lines show the distribution mean.

Detailed results: Changes across the lifespan

The following tables provide detailed results of logistic and linear regressions used to investigate the changes in C3T results across the lifespan. Details on the methods are provided in the main manuscript.

Table S17

Results of the logistic regression model for the effects of age and task mode on accuracy for the initial and replication samples. Age was modeled as a second polynomial of the logarithm of years of age and task mode as a categorical variable.

Initial sample

Fixed effects	β	Standard error	z value	Pr(>lzl)
intercept	-1.48	0.04	-33.87	< .001***
age (linear)	16.13	5.21	3.097	.002**
age (quadratic)	26.88	5.21	5.161	< .001***
task mode (flexible)	0.06	0.03	1.96	.049*
age (linear) × task mode	0.72	3.36	0.22	.829
age (quadratic) × task mode	0.68	3.36	0.20	.840

Random effects

groups	Name	Variance	SD
subject	intercept	0.22	0.47

Replication sample

Fixed effects	β	Standard error	z value	Pr(>lzl)
intercept	-2.05	0.06	-35.56	< .001***
age (linear)	33.79	8.68	3.89	< .001***
age (quadratic)	39.60	8.69	4.56	< .001***
task mode (flexible)	0.11	0.03	3.71	< .001***
age (linear) × task mode	-1.34	3.91	-0.34	.731
age (quadratic) × task mode	-2.01	3.92	-0.51	.609

Random effects

Groups	Name	Variance	SD
subject	intercept	0.52	0.72

Table S18
Results of comparisons of logistic regression models for the effects of task mode, age, and *task mode ×* age *on* ***accuracy*** *for the* ***initial sample****. Age was modeled as a second polynomial of the logarithm of years of age and task mode as a categorical variable. For each effect, a full model is compared to a reduced model without the effect of interest. Full models for individual effects do not include the interaction term.*

Task mode × age interaction

model	npar	AIC	BIC	logLik	deviance	X^2	Df	*p*-value	R^2
reduced	5	14362	14401	-7176.3	14352				.019
full	7	14366	14420	-7176.2	14352	0.09	2	.957	.019

Age

model	npar	AIC	BIC	logLik	deviance	X^2	Df	*p*-value	R^2
reduced	3	14392	14415	-7193.0	14386				< .001
full	5	14362	14401	-7176.3	14352	33.40	2	< .001***	.019

Task mode

model	npar	AIC	BIC	logLik	deviance	X^2	Df	*p*-value	R^2
reduced	4	14365	14395	-7178.3	14357				.018
full	5	14362	14401	-7176.3	14352	4.12	1	.043*	.019

Table S19
Results of comparisons of logistic regression models for the effects of task mode, age, and *task mode ×* age *on* ***accuracy*** *for the* ***replication sample****. Age was modeled as a second polynomial of the logarithm of years of age and task mode as a categorical variable. For each effect, a full model is compared to a reduced model without the effect of interest. Full models for individual effects do not include the interaction term.*

Task mode × age interaction

model	npar	AIC	BIC	logLik	deviance	X^2	Df	*p*-value	R^2
reduced	5	17390	17431	-8690.3	17380				.031
full	7	17394	17451	-8690.1	17380	0.38	2	.828	.031

Age

model	npar	AIC	BIC	logLik	deviance	X^2	Df	*p*-value	R^2
reduced	3	17421	17445	-8707.4	17415				.001
full	5	17390	17431	-8690.3	17380	34.21	2	< .001***	.031

Task mode

model	npar	AIC	BIC	logLik	deviance	X^2	Df	*p*-value	R^2
reduced	4	17402	17434	-8697.0	17394				.029
full	5	17390	17431	-8690.3	17380	13.52	1	< .001***	.031

Table S20

Results of the robust linear regression model for the effect of age on STI for the initial and replication samples. Age was modeled as a second polynomial of the logarithm of years of age.

Initial sample

predictors	*β*	SE	*t*-value	DF	*p*-value
Intercept	3.28	0.31	10.71	153	< .001***
age (linear)	10.44	3.58	2.92	153	.004**
age (quadratic)	-0.34	4.47	-0.08	153	.939

Replication sample

predictors	*β*	SE	*t*-value	DF	*p*-value
Intercept	4.08	0.29	13.85	177	< .001***
age (linear)	8.82	3.95	2.23	177	.027*
age (quadratic)	0.055	3.77	0.01	177	.989

Table S21

Results of the robust linear regression model for the effect of age on time-based SCI_t for the initial and replication samples. Age was modeled as a second polynomial of the logarithm of years of age.

Initial sample

predictors	*β*	SE	*t*-value	DF	*p*-value
Intercept	1.42	0.10	13.78	153	< .001***
age (linear)	2.42	1.53	1.58	153	.117
age (quadratic)	0.45	1.31	0.34	153	.732

Replication sample

predictors	*β*	SE	*t*-value	DF	*p*-value
Intercept	0.86	0.06	13.32	177	< .001***
age (linear)	2.63	1.06	2.48	177	.014*
age (quadratic)	0.12	0.93	0.13	177	.895

Table S22

Results of the robust linear regression model for the effect of age on error-based SCI_e for the initial and replication samples. Age was modeled as a second polynomial of the logarithm of years of age.

Initial sample

predictors	*β*	SE	*t*-value	DF	*p*-value
Intercept	0.03	0.03	4.41	153	< .001***
age (linear)	0.06	0.06	0.72	153	.475
age (quadratic)	0.06	0.06	0.73	153	.466

Replication sample

predictors	*β*	SE	*t*-value	DF	*p*-value
Intercept	0.02	0.01	3.91	177	< .001***
age (linear)	0.01	0.09	0.03	177	.976
age (quadratic)	0.05	0.12	0.43	177	.666

Numerical models of the possible causes of differences between flexible and stable task modes performance

To gain insight into the possible causes of the observed differences in the duration of preparation times between flexible and stable task modes, we simulated a set of different causes reflected in the (i) a constant increase in time in the flexible task mode, (ii) a relative increase in preparation time in the flexible task mode, (iii) an earlier or later development (i.e., peak performance) of flexible cognitive control compared to stable cognitive control. The simulated changes were applied to an approximation of preparation times across the lifespan. The results are compiled in Figure S6. See Methods in the main manuscript and the simulation code at OSF repository for details.

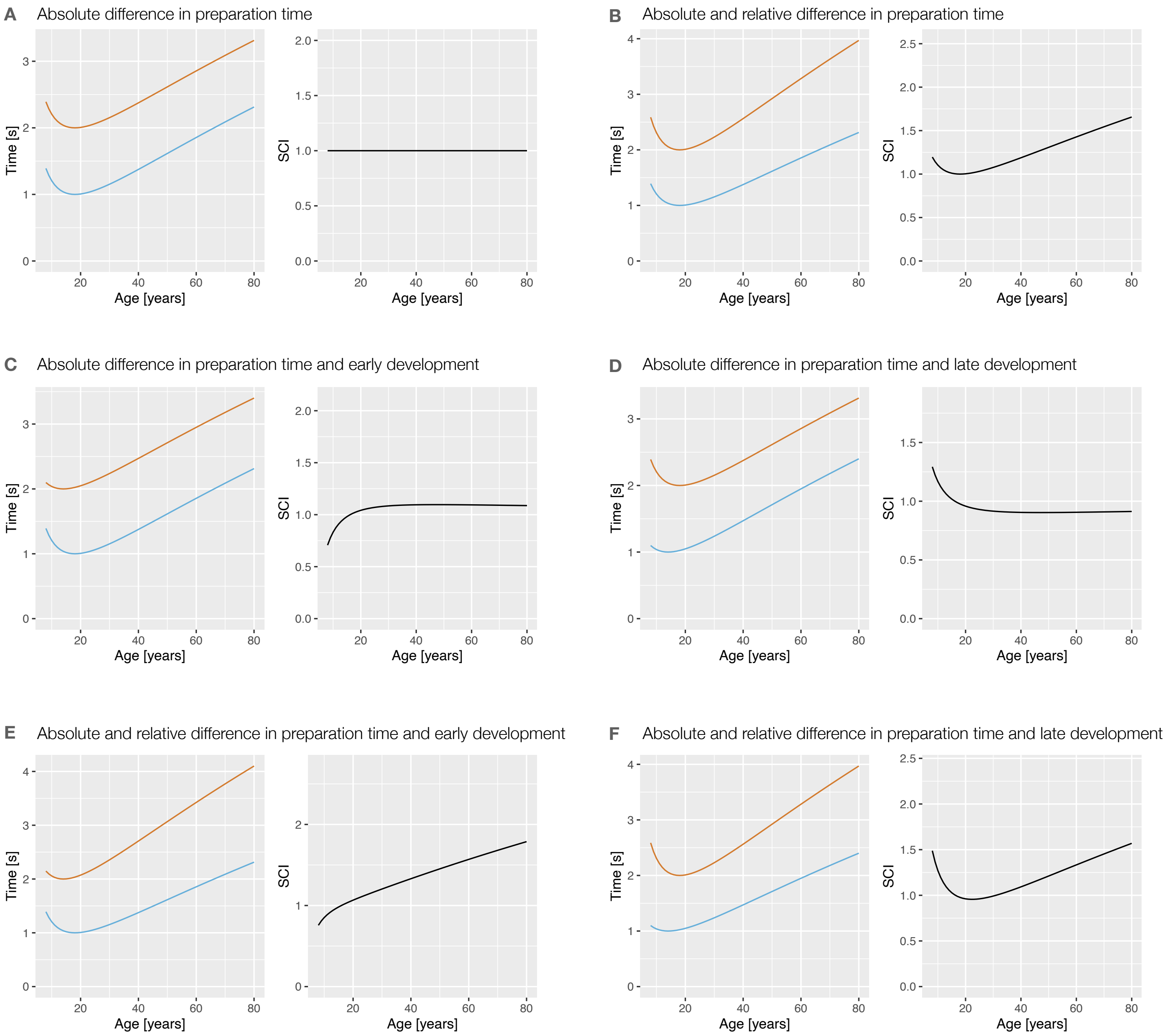

Figure S6. Results of simulated changes in preparation times in flexible (orange line) compared to stable (blue line) task mode. **A** addition of a fixed amount of time. **B** addition of a variable amount of time proportional to preparation time in stable task mode. **C** addition of a fixed amount of time and a shift of peak performance to earlier development. **D** addition of a fixed amount of time and a shift of peak performance to later development. **E** addition of variable amount of time proportional to preparation time in stable task mode and a shift of peak performance to earlier development. **F** addition of variable amonut of time proportional to preparation time in stable task mode and a shift of peak performance to later development.

Standard tests of cognitive control

To gain better understanding of the C3T performance, participants completed a number of standard tests that relate to cognitive control. A detailed description of each test follows here.

Trail making test
The Trail making test (TM) is one of the most popular neuropsychological tests and is included in most cognitive function test batteries. The version of the TM used in this study is publicly available (Reitan and Wolfson, 1985). It provides information on visual search, scanning, processing speed, mental flexibility, and executive functions. In the TM_A, a person must use a pencil to connect 25 circled numbers distributed on a sheet of paper. In the TM_B the person must alternate between numbers and letters (e.g., 1, A, 2, B, 3, C, etc.) when connecting the circles. TM_C was added to test psychomotor speed. In this part of the test, participants have to connect the circles as indicated by already present lines. The time taken to complete each part of the task is recorded and a difference between TM_A and TM_B ($TM_D = TM_B - TM_A$) is taken as a measure of task switching.

Digits and letter span test of working memory
The test consisted of 4 parts, with each part following the same procedure: A list of items was given, which the participants had to repeat according to a certain rule. The number of items started at three and was increased by one each time a participant repeated them successfully. In case of a mistake, the participant was given another chance to complete the task with a different set of items under the same load. When a participant failed to successfully complete the task with a given load after two consecutive attempts, the trials were terminated and the largest number of successfully retrieved items was recorded as the working memory span. The four parts were: Forward digit span task, in which the participant was read a list of random numbers and had to repeat them in the same order; Backward digit span task, in which the participant was asked to recall the numbers in reverse order; Alphabetical letter span, in which a sequence of letters was read and the participant had to repeat them in alphabetical order; Even-odd digit span, in which the participant was presented with a random sequence of digits that they had to repeat according to the position in which they appeared in the sequence; all digits in the even positions in the sequence had to be repeated first, followed by all the digits in the odd positions in the sequence. The spans for each of the four parts were recorded, and the average across all four parts was used as an estimate of verbal working memory.

Towers of London (computerized version)
The Tower of London test is a well-known test of executive function, with particular emphasis on planning (Shallice, 1982). We developed a computerised version consisting of 3 examples and 22 test situations. Participants were shown two images simultaneously. Each image showed a unique arrangement of a red, a blue, and a green ball positioned on three pegs, with the first peg able to hold three balls, the second able to hold two balls, and the last able to hold only one ball. Participants were asked to indicate the total number of moves required to make the arrangement of balls in the second image identical to that in the first image. A move is considered to be a change in the position of a single ball. In 5 situations the minimum number of moves required was 1, in 4 situations the minimum number of moves required was 2, in 5 situations the minimum number of moves required was 3, in 5 situations the minimum number of moves required was 4, in 1 situation the minimum number of moves required was 5, in 2 situations the minimum number of moves required was 6, in 8 situations there was more than one optimal sequence of moves to achieve the desired arrangement of the balls. The reaction time and accuracy of completion of each trial were recorded. The proportion of correct responses and the median reaction time for the correct responses were used in the analyses..

Operational span
An automated computerized version of the Operational Span (Ospan) task was constructed based on the original test by Unsworth, Heitz, Schrock, and Engle. (2005). The task was designed to measure complex working memory. This version of Ospan allowed the participant to perform the task independently of the experimenter. The task consisted of 15 trials. On each trial, participants were shown a sequence of mathematical equations for which they had to indicate whether they were correct or not, followed by a random letter. At the end of the sequence, participants had to type the sequence of letters shown in the correct order. The length of the trial sequences varied between 3 and 7 items. Three trials of each length were presented pseudorandomly. The task took approximately 15-20 minutes to complete. The sum of the lengths of the correctly retrieved sequences was taken as the final score. The task was considered valid if 75% of the equations were correctly evaluated.

Verbal fluency
In the Verbal Fluency task, participants had to produce as many words as possible from a category in a given time (60 seconds). Three lexical variants (words beginning with the letters S, I and T), two variants for semantic categories (animals, male names) and one variant for a semantic category switching (fruits and furniture) were completed. The average of the number of words generated in the three lexical variants was used as an estimate of lexical verbal fluency, the average of the two semantic category variants was used as an estimate of semantic verbal fluency, and the number of words reported in the semantic category switching as an estimate of verbal fluency.

References

Reitan, R. and Wolfson, D. (1985). *The Halstead-Reitan Neuropsychological Test Battery: Therapy and Clinical Interpretation*. Tucson, AZ: Neuropsychological Press.

Shallice, T. (1982). Specific Impairments of Planning. *Philosophical Transactions of the Royal Society B: Biological Sciences* 298, 199–209. doi:10/cgxv89

Unsworth, N., Heitz, R. P., Schrock, J. C., and Engle, R. W. (2005). An automated version of the operation span task. Behavior Research Methods 37, 498–505. doi:10/cgkprg

Lifespan changes on standard measures of cognitive control

Whereas the analysis of the changes on standard measures of cognitive control across the lifespan was not the aim of this study, a review of their trajectories can be helpful in better understanding the change in C3T measures across the lifespan. This section provides the plots of changes on cognitive control measures across the lifespan in which task performance was modeled as a second polynomial of the natural logarithm of age using robust linear regression, in the same way as C3T measures in the main manuscript.

Working memory span

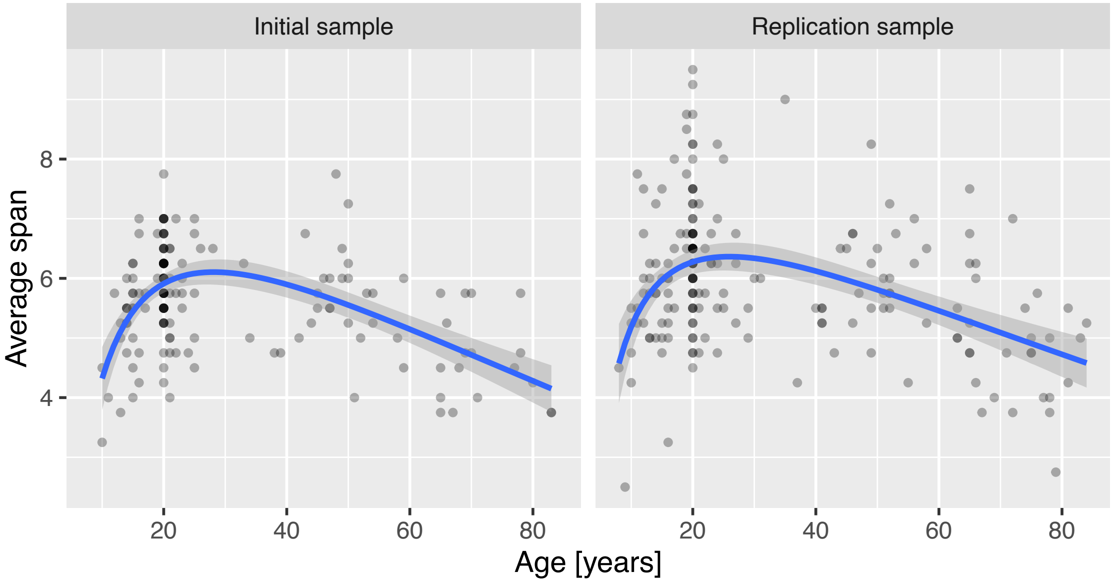

Figure S7. Average span achieved on working memory span tasks across the lifespan. The gray dots are individual participants' results, the blue line shows the prediction of robust linear regression, the gray area denotes the standard error of the prediction.

Trail making test

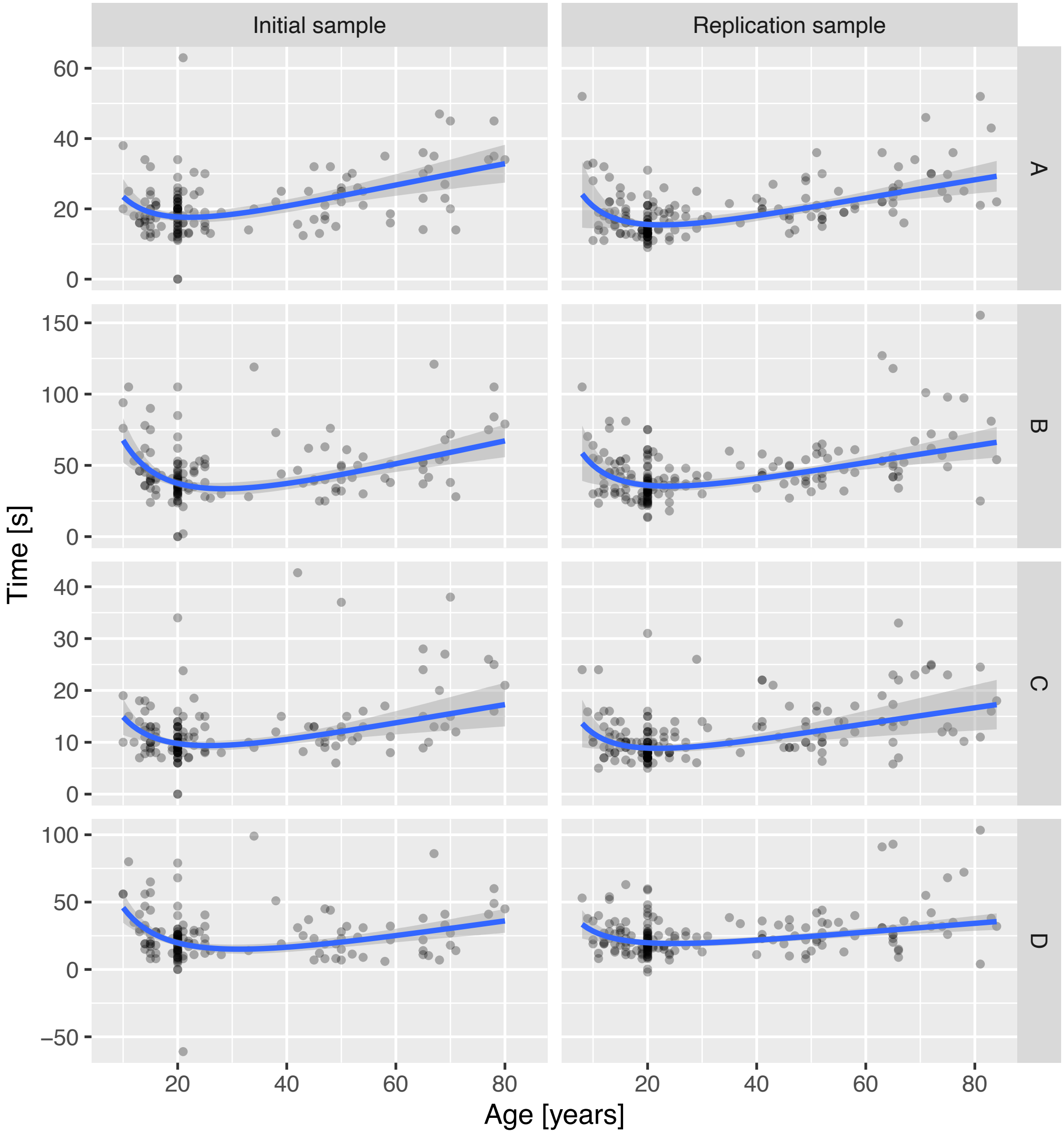

Figure S7. The times in seconds to complete the three parts of the Trail maiking test (A, B, and C), and the difference in times needed to complete parts A and B (D) across the lifespan. The gray dots are individual participants' results, the blue line shows the prediction of robust linear regression, the gray area denotes the standard error of the prediction.

Verbal fluency

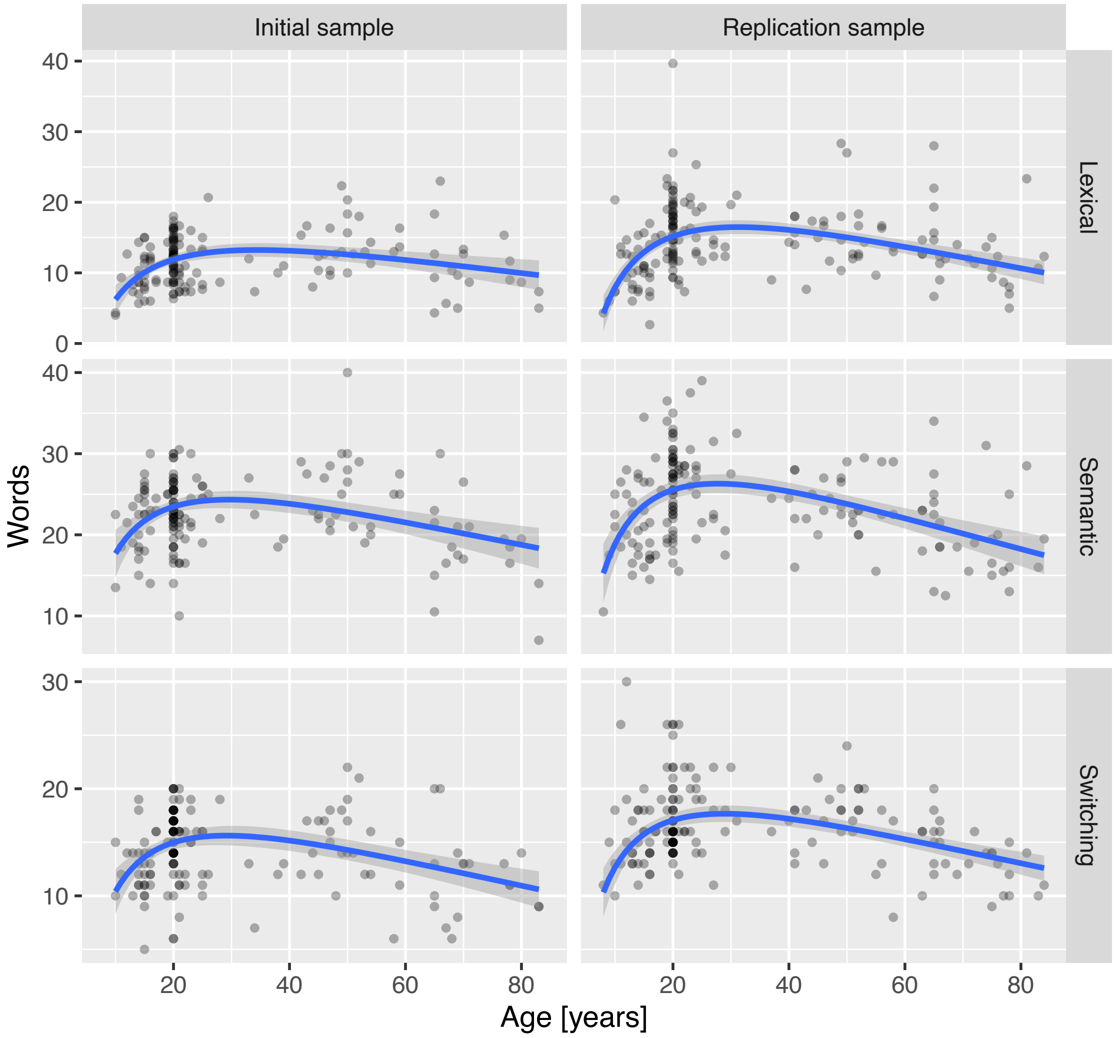

Figure S9. The average number of words prouced in the three parts (lexical, semantic, and category switching) of the verbal fluency task across the lifespan. The gray dots are individual participants' results, the blue line shows the prediction of robust linear regression, the gray area denotes the standard error of the prediction.

Tower of London

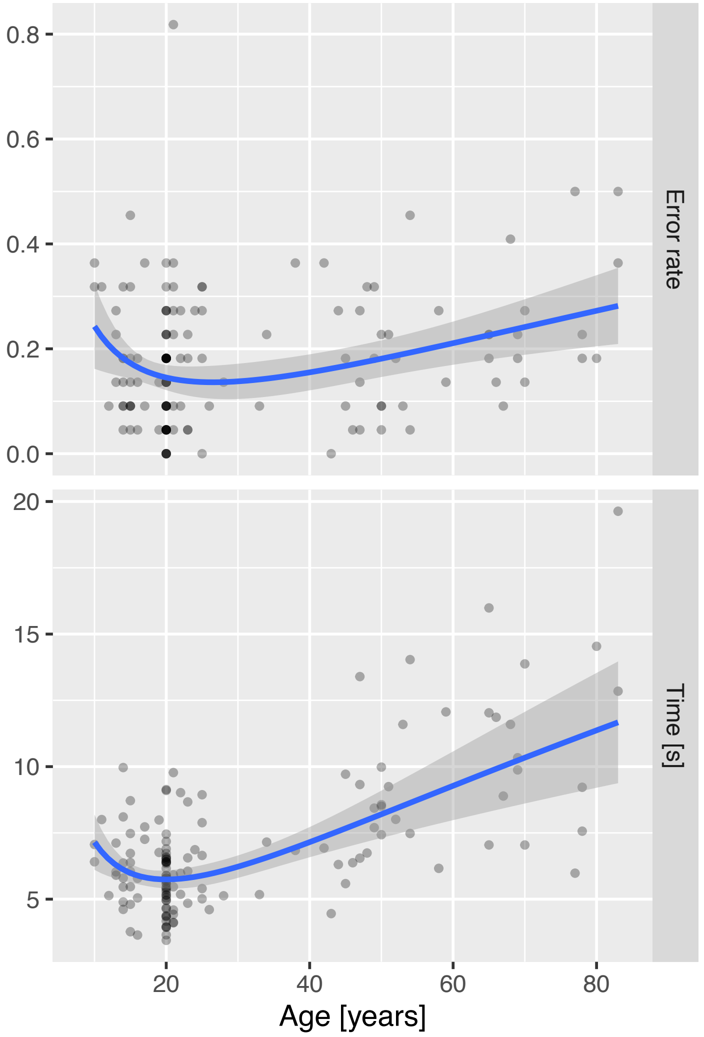

Figure S10. The error rate and the avarage time in seconds to complete each trial of the Tower of London task across the lifespan. The gray dots are individual participants' results, the blue line shows the prediction of robust linear regression, the gray area denotes the standard error of the prediction.

Operational span

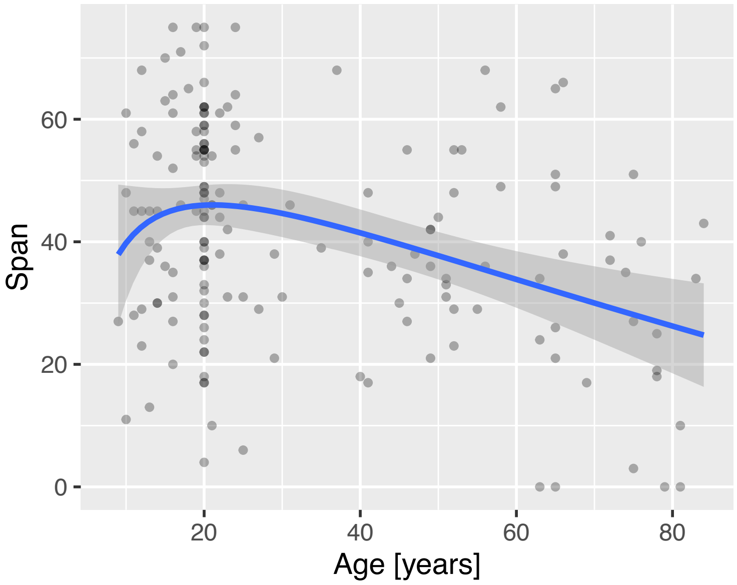

Figure S11. The total operational span across the lifespan. The gray dots are individual participants' results, the blue line shows the prediction of robust linear regression, the gray area denotes the standard error of the prediction.

Stimuli attributions

The following tables provide attribution information for the stimuli used in the C3T.

Table S23
List of C3T visual stimuli and licenses

Picture	License	Source
pineapple	FreeImages.com Content License	http://www.freeimages.com/photo/pineapple-1329154
car	creative commons attribution	https://www.flickr.com/photos/pictures-of-money/16678608434/in/photolist-rpQedo-qGCfWH-fFCTuo-fMffcZ-ojPHTP-acRB8K-ojanfE-aiFqpH-afaxQE-8iPncb-869mTF-bgSyJM-5MuNPP-9qyo4Z-eECW8k-to1BGA-qke4ku-c3uKu3-nNPj6R-mxv6oz-e4BbCE-4GRZdr-akzWQA-z1kFJt-nRnQPE-77P7Wu-AgMDYA-88pRcG-yNr7cP-afkexC-8QNw5d-aiFqpt-7FkL8x-7yUB4F-mxuBbz-6ftFKE-5i7hkC-8G4eNh-b9bK12-bqKx62-y3vwrE-8xyopP-9ANWUq-adnH2i-94wva4-6Wuhiv-9MQ2bh-wYBPFP-rvjjqp-7FkL8D
cushion	creative commons attribution	https://www.flickr.com/photos/52372445@N05/4828329493/in/photolist-8mEs9e-8mEs1T-8mErRk-4AcmDf-iEooD3-iEmFB6-6rhrZw-bWYxxE-3gj8V-5DjoPh-iEmEYH-iEqQS3-eK8rrg-7boK4H-yVaQWE-p4ZFGj-7BNh52-yVjGMe-4ApAev-4AtSG3-4BurCB-dVQeX4-6rhqvJ-3biZjj-bWYwhE-6rdgei-8JjEV4-8diyQu-rdfCX4-58jYHk-7bsxK1-6rhrK7-8BSDCJ-8z8xVe-f9PN1e-5G85WN-eKjRfY-mZGsFD-eKjRAW-febb2e-zzFMUp-88nS1n-mZGnPT-4BurDR-eKjRS7-eKjTzW-dVPkqx-eKjJ4w-eXNyps-mZGoDk
Bled	creative commons attribution	Mirjana Todorović
candy	creative commons attribution	https://www.flickr.com/photos/lucorreia/4151990768/in/photolist-9rE7h4-51A3xz-bbrCu2-pgGUe5-jcWGyc-bxmpvA-9sXmGo-6dpWzx-7fadd1-kWvCzJ-4jqY64-5FuBg4-eb2Khm-4AXDak-4rbjUC-6tBrQV-4p2hBi-aTqWen-8U2DtL-5mZNEp-6moTWV-Aw4myC-7PaUwx-7jU3cJ-btkdvi-5wAUjW-dE1KbU-jxvJfC-bDjYSg-7ywmeA-7Lbphz-5qucut-416Rbp-aaB5kp-4n3bBw-4naafQ-7dAWV-55WW9K-k7Gp16-35aRPc-aNtgUg-8nY3Qm-pwcmgo-azGxDP-a63r41-arhm8c-5bzavY-j3cNpt-8Mggyd-9sGqhc/
Borut Pahor	creative commons attribution	https://www.flickr.com/photos/veni/3553811204/in/photolist-m18PEZ-m1uJNd-6q3dfY-6pY5Cz-6pY9Bv-6pZpLa-6pZq7H-6q3igd-6pY5sH-6q3bGA
vomit	creative commons attribution	https://www.flickr.com/photos/mahalie/276539107/in/photolist-qrkrD-MhdQU-6PWXDn-dQmaLn-dvgGDU-5f7dbt-5ZcQCN-5o3H1t-4wdBtL-Jw2Km-4YDprb-dTiKmF-6TqjUx-4kfRXR-7hyQbL-aAgsi-eRSTks-rdozH-JYm2z-auMm5-7girg1-6tT1DJ-9YGDCq-9YDAv6-peUnVj-dWGy2X-gpWHU-62Af65-5QNXgs-7xbXHZ-3xgWJ-dksd8x-4mmsJ5-5aRYq6-e2Lt1F-b8vQRr-dksfhh-dksf43-dksdtt-dksdiR-dksePC-dkscjg-3WnTSM-dksfUy-5e3sFR-dkset1-6kXKn4-9YFVjG-9YD1rk-9YFUbS
newspaper	FreeImages.com Content License	http://www.freeimages.com/photo/newspaper-job-section-1427231
street 1	CC0 Public Domain	https://pixabay.com/en/road-asphalt-space-sky-clouds-220058/
street 2	creative commons attribution	https://www.flickr.com/photos/cleopold73/3677296594/in/photolist-6AX7aq-t83ix6-uP4F9-5yBcaP-6Q1FTo-jpjRtg-uo3har-81ZGLm-dpX1iy-96w32T-h4C6C8-abeAJ6-pDuTrR-q2hKgt-q2bhFN-Ufdf-6V9hC7-4Bo5g-fFHuYV-aPNzeH-p4YsF5-94rNeL-sFnBkM-nQzq4g-8SRNC6-koJy9K-7hCFXm-pMCJZz-iFVAkn-dhQGLQ-u9qvRx-p8DyTh-8av1Xm-dbEDKw-5g6Ne7-rzjM1T-s1UWuq-hbAFeR-fdhBPP-fuEuwj-a6Wbvb-dnjCpQ-kSheJV-edNGQA-yHXwsb-auMXmu-fTsLbG-tfn5Fu-jawCKZ-7RRubf
shoe	creative commons attribution	https://www.flickr.com/photos/85546319@N04/17278653804/in/photolist-sjRBNd-p6R9Pt-tgTDTx-sZhR7y-p7cVbh-xFHN6E-x49Acy-8oTECf-7amLX-xmkD2k-x4gjzi-sZhYDu-x494xN-wYP8CA-nfENFJ-hU997b-uuH7FJ-xjYkdd-xjYyw7-kgUGHx-3DrwhV-g5cUPY-wE5Fyh-nqSRH5-x4gwZD-tgS9UT-x49SMj-4kgBPa-oCNwu9-e3G9Ph-7HCTvJ-oGAHbB-wrnhBZ-9LR1L4-xnrxDu-xoPwZ2-oCNvKJ-7poPBP-7HyXAp-nhrdyz-7psJjs-7hdx7n-7ujsYy-tgUQHT-fHUvQm-7HQmcA-tgARi7-oU1rM3-tgS7z2-x49tiq
cymbal	commons	https://upload.wikimedia.org/wikipedia/commons/7/70/2006-07-06_Crash_Zildjian_14.jpg
chocolate	creative commons attribution	https://www.flickr.com/photos/schoko-riegel/7674776788/in/photolist-cGceKJ-76Ncrf-eVq3Ku-9Pio5Y-ii7f5X-9zy8QT-auw1aG-9Piow3-rivV2e-abejQ1-aLuw5T-aJ7aU2-8c5wWC-6vLXWu-9jrqCx-8xMLrh-actYkc-9kzXHU-9pGkw8-8c2bVB-b4L5pz-94ksk6-fk53dW-actYgz-fk53aw-q7PaJb-phYR1z-yP7tcR-aHnRc2-bDZzV3-a6NnYr-j5bdbS-tgUJfx-hqHi5S-93X75j-9PfxRt-y7ZYvu-8wFo9S-b2FwVc-e7T95T-bn12AH-ozACFv-6g28ta-uMhxo-eU8iBB-tgVq2r-d8pkNE-hDoRnu-texNy1-e7TMNv
slipper	FreeImages.com Content License	http://www.freeimages.com/photo/red-shoe-5-1307917
remote control	FreeImages.com Content License	http://www.freeimages.com/photo/remote-control-1506671
Danilo Turk	creative commons attribution	https://sl.wikipedia.org/wiki/Danilo_T%C3%BCrk#/media/File:Danilo_T%C3%BCrk_-_World_Economic_Forum_Annual_Meeting_Davos_2010_cropped.jpg
nine	creative commons attribution	Mind & Brain Laboratory
umbrella	creative commons attribution	https://www.flickr.com/photos/131260238@N08/16606777908/in/photolist-riu5v3-dg5t61-pqBcgA-6Zcvvf-e7iXqS-4R3pMd-9qHyP4-rwcB8M-6vQY6K-bqnXsf-55XKPQ-9qLyv1-aioB2h-

		dp4ZF4-zgMSh7-oG4veq-kAQTmR-eXR1wH-9W5KqN-aoEwFu-9qjn36-oi7XJK-dovXv8-5THk61-6bi73H-3ec5DM-rA4s1n-9iGnD6-ch7fwA-rA4swc-8eiukX-riBtHH-gCW1j-bZPRJu-e7T7Ap-5nCiNr-2AT6gH-bWGYhb-5xHe9K-9TMMzr-bZPfMS-p8UAoA-mKBckT-nQK3c-bYheYy-bkrprk-fJ7Eem-6bCsrJ-efd4qa-bDgci7
chisel	creative commons	https://www.flickr.com/photos/smallape/6310337398/
two	personal collection	
jeep	creative commons attribution	https://www.flickr.com/photos/precision/373995286/in/photolist-z3PLA-5JkWbZ-5wBeFf-KRHEt-bAivYt-z3PVt-xDFL2-iXjNKq-iXjLnu-dRcP85-7FLPtk-2h18FB-iMzj9u-aKtcR-2h68YG-iXmBw1-98zidY-5UNVMx-r4Te8j-2h6GpU-iXhF2K-j7H5m7-9nn4RZ-9nn4rH-nG8hTV-fJ2R7D-bnoAFd-9wBAt-cwbs7b-ojAo7a-ahu18z-nYmgWz-4PQPPs-iXjMmo-89Cn3C-3bBVqp-nZeGxb-vQCG82-seqSL-7obnp1-nYiRpV-mEjbUg-rXMeNR-nG7yeY-6y4Q36-acq6Zm-scWqWC-rXD4vS-2gZouM-2h6MiU
Eiffel’s tower	creative commons	https://www.flickr.com/photos/132084522@N05/16746636328/in/photolist-rvQTx7-6JuKbt-aToxCD-5An6tQ-dhdzLP-bqQo5c-cGJhn-4xy3S-9vb51L-Ad1iFf-5ZR15q-RVmCX-8SNDML-56XE2m-aDX3VL-56Z1Jg-aHtpqT-eVzAq6-7Nr4P4-7wmUKU-5afkE2-kkuvnj-sgNRp7-9cgjn-Fzw1E-GAsic-btTpyD-8PKRz6-j9Yez5-56Z1DF-gEn1zy-vgpuaA-rBSz5B-fxHiG-dz2Bax-iM4Hp-88MRBP-gH1YHh-ws2LN4-w1wELn-pvXUJS-9ForqE-qRnN9i-AB1xVL-znZJcz-8MwdS2-rBQN7x-rUaD9S-rUaMQu-qXij1C
one	personal collection	
ferrari	creative commons	https://www.flickr.com/photos/39302751@N06/5503014426/
flute	CC0 public domain	https://pixabay.com/en/flute-music-instrument-played-wind-893911/
small ball	CC0 public domain	https://pixabay.com/en/marbles-blue-glass-kids-play-319938/
rakes	creative commons attribution	Mind & Brain Laboratory
tire	creative commons attribution	Anka Slana
button	creative commons attribution	Anka Slana
house	creative commons attribution	https://www.flickr.com/photos/benchan/9520899528/in/photolist-fvk6J9-8nhh1Y-5n7y82-5RD532-6x6Z2C-5jWee5-TpBM9-6ysKzF-5yUiwD-3q4T96-ed7x42-xzqQDw-6nnccv-5fH4RA-324Qp3-6ywZN1-7sNDT6-a8QJX5-PghoV-A8mHH-2Bgyoz-2fP9QD-hSjncu-ibXzS8-c6hmfy-7SMGf7-6nZDn3-h8rbAf-dJvCnE-foeQjB-3fyrj8-5PqwTU-5ATZJe-awAXvn-6isyKH-cfX93-9TCvVR-8fYeLa-7C8iej-6gcgNb-4qq2b3-8PVbSM-54VCd1-4qq2p9-fEr1JC-qbtNV-fvk7Kf-9wfqgo-ojEceb-zLUSav
oak tree	creative commons attribution	https://www.flickr.com/photos/sigurdr/4950694681/in/photolist-8xtB3R-p2SiB6-6aXRtz-9tBAEU-hSh17s-dgEHnV-4p5FPe-6vz4Qp-z7cc8V-E9rM-dHRTCr-dgD4PS-6LQTFb-ddcEvo-94WXZQ-kGSU3a-o39giS-aBXa41-9Xb1uU-949x9G-egwR5o-oaUBjf-ojfbPW-dudJd8-bwTbBc-hULHQR-q24ZSU-nbvNjb-BNTegY-8Q2fvu-pKzbiL-5CXUJk-74JwRU-5WYkY1-dgjnnb-9X8cyr-bUzvwT-2unUor-rMFm7f-dSVXqv-9e85yc-7JJvWU-oT4nfw-Fwk9-6poNGF-p5hAoq-anwBVT-dkTdW6-nTT9hS-bpcW1m
toy	creative commons	https://www.flickr.com/photos/partymonstrrrr/5831936062/in/photolist-9TmcpL-92Go7k-gqMk7Q-qv2V8L-bzKRYH-48kSAF-9uQ3bq-8ANqrm-kfU3Gi-a4oQtU-a5TYgu-8fy57p-9m8Y2E-p1JyZD-qXbSpx-6cQexa-83scXN-dBnTLV-kGE7NM-8c4wFn-e3Ui1R-5no8Wp-kfWndv-bbNkov-7Fr4vP-9ATmjy-6L4Rtq-kfUL4K-kgz5FZ-dTSaHk-a2U4Ya-66dqbX-9PMaqR-5wmAYq-bo8Dvm-8YLC7E-7wzXew-dCv17p-bm58pj-9bGBCS-rKuyRo-dDx5VH-e89F1D-oebKEH-9bDveP-7Pud3E-reAXAt-wrYLsv-d5vijb-j1BGTe
small fish	creative commons attribution	https://www.flickr.com/photos/casamatita/13991603868/in/photolist-njoCns-byqc1r-iKFg64-5UvK3W-9z4JP5-nn2NYB-a1kooM-9UyGDW-vvYT5-f2j7zk-5GYKsP-6kz6wv-xn2mDV-9Sfsrz-6qwPxk-5UvN31-cS1sqY-6kDaWG-7tioEs-6qAZsE-5nNW9M-6kz1PK-6kDg3j-6kDfGs-6kDfAd-6kz68t-6kDfo3-6kDffJ-6kz5MD-6kz5Gt-6kDeWN-6kz5tX-6kz5k8-6kz5ag-6kDejd-6kz4LD-6kz4Ac-6kz4uZ-6kDdHS-6kDdC9-6kDdw5-6kz44D-6kz3XT-6kz3Se-6kDd5o-6kDcLf-6kDcqJ-6kDc75-6kDbPy-6kDbvo
injection	creative commons attribution	https://www.flickr.com/photos/cookylida/3179294597/in/photolist-4o1Rn4-mJFas-5QWHw6-7KFRF9
excrement	creative commons	https://www.flickr.com/photos/snowshoe_photography_alaska/21600768491/
screwdriver	FreeImages.com Content License	http://www.freeimages.com/photo/screwdriver-1624370
apple	FreeImages.com Content License	http://www.freeimages.com/photo/apple-1556445
baby crying	creative commons	https://www.flickr.com/photos/thekmancom/2674681455/
woman crying	creative commons	https://www.flickr.com/photos/dionnehartnett/4988331495/
baby crying 1	creative commons attribution	https://www.flickr.com/photos/joeymarasek/15039605842/in/photolist-oUZUR1-b9gvxH-fmYDR-21sREG-6Em2P-emN4Hc-5vz85j-26pCje-fAsGBe-c3wbi9-aayNtG-7VGN8P-6jbhJw-4NksMd-ajha2-21jqJ-2t4r2-2Ev38Z-7CVPCs-QqSZe-x6214G-y3VhKs-7Q6w-8HnEEc-covUyU-8412Zv-7Ws8vU-5B8MHf-hy98BR-eFD82-6HqGxe-8Qcyvs-6dxfrB-6ovvwA-6dS1VZ-r4Zmr-3jDYmZ-5NVRj7-6bPYoY-8HqNoE-wMcHRh-anQdck-H3sP3-wQbMZh-jtoWP-6i4uSw-7DPTZB-HMME8-zhooc-6bKNox
baby crying 2	FreeImages.com Content License	http://www.freeimages.com/photo/devansh-1243873
baby crying 3	creative commons attribution	https://www.flickr.com/photos/120077902@N02/13234990425/in/photolist-mawMy6-j1ff2-692vuA-aDThu9-9jRaKV-26xSer-5xjoMv-9tjcPE-9JrhKQ-9Et2o6-5LuT6L-5NxT6V-sySPC-nqRyrw-gjepHy-9jUfuS-8upZVH-7eP139-9jR9TB-8nTxh6-5Aguqy-6BR2Xp-7MoJoa-a2bUCw-9tjcrE-9tgeLp-53rpJP-dgYpfp-fss9JP-bENc6Y-h4kju-jKRY5-6gtQoT-9jUfiC-5gJPte-oMEXT-s1hQk6-9mnXBz-8ypBNR-5VQyDg-4mL8wg-aCneMg-jpRBy-rKXEHW-8vGnwi-bafFgV-aGZXQc-7rBhXj-79tQPi-56i14z
snake	FreeImages.com Content License	http://www.freeimages.com/photo/snake-1554005

stone	creative commons attribution	https://www.flickr.com/photos/rvoegtli/4639855198/in/photolist-851tiq-9PUSo8-9XqMzY-8jVeLG-55fvmF-By6xP-xz7oNd-p3vK6W-9QjZWW-7GabtC-4DE2Ai-8qxnfn-bwNTUD-iJoGwd-664bVJ-awEMzV-91P1Ed-d6wR81-6vAZQt-6KkjvP-7dgUd-vkE6oP-9pMMx4-vJdtU-awn9A6-bx8kSd-66riUD-8hJRpD-dBssZh-9pZ2PF-AyZfz-eRqJg-91KTT2-4YWMJN-8hN73E-bxkgUK-5pp7oj-ww2E9-5uctPC-8hKqEg-tXQcV-9R9Yrw-vk8FLq-5QbaAX-8hJRnk-8hJRjZ-9BVVzC-bq4L3P-4bKbYd-8hNUtN
kinder surprise	creative commons attribution	Anka Slana
hammer	FreeImages.com Content License	http://www.freeimages.com/photo/hammer-1561230
key	FreeImages.com Content License	http://www.freeimages.com/photo/key-1568411
hat	FreeImages.com Content License	http://www.freeimages.com/photo/old-hat-1421327
bicycle	creative commons attribution	https://www.flickr.com/photos/brand0con/4265664591/in/photolist-c7RkHE-g8Bktb-kTQc-7uWDte-nSYHtz-6LFf5r-c25VT1-dM2xfw-9zUgup-ar3gDn-9zUft8-o9g66C-9zUk68-o9fZb1-oqJ7Zu-gCJnL-9zUm7p-6z9XtM-o9g9f1-9zUngx-e3hBXN-7TpHKZ-nYybTX-6zebrA-7T8sgz-4AsD4e-chrrH5-nX2AJW-oqxL8m-5Vydfh-4JVJWu-o1CMC8-nYxjXz-9zXdgy-yhtwa8-7zVs6R-fAtYsq-6Uem3F-9zUjuH-ef3sLX-5VtRuV-5bu4Dz-rswP3u-5VyefE-5Vydd9-4ryZ4a-ef3sVK-rfdxFs-oYWC2a-5Vnyyw
carrot	creative commons attribution	https://www.flickr.com/photos/vialbost/9140280979/in/photolist-eVGk6n-7hAKBE-dUKDyr-7h39pL-9RgPYB-9RgPXP-8NXjSi-axJL2o-avNCZ1-4PPRge-bYigVA-bZioSf-66VzBW-mD5RNc-mD5Sn8-6R3pat-imwbBF-bJMqZT-5Guvs7-5toLDr-9dDPej-wofdq-5JzyXT-9c3b9k-bwXmP6-v7cCyw-9n2iHr-7MMVCR-5deq65-7U6Ed5-nxihRj-fCsmjr-fCsmKx-ne7e7a-qgqWRN-5Ez7Kd-imwMQK-6BeQxy-7JbNU3-a7y2Yw-aiajja-dctdpr-nekqVs-9eSWjj-8Z7Wnz-7igAzT-2Toe5r-8Zh5RZ-4BKqxp-7MMVAP
suitcase	FreeImages.com Content License	http://www.freeimages.com/photo/old-old-suitcase-found-in-the-loft-1544849
cow	creative commons attribution	http://www.flickr.com/photos/adstream/2446660939/in/photolist-4JcM4i-gU9Xap-gEyJt-5TZTV2-6qT4Y7-8wXjUE-daeTsQ-8obmSD-5Y9xxE-dLc1zq-oK8KUa-dMTsgn-o6RAt9-2suKGh-bLmYfk-nBM8NA-6kzZbk-beYJdx-nBM64P-3tigMj-fexs3v-hoHhLY-ug6XQh-8knLeE-eZeT95-oH2Xrr-nT5WaN-fZU21e-oJCi2D-up6sU-5hBUnt-54ZCYv-Ax6cQ5-2iTmSw-8bdaA9-2spGD9-5tAgEk-x2ie9m-qffiYC-p4R9MG-oaB7zb-dvnZPc-fYGgzp-cYmKXN-2y139z-6kotjP-pcyKaf-pwVSAX-aghf7i-jGS9JK
screaming child	creative commons	https://www.flickr.com/photos/kessiye/516354653/in/photolist-MCsfr-5rZm5D-4y2Jor-6Un1H6-iJKXP-6uZdMo-52AnYk-47wzg5-47s8Wv-47s6PK-47wuSo-47w7Yw-47sh8n-4XDMz5-o8cugC-5hyFZW-d2qt37-d4T9Eo-9fTdYT-47w78C-47wefs-47whjC-47ws93-47srUa-47woTN-47w7Rs-47wgCy-47sa84-47s54i-47spAe-47wiJQ-47w5Hd-47sAjR-47wBud-47wfow-47s7vZ-47sej8-47wdNu-47s2Ur-pZZf3D-b2S7JF-4wmtsD-5fr578-5kQupH-9T6uSv-4eDje-dNiPSo-4wmtbi-4wmv5Z-4zEg6N
screaming woman	creative commons attribution	https://www.flickr.com/photos/jumfer/8168188753/in/photolist-drN6Wz-dZzhWF-4H84B8-qsP6cq-3vh2LK-bYhf4W-6cGVpw-3mAUt-25LnGL-25FV8z-b7zFgi-7zrVNZ-5HMQVk-sJWk1-9AN11x-mBfdZz-qdb29d-4rhMpg-e8wP5L-owoWTG-4m9PSt-oB1VMk-cEn5xw-4r8X4g-d43Jhf-4S3oTq-5nn8gg-7byFRE-87uQVq-naigT-4RYeJk-ePsAW4-33ajPv-8kcaAL-6kbdPd-dkXmbr-dv7AKC-8LW7pG-ac39Yp-6f1UU9-dMzF1R-m32irn-8AgCog-6btB6k-4kbWAD-8dqTmy-6ZiLz9-4RYdHc-8zEA5X-5BK5qi
pen	FreeImages.com Content License	http://www.freeimages.com/photo/pen-1568529
reptile	creative commons attribution	https://www.flickr.com/photos/papa-t/5756776648/in/photolist-9LGZ8N-frmFar-fqPHW7-9G8iya-Le6Ye-9C8JFa-deghFa-aCU1pM-9wxRHZ-3ftrxQ-9ztoRS-9zqpWc-bNpA3F-8keu8z-okpxAH-nGwDvA-rQDzqp-8eL8c2-9yaF3H-fPFgkG-efkq1D-86NkAs-bsGncq-5mwt5b-uHSfxm-8etwtf-cDKTUY-bz56MW-nfzocG-9nxApz-s6JhY4-hzSihc-4Ui2yx-twttL9-oZi4u8-gnFWvH-jkDwf-wsu9LA-9wAQzu-oHN75s-gBmX8w-9NhwZ8-gMnpzN-bRNhUD-jNfiu-bsT6h3-5y2jFK-oA1a6d-e6tX45-9uEayt
hair	FreeImages.com Content License	http://www.freeimages.com/photo/hair-1424731
leopard	FreeImages.com Content License	https://www.flickr.com/photos/tambako/14105559572/in/photolist-nusFqY-gXRJZ3-dMXQy8-4TRaQK-ojbk4-dNh6K1-nAsCMR-dP4Qya-o6rMA6-ofUf1i-oj1uB2-cMBMLW-82hDsc-cGqeam-bpi7qz-9sY3vR-g2jsXw-e7VH1L-nSHcsm-asTJ76-9cnKyb-iw6oMb-hQonEP-i2e3jN-iBKdpk-rxdp8j-bJy9AT-orb4Vv-cHmuu9-a4nDrm-ato7Tx-rCAaPC-j2Xru2-9Kies1-pJjbr1-i6NJBG-ecM4Rt-KER9V-cSSjfU-hKi4qd-o2N1kG-o3okkC-oswPwq-a4o8ED-5ZFLkv-bKEGKt-d52Fzs-8kVQEp-f8KYZh-hQCPyW
leopard_1	FreeImages.com Content License	https://www.flickr.com/photos/tambako/8594099800/in/photolist-e6r1su-edvVQv-fpwxhh-dNXGC5-5kcAGc-e5zTdd-9ZTSDo-6BsL5s-ar8pYj-bUGz4n-2SaaJ1-5PxDYU-o8d9bJ-u2rUqo-584ZKR-9VAa7D-62oB4X-76qrEf-65XhNx-2GnTb3-87YTQm-ghafHB-4jQKdM-7EAbWU-bDHohb-3qFxcX-dVDuhp-GEVPL-xm5hyY-uyfKkC-ondszo-bKqFDF-7EwkAt-einBhq-bL7GUZ-sNBQFh-dVSct2-heiK7r-opfr1V-bL7Gk6-6dxZMw-5QLHdT-vREhs-3HcXYT-cfW3fd-5PEptQ-cv2Qrm-3qjmWX-7Uha9b-3MHVuY
wood	creative commons attribution	Anka Slana
ladder	creative commons attribution	https://www.flickr.com/photos/stephenrmelling/12831392255/in/photolist-kxSeSX-9svKir-6KqgFo-eK6kZC-7T45Xb-7QSFTX-auWzPx-dvtkLt-6s1VkQ-5hUNjZ-9svJVp-dT7kuK-nznpm4-ne3nKd-niKPXN-eiYjrW-5K6RxZ-ebx4Xj-snmQvE-ekxKRP-4pzgsL-8WUsAw-vYZDMG-4zYZqo-5pk3qk-9iPHnf-5j1fBz-uPTrdC-8sAcYd-dN6zSv-ap54qM-78pH1E-8Cwdxu-kxSAoK-X5kZs-5U6Rer-bsQKVP-5FkWHE-7sFFKi-bkEQ9Z-3gs75o-ad8Yrf-5FqFX2-5qcA2Q-4akjtM-nx1tPe-eiCYRm-cQQ8uG-7LJ4To-6V4o4H

lion	creative commons attribution	https://www.flickr.com/photos/tambako/8465301216/in/photolist-oWWXFx-9mdZV5-8v4tg-zdrvgC-dU3T8f-bEDsqt-dQFLG4-oWWWSD-7Un8WK-8Yxo3e-7Un6yD-zX337Z-9uWBCL-9uWBFC-5q4wd5-aMYMzz-xbd2Eb-9m4Xt7-6qhqqb-4xUCt5-79WA6M-9znEWP-4bMVsv-7h6rWa-7Un76P-s9RWvP-dZ1JV-bFyrrH-oWWZPk-a1NKVA-hj7eFK-a7oonu-i1dtk-8BDAMz-EcPJa-a62CRc-JND81-DDqFy-8JPSwk-fy7wLB-fzRBoN-a7op8f-okiATG-8yf5A6-bxHpAg-eFCZfd-77QEtw-5UNjHz-5xKUJo-x1rH7
fox	creative commons attribution	https://www.flickr.com/photos/unicoletti/7112974337/in/photolist-bQxRsz-uyKG3E-de5PrK-azrVYq-brwYBP-8zHUnd-JuJpg-3Ao6a-enojkX-3AmsD-5BP8sj-eSHcKY-fQmG4n-3Ao68-bXAibq-3AmsE-3AnNM-cLKNq7-8HKevz-dZ8Xes-3Juyt6-kMwed-3AonV-7Npcey-inSrc-eX1vfP-dVJgfU-vXRcsq-9vjzEf-3AmsG-dpYiNP-cYokyU-vRjgm8-azrYmN-tgxpiE-9r4utL-eHXf7n-3AmsF-3AmsC-3Ao6d-4UunLm-fzZNrf-uyx7F2-ej1N2r-EBU6a-nz32P5-rFq5nx-gQxunh-wKzutT-jJnM3o
scull	creative commons attribution	https://www.flickr.com/photos/dannypigart/2682192377/in/photolist-561Wma-6uPkTN-cPczgG-fn8ZQK-29kDnk-61XgWi-mScic-cPkMc9-7LPJyV-9oWupZ-6sKfMB-byFztg-9rA1RV-bxGWPw-oa1Hcu-2h1wxD-bM6GC6-ffNX3G-9hfS1U-fPF2tw-4JXVQT-9hcHBZ-afG7oo-7VyUd1-7966e7-9tS4AC-6WfGPs-oTe8EQ-5Bm2yY-7APeSe-e1Cx3b-4QM47a-9aZR5D-fV61RH-x17WwJ-8Z2KbH-owBQH-aWRMmr-4TBjMo-2gR2r4-65d19r-ayrfXM-ibSgkM-9hcMQz-LZgxK-cotPU3-bCJrNe-6JwUrF-7967Jb-c5gEXW
flower	creative commons	https://www.flickr.com/photos/witnessoflight/21216719632/in/photolist-yjReyU-9TiQi9-hNbRo-7SdA4T-7a3JiJ-45mw1-3w6g6-fvVLuD-367Jho-nATwf3-ndDxgi-nFHLpz-f5XMrx-86rUdr-2CMy6j-3tYQE-wEkjKL-JVhPf-i5PVi-aiUNRt-tsM2H9-93fV5F-uTzx3s-2hKZkU-t7Yb34-iu74NR-5frHY1-2jiC34-8qMcRP-4MsyRs-nbB1yi-nbAZxv-4G5MB-897DYM-a2VRHq-nnSYD-6NdB2r-3tYQD-s2m24Z-xcM95m-ajgDVJ-kc4B5F-pXeJJC-r562zD-RoAe-6XCCii-4yyHRe-8qdEp5-apk374-9aWWXS
shovel	creative commons attribution	Mind & Brain Laboratory
cat	creative commons attribution	https://www.flickr.com/photos/mainecooncastle/14129834792/in/photolist-nwB6Bf-vxMGz-nwSu3a-fd7sCV-evCjfm-5xUnZB-dnEi2v-mY4ibA-ei8aqZ-x1E9CW-asHHnt-5xVwgU-ehMkwT-8ryPqV-81vk1-nfnxiV-5sswRw-nfnNu7-9mAZhU-nBeGPN-nwTFXu-bxkzx8-4GvMSN-bMLPtK-97Rdr6-62qEwz-aZdQHV-dZU7gj-aTBHpX-9vD3g7-XR4Mz-3toTt-c97s2w-7VPK4d-FHtxS-9AfoyW-9616VH-cUPhgC-3eCC4b-rZxQZ6-6nAufn-deAxei-bpJu2H-asLmxC-eDFXL2-3toKA-6j8y4k-55zA2d-86sunQ-8aLTGV
T-shirt	FreeImages.com Content License	http://www.freeimages.com/photo/t-shirt-1426871
massage	creative commons attribution	https://www.flickr.com/photos/essexresortspa/4098831412/in/photolist-7fczM7-oYvXCa-8rhH5Q-nYAkwH-7wxWVB-bjFTta-4JHFr4-AshVYk-9YLuSK-azdunZ-9uGrXT-9W59kA-6Aw4BL-mf3z1A-mf2tXF-4fcvj5-arpbwX-4uekgE-9W59pm-68ZL32-9azosH-4vnLGw-cLSho7-9K1AKa-aszjs7-ejLZCq-61AYLt-5bQ4ww-dB3xy9-7sZRqK-g44Pwf-ovPZKw-aCXtBW-o7TtSe-zXWFEN-pfJgJp-iMebAR-8shtGs-8seqgv-7GM3FE-9d4PDN-71SwLW-hphyZp-4cAeLb-5k7yQv-8UWK3G-6MmE7s-75fYoK-7PtUWe-RgkT
bubbles	creative commons attribution	https://www.flickr.com/photos/featherlite/3482830636/in/photolist-6iLqd5-2hXkJS-bny4sZ-6WYjhQ-c7oHAb-7TdXYm-4WCTgi-3rnN61-9Z9UMD-juWYmn-6UvThG-vkb9NC-oJMaeb-9B9Nbp-oqvjqU-9Ld1VB-6k3MfL-ho516Z-7e6Eto-dxAqag-f3VhQ-mJh6A8-ztMoZ-6Mahzv-oNS2gK-9uuatG-bVn1o2-oTaD5g-7Th9X6-q4RohF-ocSXjF-jGw9yT-qnwFgR-4hi378-rJuaBn-e4PpRC-jDm2qc-oeDX8v-beppUn-etFqoV-dgda95-etJxvL-6wQwcr-6iLqgu-9nkB9K-nzZ5c8-n9hVjG-89QFwM-4VzLf7-8bVTzG/
mouse	creative commons attribution	https://www.flickr.com/photos/127787488@N03/23784878579/in/photolist-6cqnP4-9KoaWU-cDxomb-b5ZMeD-6NGKTb-7DJmY4-ptzwDn-9SLBur-8Neo3T-oYTuTY-8psdcd-doCXBM-fM1v18-c4rL2s-2hvvWx-9zW4me-9AJTAg-7wHtcj-rvCgjk-dxuGG6-bvC1hv-e3qFe6-9yx94k-9CWTKB-e69bdh-94Wgmg-9pLciz-eGiyLc-5s12Lm-oecDwc-dHfYkp-CLytPr-hcymff-94Znom-4oLKan-bzet24-ngahMk-9YiDpX-bt9w65-94ZjkG-9jG3qa-BaJxUe-e6K9mw-CeMHKr-fU1yPV-z2KgEv-9noka1-9wKcTG-4PWhDx-4ERncA/
sand	creative commons attribution	https://www.flickr.com/photos/davidstanleytravel/5282834545/in/photolist-dvgYSs-dUeQqd-bsdXvM-dLgTu5-dKv7Rk-9nxxRH-pkuwu2-6Rq3rw-93PUEe-dUj5ik-a8cUFD-bmdFAG-dR6XVD-bqW1YN-6w9Rc2-qSCdTJ-dTWhuP-mimKqa-ww2Qa1-i42xVb-9bXf6r-bF69Tp-9caSez-a6xoU6-qWN5Lr-8FKQ32-e4pzmd-dUewpQ-dvAS9i-4YT3zi-9svcTY-e3hA9m-nJkVv3-dzv3uG-9HDyWK-auhTnV-amuNXw-845j35-pk3CWd-5Xvg1a-rTtZC4-9yCSvT-d12Wrs-9vf5xV-dPddyT-dE5YnP-dUMMSR-naR6zY-7uxmyG-du3YT4/
table	creative commons attribution	https://www.flickr.com/photos/mcdowellcraig/5451576082/in/photolist-9iJKD5-h1zoao-9iFC5B-dvA7FW-dvA9NG-oZMpE4-isf1v5-JpHmB-nrc4om-o6Cppf-dvuzwv-9iJKVE-eBZCBL-is7vgN-nv8ACS-rqyzZN-dvAapW-8e1ZBC-dvAaz7-vNj4Na-jxB4yZ-9G6AXf-dvmkcU-j19vVW-9G3Gz6-dvuzcc-dvASxb-bwuiFr-dvA8CA-dvA9XW-dvuxbV-e3iyuD-e3gbEt-dvA8TC-e2Bw5c-rzevVb-9ESdXj-dvuzeV-bqUvsy-8HduqZ-iYpBFq-iYoKRe-iYpBPS-iYoKDF-iYntmp-iYro55-iYnt7X-6pSoee-ch6pao-j2SjUP
sea	FreeImages.com Content License	http://www.freeimages.com/photo/sea-1555628
sea urchin	FreeImages.com Content License	https://www.flickr.com/photos/kuw_son/305101168/in/photolist-6mLYS1-bX7h9a-buKuTW-4wMcJK-9bdQJz-9bdRhx-5n4ACe-4Q8x1Y-efacTm-5PSbGf-ar88nV-9UYfXy-9UVqEn-aam7om-9UYg7o-yMRPLM-mESCGz-5PGtXW-o3A4bb-9UVqrH-7mHxdQ-rVZpkG-6LLPRY-31Q5F-bwEWgK-Atu2b4-sXHWY-9UVqjt-9UVqvT-721UAp-ciY2xs-bEPcd7
fish	creative commons attribution	https://www.flickr.com/photos/noaaphotolib/5187495241/in/photolist-4EaFi9-2PbfaH-6fNz2X-6fNxPD-a21bpR-hVukWC-j8xtzr-9XSrdN-6fNxRF-6fNzjP-7x1D3m-hVu1UD-4qWcqz-dprNnW-dprPNA-dprDZg-6fNxYc-6fSKab-4xuEK3-89TCzX-6oH6qB-dprLXm-6fNxUx-6fNyrz-6fSKyU-aLYU4K-9BcBXk-bgGmcc-5obY3T-5ofNjN-4MCNfE-8TTiw-8z26pL-6fNEmt-5ofRKb-5HhgQx-p31Q4F-p3h942-yb9qKb-5HmFg1-9hrX6g-nDuu6Z-a5FNGB-8UpgB8-mKTuu-6QsJdZ-8NtRb7-9FWU6C-5Ns5Xf-6fNxbk

shark 1	creative commons attribution	https://www.flickr.com/photos/rling/2327704400/in/photolist-4xG6qu-7oqBeW-35PD3k-e268Rb-4vqPRA-cCRXh-nthdyE-EH4pV-aGHG-cTV86-5SHw9a-8otLek-cJbsuL-npexcu-7yC2ij-82MCLg-oHVLa6-oDabKh-bhDJxt-8yKpbD-zojgfd-mtoGZ4-sqH6P-bgLamp-r6qEAv-mEpnHj-72fZGF-erkZB1-EH5gK-8AXyR-BxPnn-piaLpp-6MNm4N-jpk1wX-ncdcrN-EH4pr-e7qh4b-4Fv7NG-diDRts-cCRFX-9jfrTo-HgfNf-qKMPS1-rfNCmo-nTS63-oHVMP8-9RAQ2J-8ddg9W-EH5rm-kjs9eh
shark_2	creative commons attribution	https://www.flickr.com/photos/the-lees/134610871/in/photolist-cTV86-5SHw9a-8otLek-cJbsuL-npexcu-7yC2ij-82MCLg-oHVLa6-oDabKh-bhDJxt-8yKpbD-zojgfd-mtoGZ4-sqH6P-bgLamp-r6qEAv-mEpnHj-72fZGF-erkZB1-EH5gK-8AXyR-BxPnn-piaLpp-6MNm4N-jpk1wX-ncdcrN-EH4pr-e7qh4b-4Fv7NG-diDRts-cCRFX-9jfrTo-HgfNf-qKMPS1-rfNCmo-nTS63-oHVMP8-9RAQ2J-8ddg9W-EH5rm-kjs9eh-bs5YmJ-5kKq6-cJbBPq-9jfrT3-3zDA8k-dVZSk-kfL1m2-7zd9X3-gCBWtC
shark_3	creative commons attribution	https://www.flickr.com/photos/134222203@N08/20730963375/in/photolist-xzVBa2-bmeZcy-r8fuUw-bo7zxB-rcCUr6-snRpm3-sKesRr-s7LuXQ-rcYbev-rMUPuo-ymNFbC-8BB9hJ-r8gVdw-8emZSE-6jWS96-qVcZAY-8eiHtH-9DfeEn-dobkmt-rf1wMe-6sf5ua-r8d663-qzsUTB-rbWS77-8BS2iu-ttdX56-qhGThq-rMW3ad-qfH18m-qExAux-qVwZGb-egW4Mo-rwb6SB-5kDo2X-9njggv-rjLuDs-qUwjqk-fusbXX-8APzko-xMMRt9-rpHtNg-vPt88-nQjUvc-dobruq-aDt9At-5R8jFJ-o5hvQp-nx1Tqc-5X85SE-oD6wc8
man 2	creative commons attribution	https://www.flickr.com/photos/maylee213/7266792026/in/photolist-c59d6G-4De5HP-dadw1f-hKyFNo-r1m5oi-r9BtEh-eMN3gY-qVy3yJ-qeT7Hm-5eTr2W-rbXTgn-rcYkpf-qVDhTz-rfNhfV-8emZ2j-a5UT7s-rbThM7-73Nk5N-apN2yQ-qeNaWq-tpt5Ro-qVCkui-a5iVmG-qeYZP9-qg3msF-qVE9hV-74nMHn-8emZdm-gojAYo-c4yVdw-eib7VU-4udPPM-74rG8s-5xpBr2-qWyvaH-qf1DT3-rcWPf8-qUnQUq-8emZvd-f93Htq-8eiHmF-qgiPLP-tFMrTG-rgjxLy-rbUfn4-qUttQw-qWBhHv-r1ewsC-qUqCx6-4w1uW4
dead dog	creative commons attribution	https://www.flickr.com/photos/vintagedept/3370128351/in/photolist-68NMKe-6mqXq8-5uaEyN-J9eDg-iGBZ2n-4j1ZpA-5cvTVT-DwdvX-68A4vH-bEPwSq-nkL1-5yWvrw-2zwJC-9dPDH2-53ZBAV-6xGMSb-aE2pJG-atK4y3-atK43u-atK3xQ-atGnaD-atGmze-qR4NPr-r8uB4V-qQVC4h-rbvTtD-AN4TZ6-2bn8pS-a6kBBw-j3YE6L-ekCdr9-887E3B-6bUyQ8-gEhBV-cSYG2-dT8wPo-iQXGc2-pGvroA-8mf8Z2-5yJxS-3WaW1-jAVdg-cqEK9-7dVPvw-eFTkTk-9wYhhA-7WtXCC-5V4sCK-xjiEYT-oW37Ne
dirt	creative commons attribution	https://www.flickr.com/photos/f-l-e-x/850204185/
socks	FreeImages.com Content License	http://www.freeimages.com/photo/wool-socks-1-1565030
hanger	FreeImages.com Content License	http://www.freeimages.com/photo/percha-5-1417281
hug	creative commons attribution	https://www.flickr.com/photos/lissae77/7884276572/in/photolist-d1GYJY-rQViho-7jcMbe-2JhzW1-69U8r5-rwHeUP-5i84nw-oeRgV5-wPnWp-aqJrfq-oy6ZeK-eiDrrE-6eLDLT-fCZgWv-nHMHTV-nAreZy-rQVifE-rwHeTM-69xAtF-69xmm2-5kXiFe-5kXkbn-5kXk9x-7jgENw-7jgFm9-qU3nn5-69BEgG-69i5gZ-iPWSvX-5m2Asd-5kXk9Z-6BCeW1-fvCSFG-eK9zD1-rJHdS-69BvTb-fvCdem-rdWvNu-4fUqN1-6zkLTC-rBe8Mj-eeiQyF-qU3nsL-8EVwHM-njTtNM-bza1Zu-8uHH1M-nHMVzQ-nAnVct-69xpQv
blanket	creative commons attribution	https://www.flickr.com/photos/131260238@N08/16800383102/in/photolist-rAAmzC-dGNGLA-vMHaY-psE9Ng-rgJTQg-rAAfAn-rzXzoB-rhoCFn-rAAjiJ-rA4hdM-riv6js-rMz3Kz-o2pzsa-bWUnRW-dXqozC-rxLLg7-dXjHmp-rmJkTg-9HJQuq-bWcF29-7PyK8d-fwZHEr-4u7Zwd-8XWtys-iZ76G5-4QM47a-iYGijT-aoRKpk-kmb47H-iZ7c1w-88nS1n-xMXZj-9k1cfd-rbgF98-9eLzwV-9nMaBF-dFTR5U-iZ75F7-iZ7b7Y-iZ79Vj-eibVHJ-iZ6iP2-7oCxwr-iZ7buG-iZ6cse-iZ79yh-dXjHr2-8Rev3h-iZ6pYX-iZ7bV1
injury	creative commons attribution	https://www.flickr.com/photos/zervas/3849855910/
organ	creative commons attribution	https://www.flickr.com/photos/brokentaco/270516180/in/photolist-pUt35-iA2FxP-n2jYZ-4pGaru-bCvaki-8dS4F7-eZGDVM-y8EtLn-8cZLZm-cTd9FA-cTcR3E-7XLp5f-6gkSx8-riSYg6-9xfvsh-jC2eDs-5jSVf9-ncrHnP-93MtgM-n2ffS-kqWGC-9p9deB-swuYh-c37nb-mxuMo7-mqRcq-cTd3Gw-68nthZ-bCvagn-9iXAnp-6RpzTg-en6hs8-c4Pf33-g5G6J4-23efAc-25YSzC-6TGVRz-mtMzA8-9WKeGn-enEVdS-daLHdC-daLH8h-en6hZR-8bw4rk-6QBdZw-kV25M-y9UTNv-6AU8Q1-fwGoaY-fUaYsb
donkey	FreeImages.com Content License	http://www.freeimages.com/photo/donkey-1380444
eight	creative commons attribution	Mind & Brain Laboratory
sniper	FreeImages.com Content License	http://www.freeimages.com/photo/shooter-1431402
child	creative commons attribution	https://www.flickr.com/photos/limaoscarjuliet/3921970684/
child_injury	creative commons attribution	https://www.flickr.com/photos/stopdown/2541642099/in/photolist-4SAzDv-bCsahx-bCs7D6-g8d1dq-bCsa5v-bCsaGp-2uQMG-g8ecE9-g8dCss-943WZH-a4PdMs-67kXYt-24RSN4-vJTDGx-g8cZYs-8U7xUh-bCsbQv-uEWU2W-uXJgUt-8U9dMt-9RYLAm-9ZZRS1-uX484m-u1HWcV-9RYLBf-bpxcib-uF12D2-uEWytq-dBjeqe-8KGG38-bZBhoW-g8eyhR-bCsbvi-7agAHq-fZgFX4-85kPcF-eP8wta-g8di98-u1t2qm-uXNzyP-5qz4DE-5NXApN-bZBhW3-g8cWXB-g8e4mK-bCsaaM-bCsbyV-bpxfew-bpxbBG-u1rK4W
child 2	creative commons	https://www.flickr.com/photos/ajay13/15701703866/
tomato	FreeImages.com Content License	http://www.freeimages.com/photo/tomato-1326722
feather	FreeImages.com Content License	http://www.freeimages.com/photo/feather-1540813
dog	creative commons attribution	https://www.flickr.com/photos/jlhopgood/5547719994/in/photolist-9sevSJ-cKC87s-6yDeZx-fb5kq1-xr69gv-wi1XyN-wXyxe9-7GuhBs-94wFLW-wXsscX-cKC9nN-5Pua2P-9URQPf-bymBCM-8vn2h-5PuaGe-9jZPUe-da2M1u-exSSU2-5PysL1-9LCrfn-5VfTUu-6W2s7W-wXwZFa-8vtZu-oYWmwM-8XckPy-9aRtNF-faQ6jv-7xDbeB-faQ5R6-fb5kXo-xekhUE-wXguGY-

		8QQWoT-b1ULZ-9w9CGS-va4vcd-rUJz9P-76ihs3-xe8CxG-cfVngL-cKCijh-5FYsPf-4AZzHq-cKCcPC-5quroC-5Pyu25-AdmtQ-dmasRx
five	creative commons attribution	Mind & Brain Laboratory
rooster	creative commons attribution	https://www.flickr.com/photos/hitchster/5442716231/in/photolist-9hXkV2-aWRPCR-e4vw8X-bpnhho-eyDJ2E-aG8Yd4-4iL36G-9ooJuH-7iA8ks-gsYQRK-6DwaYs-cBNh1U-dsySQn-wjtUtP-bXhSyu-YyWBT-f8rU12-6faWvv-aWTJjK-aWS9cD-axtskg-8YkqZP-6wV83Q-ozQxz1-7XXB42-6wV8Q5-cEHD8d-64MoWy-cBJQDE-7Wey1c-4Qhn9u-bXuz6S-4Cyq6Y-48sP6S-bqzTzQ-5xWqih-bXhDgw-pc7YwY-7uX6Cp-nQb4yb-b8Z8xx-a2ibAM-8cNTYH-5RoTX9-aG8X92-dh1Bon-5hq2Lr-9WQNwA-5q1s8W-5qwDBj
drink	freeimages.com	http://www.freeimages.com/photo/glass-of-water-1327027
pyramide	creative commons attribution	https://www.flickr.com/photos/yasinhasan/3829314547/in/photolist-6QoeNB-dfNgCw-bG1Nkz-gmjsz-aVKbGT-sEd6x-72Pseu-cagMaQ-e6bFjU-4nTfjZ-8Rv6ZF-6EGmYf-e3S38D-N1RnX-9RAYbU-8QQNGQ-jEpmRC-spv3K-6jN6Ai-qKX69X-6Mq95w-8aMZbF-9X6Z11-88NjJV-5ykyQg-Fugdv-5QWpPJ-8Fz8Zu-mYb1D-9gTmE-dKmzrL-5hpZ9J-5AYL8V-qM3jJi-8WrzM-evcFGR-6RV5z-5R2vmy-AyGbdm-sgNmP9-58U9ha-s64Nyo-658DSY-5DgLde-yZWzg5-9fWBAk-e9x9pU-9nBs2E-8pgk4a-4FDfRW
Pisa	creative commons	https://www.flickr.com/photos/yujin_it/2106118080/in/photolist-4d7pwG-4D9JMF-7K4KPS-H4Grb-H4GSw-xEE29T-xE3cZB-7K4R6b-wH2bdm-7nYsG-xBHvmw-5eifn6-fv4puH-7ANaPo-wH291f-wH26K3-xnqvcq-H4Gvh-i61tvP-7NDxoz-xE33yT-5xZuoa-5xZtwv-6KXWia-7GWLxY-xnwUMt-23Aios-6XZ5x1-5EriYX-4HC7Dh-xuTX6-g1TZTN-2y6ZuQ-wHapMD-kfVUv-wHax4T-a5unFA-xnqBYb-5xZtzr-HLmeP-8iDsZ4-8UMBbc-2ryH8g-2B2Xo-5y4Tzs-5xZth4-92rCm-G6GQ-fviGmJ-wH2cN5
gun	FreeImages.com Content License	http://www.freeimages.com/photo/gun-1493106
pizza	FreeImages.com Content License	http://www.freeimages.com/photo/pizza-1570323
swimming	creative commons attribution	https://www.flickr.com/photos/frankcomer/4826831425/in/photolist-e6EKzT-TMqqB-fnvrwj-4f2J3X-4f6FC9-4f6J3E-5Jfc3J-58m5Am-5TKu4w-5TF8zr-8mwLPt-5Jfcsb-X83cy-ndtZB3-kr72RL-kr9LNd-5JfcBf-6KQo6g-bKvCcT-cyCxow-9Y16U9-7bsrz8-7DNrfJ-fpMrax-cQHTAw-6xEH2D-5q5JYk-todhCq-axFanP-nLMxkn-e2DesE-87Zxkm-s4ft8u-2fyZ5q-5TERKZ-tsw2nT-5Tktt1-9mmsw2-nqfhvd-aq68jE-aq3r7v-2voJBj-cQJ1aQ-oaPERT-5TETy6-aq3rbH-7DJB8t-e2Devb-e2DqSs-6dy9QJ
beach	creative commons attribution	https://www.flickr.com/photos/jopoe/5016610618/in/photolist-8Diryw-mKa4p-moEdW8-ekwUSj-eY8zP5-p37JJw-6eBKTJ-3pstkj-5SqtD-hq23wf-91p8hB-biadpM-8svBXE-9DGqoP-o6ka2p-9aGge7-2wUBxL-5eb6WR-rsyWo3-ukA497-adGq3S-dDM3Fc-7FL5GU-qPrCdv-9zHGtr-LywBb-4Deq5W-94nZNQ-9hy5vX-82TDxP-dSaDo4-cqcE8w-9o1cJx-5jFUSL-R54BE-2zgtdQ-9o4fi9-vAvyuA-a67mjf-F95n3-6GrpCQ-7wUuJm-a3YpD8-6LVgDF-7Yv61v-pbmrgB-67Lf5a-8o595t-fEsZtb-fZpdpK
beach 2	FreeImages.com Content License	http://www.freeimages.com/photo/paradise-1378481
diapers	commons	https://upload.wikimedia.org/wikipedia/commons/d/d8/Disposablediaper.JPG
mold	creative commons attribution	https://www.flickr.com/photos/scrapygraphics/100317097/in/photolist-9S9M8-5Fm6xr-F9DXc-3T6uV3-AdKDC-fquEvJ-7UDbmx-9k1FN4-63qRt-57HAD5-dQDrfo-dQDr7w-56LT55-4mUsH9-73H1yG-oeCncq-4juSeB-m17NGa-57DwRT-bzpyVi-8JUuZG-8DPQ7k-AdKBw-vMToP-9THi8-4RFk-6PHnW2-7teukA-9U1GA-7biwuU-BM4pY-9aDqQ-fm2Lta-6LSDrt-5PQb4u-D1QhQ-gWkQJC-qkYy4-4M53Yy-8JRpfe-9Gg2t9-dgMVEj-e6pXPe-3BgsUp-4Epycp-gWmMmR-adbDjx-8DPQ9z-8JUthf-4D7C3z
rat	creative commons attribution	https://www.flickr.com/photos/alexk100/1091960123/in/photolist-2EuzGF-2jo7rU-wEag9-fNQfvF-bAivYt-5inrHC-2ghCrs-2gi2GQ-2gi2BS-yxUv9-5funF9-8A8TNU-2Euzgp-2Ez2pb-5fq13c-5fq1kg-8pX1yD-xhzR18-bSt1uc-FeLj9-gci3FV-5funoj-8Zd7nZ-wCFYH-wEk5H-7v6AFz-bnoAFd-rxtCME-xqGSF-aDoMD7-6S8Ba-xWakz-5sgmYm-dpUmNQ-5fpZYH-6CWYm9-8XsC36-yDWEAk-yXfwqB-4pLZiY-9vZXJy-yDS3DC-y2ZNW2-nfcnKj-Fe8a2-rZSNaR-yZFpkH-6nFqJU-qAMMPT-oteF7E
talking	creative commons attribution	https://www.flickr.com/photos/polandeze/1206596658/in/photolist-2QC89E-oVcieH-4zfMJ-8WrMyk-aw5Ze7-2VGfUx-8nBf1e-kdtYe-9BBi5g-iAxDtD-k1VCxV-6jKeww-4rhr3M-56QyKv-bkhtF2-ax643X-7FHqDC-5xi8KT-8nZs5P-53M5he-8gX54h-8phdX9-31B7wG-5bhetg-7fXa97-6ASji4-5xi6cg-6Kgst4-Hgvsr-4stfWT-5XsxwH-pfP5wu-4EYA2F-eayK1s-5xhJpB-pB5UTe-ajmari-bzz8mU-jirwzp-9hzA4K-feSVtP-zj6njn-pixuTA-qWMctb-pNfEcD-7jYszV-3nt85E-ci82s-8dZpPc-pqTwWW
snail	FreeImages.com Content License	http://www.freeimages.com/photo/snail-1557298
wedding	creative commons attribution	https://www.flickr.com/photos/whorus3/13926526716/in/photolist-ndD6cA-nfHTeU-atYenM-ajqdXW-cPBrWj-8aqkkf-bxUX49-7VzCR4-e9DjLZ-e9JZYC-fzWasR-9kTKdt-8anM44-9qGuLB-mW9T5i-fjZ8sP-8aqjsE-e9DjyD-9CnMi6-dMF7sY-aB8abK-dMFedS-9CqFTy-8anGCv-8aqyjN-dFnHB3-4wF4ES-hQPac-byYUnJ-dMD4Hj-8aqU49-eFSQR5-ha5My9-aFMzV8-2NBidy-ahZ5Uj-a6rzy9-fkegLj-5wmG1f-9qGtBc-bYMJmy-o3JAx3-ffvffE-PZ714-ymcu8o-nT7AcK-t2awQB-aKzBcZ-29mLZK-y22gMf
injury 3	creative commons	https://www.flickr.com/photos/dougbeckers/6630346217/
listening to music	creative commons attribution	https://www.flickr.com/photos/nkashirin/5325053378/in/photostream/
cake 2	creative commons attribution	https://www.flickr.com/photos/michaelrperry/9501040588/in/photolist-n5YA8-zGjmN-4fc1bT-9LNkHD-9JrLk9-cwAQkG-6PxYR3-5JDvxK-9JoXf4-9JoXYR-5JDvyH-ftzjmC-9eMcZa-9eMawD-9u4XcP-5JHLZy-7RyzyN-9Jp1Ea-9JrPDS-9JoZ1V-5sRq18-9u848y
Prešeren	creative commons	https://www.flickr.com/photos/13706945@N00/8106907641/in/photolist-dmo2cF-6G8eBN-e5obet-95ajQi-95dmpy-95drn3-95dqLE-95ajo6-95dnwm-95dtfE-95duju-95dqjm-95drQJ-95apCv-95aof6-95dnYC-95dph5-95dpLh-95dsMA-95agR8-dPUC7K-e5m6dc-jgJoBZ-jgGFcD-

		e5uQHm-7GaRS1-e34b3d-e34nm1-npPgAU-npuKgP-npvoyK-npuQPU-npvSjz-npM2RK-nryFNZ-npQjs3-npQftG-npMiqF-npPVB3-nnK9SW-npMmXR-nnKhgJ-nryPza-npMFSK-npM6zX-npNaHz-npPczY-npPJo1-npPN2o-npv8z6
ring	FreeImages.com Content License	http://www.freeimages.com/photo/ring-1425671
bird	FreeImages.com Content License	http://www.freeimages.com/photo/bird-1408461
pig	creative commons attribution	https://www.flickr.com/photos/climberaj04/6190198854/in/photolist-ar1oss-zKPYbw-dnNc2C-4WQDQ3-aAqdLi-8Rxt2D-5G1qG-6dkYNX-8988Rb-nCNd6T-95hFAa-znG8XS-53oSE1-nmiTyg-nCzng9-nmivK8-dM9qeJ-nCzmQj-nCzmTf-nCMNVo-nEzt14-nEzsTa-6yq8SS-5667Wb-6jsyeK-5ir8Pi-neVBtd-nEzsUT-7tKSnw-nmivLv-nEzsoH-nEzskg-nmiwhR-nCNcsD-4T3hxn-ah5bJq-6T7dGe-nCNdBH-4bKHo2-2fHACx-DBd3X-baiaTD-ihgVW-7kAEjY-aWTJee-9fgfHg-e9Rs4d-m3cBX-diTjZG-7VmZfs
computer	creative commons attribution	https://www.flickr.com/photos/julioaguiar/2181271770/in/photolist-4jKA6Y-dkJk6r-diXTpY-8kYNbz-5j51tK-eiFSkq-dkJkjg-5LQxjz-8kYPpK-8yxJTR-A3EBS-dkJnnU-8kYCW6-9rHGs9-8m2J4f-6DyDJw-5tAWFQ-5Bwdks-3bQtTv-toZZ2-cPCJu7-68QFUK-aTeCjX-3UW5y-aVQ7Zc-4BdXk9-tHyY8-bV3SAm-xN7hS-59t3AY-dQnPYF-4BRqog-6fYYHi-4CH1wa-8W5Lku-kH4wpB-8msy5a-9xBJ67-2cfeNp-4oPPdh-51K1YL-Nsf4C-pPMX-8eU9fc-7B4Xii-4BRqRX-zbBUg-6pgNUe-9rR5vR-5Wrwym
rocket 1	CC0 Public Domain	https://pixabay.com/en/lift-off-rocket-launch-spacex-693257/
rocket 2	CC0 Public Domain	https://pixabay.com/en/rocket-launch-spacex-lift-off-693269/
ambulance	creative commons	https://www.flickr.com/photos/24874528@N04/8713777408/in/photolist-eh1orw-fKHAm6-5v8Tqs-7Gdio2-8K3g6r-4TRPF4-9YP29k-bpdo8T-8hccVu-9pFarq-8hc5kS-6ikQtg-61AiCr-zxYksS-7MdURB-8b1dAN-ainxJY-9v6wsY-92mPbV-986GAK-f8GVbZ-afXHpZ-3pD66K-eLuC1i-98WzEF-aFfJen-k1Sj-8k3isX-4m2kpo-kUTafG-ptrx5Z-8KjqD5-7yu6fJ-98ZJxo-fCezsY-98hmcm-amL9Dd-9a1xeD-93aLbc-bpD5Ke-qTWRGv-7YfYfF-55UA1h-5cp6vS-odmKXB-8ZQGjq-8gibBy-8zCHNp-67Nk6f-drJWD8
life jacket	creative commons attribution	https://www.flickr.com/photos/ajay_suresh/13707016483/
small fish 2	public domain	http://www.jup5.com/wp-content/uploads/2014/03/Clown_fish.jpg
fishes	creative commons	https://www.flickr.com/photos/mal-b/6981180591/
gloves	creative commons	https://www.flickr.com/photos/mararie/2285451839/
racket	creative commons	https://www.flickr.com/photos/stevendepolo/4637294887/in/photostream/
cup	FreeImages.com Content License	http://www.freeimages.com/photo/red-cups-3-1329258
small fish 3	creative commons attribution	https://www.flickr.com/photos/casamatita/13991603868/in/photolist-njoCns-5dJrsN-edgmPp-5fZsvX-5fZswg-bGuj64-9iDv4u-bsVpv6-5fZswc-6PNbTL-AevWS-jQpsir-5GYL56-jQrezh-8AQypi-yyHWn-9eFbL-nLLu5v-jQrjPL-q6WV4-9eFds-7hftPa-7E8wWF-94JUra-5GYKsP-cbHVJN-7Ecne7-cFUgmS-7WEFoG-bJ6Zza-iSVqUc-5WFABM-eQpwRk-6QVzk-BjTEW-pvrR7-jQpTXT-bB2imv-6PNawY-qt9UV-dFHqE1-ragX6s-6dW8xD-54YBZs-9jHHrM-6kDd5o-5UvK3W-9eJ9BB-rDxmbJ-5Kxnfh
seven	creative commons attribution	Mind & Brain Laboratory
segway	creative commons attribution	https://www.flickr.com/photos/rosenfeldmedia/3265662260/in/photolist-5YznBJ-2qh3FU-4qHrE-5zteoJ-2JtQMq-arSvvX-8Uca7p-8Ucdae-7DqZCn-5zoMov-3ispZ6-81jDVh-a1oQpB-zvSXyo-4qK5W-5zoXkr-3is3ut-zz4UPa-7DqZhF-81jDYo-81gwLV-5qzZGm-a1rDT3-5Sisf-7CeMa6-4qHf2-69CVyJ-4qHDm-8Ucdxg-6Jo7Ux-79HfB-81jGD3-zgFDhc-zgAXff-zx7BvQ-zx7K1j-4tXRH7-zgzcVU-4tTU2n-zKr6g9-seD8J3-cCueJ-cCukG-4uyUvU-71ioP6-4tXU4Y-4tTNm6-4uyRVW-tRwoYb-4uuU1B
axe	FreeImages.com Content License	http://www.freeimages.com/photo/the-axe-2-1464795
six	creative commons attribution	Mind & Brain Laboratory
hungry child	creative commons	https://www.flickr.com/photos/javiercorbo/8333421294/
hungry dog	creative commons attribution	https://www.flickr.com/photos/wonderlane/5331432675/
whale	creative commons	https://www.flickr.com/photos/jdegenhardt/3940510392/
pulley	FreeImages.com Content License	http://www.freeimages.com/photo/pulley-1208871
dessert	creative commons	https://www.flickr.com/photos/manggy/3056627702/
ice cream	FreeImages.com Content License	https://www.flickr.com/photos/thoughtandsight/14764412133/in/photolist-ouFtkT-gitmtA-jzBPGM-cRRweo-6j3Bc-4XKNVe-9jao8G-yZU3JA-88GmEt-aAViDV-6gSTbi-3XCBS-dGa6DG-95sEiy-eTcnaw-7vEm6z-9y5HrR-7wKeng-8DAXTB-nYerAF-a65tc5-5mMowz-5HBSRc-cZESaJ-4z5vv8-9iwXoy-9iwSuG-xYegF2-7NiAVf-dXQ8B4-6UWADW-fynm77-8tkoT8-ae2t7v-8bP2TD-z9hBGq-zrJY7T-7TEuM4-zTjLDb-4tynkp-7ZkByt-kmLuTr-bm2Gpq-6NEx3V-kUfZg6-9Pm9Zp-4SPoDV-7eaPJ4-PmEc3-o2kCMa
plum	creative commons attribution	https://www.flickr.com/photos/134573731@N04/18854475634/in/photolist-uJ77J7-s7Au5w-4sBzhv-4vDkjy-oquhAQ-9H6yAz-9GQfBH-9GQg5H-oKVCJQ-5198K7-oKUVSF-9QcTPH-fGi7xB-4uPyF8-4qf4vK-61edsC-7QNbRm-53daym-9U7GzU-bqqrpx-atc6do-at9osR-9ymVfU-9ymUWS-7QJU1B-dNJY4Y-p1oY77-fGzGpd-a4LXZK-64xfdy-uFd3YJ-9a2wsg-jFYm2D-9ymSWf-a4PNXE-9yiTwB-eaLRGe-9ymYZm-4yew4n-5syLUj-dJyngY-baz1Zr-9Gxsvv-9TXAad-cYWiyQ-5b5mn7-xgEQv2-53exDU-rrGhLG-qCxmBz
elephant	FreeImages.com Content License	http://www.freeimages.com/photo/elephant-1477059
laughter_child	creative commons attribution	https://www.flickr.com/photos/67527337@N00/3077337501/in/photolist-5FWae8-7i6aG7-bkz9ct-7cJLvS-6mimoh-xURF3-4XSn65-bFivG4-7rMGk4-5jR1jB-6pyJMx-nRdpAX-6JACm9-gd8jQd-rDnEGY-6pCy21-6pCmrA-6pCrtu-6ZDBnt-8nwbUg-y9iiMh-6PC8mX-4rD7xq-6yxDjE-neQRE4-82ogKR-ggyWkA-a19fN7-wtB6Ud-6pyzKv-qFni2a-rMqTCD-dStcQ8-7rRCwd-

		5NdtMU-7HK2uN-51Ts9q-9mKViM-8bznBU-8EMyn-6mvo7s-6JAVBY-4v4iwo-4AQVuL-4q4BfN-ewcYth-7FNwkJ-5RHAQd-9mNW97-5jVxXW
laughter	creative commons attribution	https://www.flickr.com/photos/wickenden/3644297054/
laughter_people	creative commons attribution	https://www.flickr.com/photos/eschipul/199259734/
cream	creative commons	https://www.flickr.com/photos/michellecarl/5195393978/
garbage	creative commons attribution	https://www.flickr.com/photos/artonice/4600394956/in/photolist-81we9q-whjfwY-fXgwPM-e8vz6i-5adKLi-aoYiwP-9ujek6-3ag79-ud3PSE-7GnEJg-BJE2P-bB9GxR-7YLVM8-9g4QU1-9Yj1cc-5cKQe9-aaUFP1-p8Yrgz-oRKK8e-kYFsa-81t8N2-5hp7fR-sa5JFg-czmei1-5uhnce-aaRRMa-8puAwJ-6maqL5-sAArzr-uZZ3AW-6F3q5N-ddKdPQ-nZZvJC-2hvY95-fgHJh2-8zvNr8-aahoN8-nqCbxw-5rQ35r-aaUGAL-aaUGtm-9un1ih-9hH45U-5oFdNd-aahoNP-a96qCc-bAFMHv-4ay9zU-aaRSpx-aaRSjM
dump	creative commons attribution	https://www.flickr.com/photos/julienbelli/20356341368/in/photolist-x1Pz5U-8wvirA-atuZSH-286zZH-sTu1S-63eLfC-dUFV1S-8pbwhZ-nhzT8k-j7Usue-p4NF1V-7XoCoe-egEyMY-5kXZB9-51YEnw-6Urzbm-6gfCiL-bpCUYX-3qBVVw-efjiKk-6CZkQQ-qaaW9u-2Z9Tpq-pxnb4q-7Naigo-cQzQJY-uCLmuz-731BML-tJtibg-4Ro2BN-qYdnNr-oMgVfU-4Wz68q-awbDUQ-vmto3A-tFjaUK-o5ZVV8-564LSA-7W7NWG-ihDg7k-989dEq-9865Kx-989cCE-9864Jn-9864cc-7JXRz4-9867Et-9867mc-989e9w-dSxBY8
sun	creative commons attribution	https://www.flickr.com/photos/judepics/15195385056/
four	creative commons attribution	Mind & Brain Laboratory
shooting	creative commons attribution	https://www.flickr.com/photos/familymwr/7249028768/in/photolist-c3zaGC-cpN7hq-8BwMn8-dstuF-BBpmr-9KTpX3-9KTDLf-9KQA6X-9KQApK-c3ziw3-9KQpVz-2Huaeb-HW2cg-c3zaTw-4uh58R-4aUDhM-odKDcx-ejsHP9-4aUDma-bxxDE3-6KWcrX-bZPnwJ-c2tRnb-9KTmYw-ob5puP-nDZ58j-9KTnnd-9KTG7G-9KTkWW-9KQq2Z-9KQSfH-9KQE3T-9KQr6P-4uh5r8-2Lu7v-5BLUEc-4tx1CG-9KQBbi-9KQJgM-oeZBzf-9KQU66-9KTkKb-9KTgaC-9KTokQ-9KQVgV-4mE6Le-9KQJR4-9KQptp-9KQzMR-ca4Kwh
swastika	creative commons attribution	https://www.flickr.com/photos/fw190a8/3480602559/in/photolist-6iyZSX-AoyWzw-5DX5uJ-y6BzQ-9ZpPDX-2jsnFa-7QJGyt-dqFKsL-7fU7RR-3p4ey-afuGh2-6TEPay-4EBgKN-5Jttte-6hi5EF-9845dQ-9JaYis-w9Nh84-fe5ySb-7hbkaf-5Pdgh3-7Ax8xy-6P9THC-gR9Ke-7wxEZm-7hbqHf-tMiYk-ofjeL7-5S3wEy-9NFJas-8ngmL-7oH6Q-vJkjRF-KjyFT-4ovCgk-5XZLQE-9HA1Ua-FJ2va-4RoYaP-xfZ5Ma-4AMRd7-e3xRb3-49N7mf-QdSFF-5seXMH-6f8UCQ-4A6tgg-bhCUb6-6HQAJG-6xAZVN
candle	FreeImages.com Content License	http://www.freeimages.com/photo/candle-1537873
running	creative commons	https://www.flickr.com/photos/chrishunkeler/9055943792/
TV	FreeImages.com Content License	http://www.freeimages.com/photo/spooky-tv-ghost-static-1535787
keyboard	creative commons attribution	https://www.flickr.com/photos/126089327@N04/14714880858/in/photolist-oqiBqo-ayDqJ7-ayDqAb-ayAKna-574Jv7-ksh2Hp-knMJ6Z-ah15GZ-7x7o1q-47dnYK-6Qpo2B-7CKDd7-8JHvxe-doR96U-82wbZN-c28AgE-4KPnVE-9eCVPc-8JHvDT-epQK5J-dAFtD6-47doa6-52enYL-aZaWJg-9jjwCn-6DwLPD-e76N8i-bzLPgd-35JJ8t-HosRF-8LRcdX-nQUnVq-8TUDu8-aBEV1u-d4Mm2h-hYTz2-83ojv6-hwoHar-4QvoK8-rzpwtk-mcgU11-nPdcjj-8m2zS5-ojMwWm-7vqNu2-6PmvEp-sD8V2B-jT5U21-6NVoGc-77xzJC
cake	creative commons attribution	https://www.flickr.com/photos/jamieanne/4657231941/in/photolist-86xwNr-6ggGWa-5HvzVF-3Dt6iE-9UCJto-4gwxAv-boyt1K-7wrMUH-9jrqCx-9hsN6K-z9g5JN-9XWQ1K-nKcxdx-3aYkCx-bn12AH-8BDZT3-nhSzUR-rampuB-9ouGtf-6yGVQ4-byC5Qh-9HUVxq-eeutnb-4tADBa-eQKgc4-vzhQLK-bHcJ7p-vexNZq-7dXk42-d9P6UL-e9Szv3-7XHj1n-nLuVzq-ekCLU3-vq8DBi-9DE8Wd-6WHkr9-dSHHrg-8r6dhB-r5mgtS-agqBfu-ddsRMY-7f1PuU-5Ddan2-eBKZAR-e66J8M-9DBgmi-7f4ipA-9UzVHP-r5su98
tractor	creative commons attribution	https://www.flickr.com/photos/agrale/10857727905/in/photolist-hxsGeg-e64jY2-euY48W-oDijuC-e6f4ey-etUwdV-e7jvru-Sxip8-e4JevE-e6cXQu-bjHc82-buA2cC-e69mdx-cbPzKA-buzQeo-hxurNr-drTXU8-gbBxYK-e6mfg9-e69ASt-6TFYwN-e5ZU2T-buzHou-e67jnM-e67miF-buzTAu-bHuAnt-2992VR-eukR8a-e67khv-e66f2w-8t7ThL-e63rzX-e66stH-e66Y7T-e5C4Qs-4UeiC4-e6tA9m-e6mb91-aePJgi-9rSVh1-oDHubd-foxYRp-fkNnEG-cAWRLN-fkNo9A-zbJrmS-4WiFmU-7VhLSJ-euXSMC
three	creative commons attribution	Mind & Brain Laboratory
Triglav mountain	FreeImages.com Content License	https://www.flickr.com/photos/anschieber/21570479895/in/photolist-2UtCez-2Uy1jd-2Uy11N-2UxZGu-2UtBj6-2UxZdQ-2UxZ63-2UtAHR-2UtAnR-2UtA3c-2UxXUL-2UxXAo-2Utz6v-2UtyLp-pCDSnk-8igbKw-dMtXHs-8igbKb-bLbKCk-yS7maM-2Utyzt-2UxWLy-2UtygF-2Uty68-2UtxSz-2UxW3s-2Utxxe-2UxVFj-2UxVtj-2UtwVr-79jAUz-pmrmTs-pCU21f-76mcti-79osTS-79osQm-79jBn6-d1o5FC-8XcUGJ-e6J5x7-8wmVv9-8unWi3-bC5DzZ-8J5S2a-bC5BFt-76q7mL-76mbtH-pws4KM-fAsKKb-g9VQ3e
ear ring	FreeImages.com Content License	https://www.flickr.com/photos/paparutzi/365420835/
clock	FreeImages.com Content License	http://www.freeimages.com/photo/clock-1426359
cut	creative commons attribution	https://www.flickr.com/photos/hile/16805248356/in/photolist-rB2hRj-64JYwU-4rD7xq-9VEPcF-9KHMS7-fzZsEo-kj9dug-hZ7qHj-hLB1BA-e1H1v8-jLYUoG-ihdYUi-jQA1mg-93rrS2-nUNEbK-4cAxWH-3HnGN-jkWg3d-72L6mA-7gABNm-qqaFBp-8YyEzZ-aiPfL7-4K1SxK-dh42Zn-9ZSZax-okkMto-br4bmp-6978aB-6RcC3w-fANxfG-rqZRKz-6QnrSC-rnXdsb-hVt1X-ejpvFR-fhp8vt-7KSzas-e1BLzd-7zjGNZ-eV1YpZ-rB9xmt-chFSbU-tnnM82-fBD9Hh-hu1MSi-4XJsbd-oe4eka-bsKb2H-chFVCu
fork	FreeImages.com Content License	http://www.freeimages.com/photo/fork-1576821
violin	FreeImages.com Content License	http://www.freeimages.com/photo/violin-2-1420085

vehicle	creative commons attribution	https://www.flickr.com/photos/toyota-europe/9198924493/in/photolist-f1STLr-nqe5x9-nqe5Ey-nJuVke-nGHvBv-e6phNG-e6Fvyi-fxjaCh-e79r9q-e6nzYt-e69t1Z-e79CEu-a4dD8N-e6emhy-e6sHdy-e6iEVi-e6iFdV-e6waoP-9aRGV2-e6ptxN-e6J8iz-e6JhZF-e6tkXS-e61yYb-e6Jnnp-e6ps59-e6u76s-k7Wm9q-e6sv4e-8AGHMk-e6f4QN-e6MaLm-e6FbDZ-gigWSM-e6n592-iVSRaK-7Z7T7c-e6evmu-bLyUQB-7Hht7K-adiPjW-e6tkAG-e6nx2F-e6iHta-e6iHRz-8kSais-i1yvQf-e5NDZK-nqdQsJ-f288XW
pattern 1	creative commons attribution	https://www.flickr.com/photos/haabet/4390670438/in/photolist-7FZkms-7FZbSQ-d53saC-5zCab4-bZZwKC-tmryfD-5zGrwb-9jjvZe-7Qqpge-5XcdYR-fxf3ur-5LqxMi-7FV5qk-gdc5W4-5SGPqT-a3v4ry-7BmrQU-cB9uvW-ryhkuW-fxfSbr-d53rZN-fpbhov-7FV8xM-wiKad-5zC9Tc-8tQHnE-6cEvbt-dyAdH8-5SGPy2-cB8Ppj-62mvDL-9vbfgi-5SW8p7-auBKhf-pcsF76-5SGPwv-7pZeAc-6ZTFf4-5sARfd-7Edqxh-5K6EsY-8tdXu7-cRwoXW-7pZeQg-dzZHRM-8F9Htc-9BFqd1-65QYDk-gRVxkm-4nLxfj
pattern 2	creative commons attribution	https://www.flickr.com/photos/haabet/4390641924/in/photolist-7FZbSQ-d53saC-5zCab4-bZZwKC-tmryfD-5zGrwb-9jjvZe-7Qqpge-5XcdYR-fxf3ur-5LqxMi-7FV5qk-gdc5W4-5SGPqT-a3v4ry-7BmrQU-cB9uvW-ryhkuW-fxfSbr-d53rZN-fpbhov-7FV8xM-wiKad-5zC9Tc-8tQHnE-6cEvbt-dyAdH8-5SGPy2-cB8Ppj-62mvDL-9vbfgi-5SW8p7-auBKhf-pcsF76-5SGPwv-7pZeAc-6ZTFf4-5sARfd-7Edqxh-5K6EsY-8tdXu7-cRwoXW-7pZeQg-dzZHRM-8F9Htc-9BFqd1-65QYDk-gRVxkm-4nLxfj-9yjsAu
saw	FreeImages.com Content License	http://www.freeimages.com/photo/saw-1-1416686
rabbit	FreeImages.com Content License	http://www.freeimages.com/photo/little-red-bunny-1372124
sad lady	creative commons attribution	https://www.flickr.com/photos/visualjourney/2438284307/
bulb	FreeImages.com Content License	http://www.freeimages.com/photo/light-bulb-1-1427502
nail	creative commons attribution	https://www.flickr.com/photos/86530412@N02/7984329008/in/photolist-daxLQY-snh3du-4ChL9d-zRJDgm-9Txzxs-9TuJDe-9TxziA-o1oiGf-9TxAaw-9TxzVN-w7cv5r-6UAws2-ajP2d8-7AxVLL-agzTYN-p536VM-voFycM-9TuJRT-gsWHBE-9TxAfd-fQ8gRr-wB8dJf-aMSL2g-9TuJMt-9TxA2W-9TuHST-y7LyRd-58pjLj-aoRAWr-cphGFE-iDkrf6-kLDwrW-9TuHvT-aMSya8-qy33U6-dnVMoS-7Tfkfy-aET4q9-74MDX2-b7RZJM-aoRsPH-hN1f1-vZZLMH-9TuJ78-ovYWf7-i5SnmU-i5FtTd-3LLwuN-ars2hw-6M47pH
woman 1	creative commons attribution	https://www.flickr.com/photos/jumfer/8211520949/in/photolist-dvCc6p-qiZsir-mHuoPF-qffrwa-rbUUj6-qVauJd-r9FnUw-rbEJaB-r9Bsay-rcGmHP-98P1ps-7st9gB-qeeNXL-rTRPfq-rcCUr6-qVhdKA-74rH1b-rcSfAn-mHngz4-9TNaej-qfLvzG-mBnbv1-mFwGXm-mFv7tx-rcRb7p-rbNTgs-vEQ8Du-qeXPLq-apN2yQ-raukoq-rdT9Eg-rTRP6C-rd78JP-mDbsmu-qUvXCv-qVcptj-rdWpDD-4VEyVL-bDDqfj-qBa7Lj-mFuPHv-d2txyq-ohKvkt-ofW38m-odWTD1-nYuge7-ofGcWr-nYuqh5-nYvtPT-ofVZ21
woman 2	creative commons attribution	https://www.flickr.com/photos/125303894@N06/14365668676/in/photolist-nTrNNG-rpHtNg-8mmvmc-qVgcmB-62yr5L-v1aXrn-fejgfj-7pM4ve-sihjjf-r8d663-fjG63h-mXizBZ-eaK7ff-tNw2nU-obJGjs-fcVe1k-9GkEaP-hVqbUz-rWuTcu-mHmdvD-bBuWMg-nTrPpM-sbp3Cn-6Dvnav-sbM4bN-x2zYv-9fH8E1-oXsUeb-a2N9gj-apFzyg-rpGjRw-sbp3mk-sbp3vi-ofVXEo-ofNjhU-nYubsU-ofNhid-nYutUr-sbM443-sdVqB1-rh4RLA-hmVFFc-dHQXMj-Kx2bu-6Dvn5t-bZbbbQ-r6Vx9X-bgYERT-6DviCB-8oFgyT
woman 3	creative commons attribution	https://www.flickr.com/photos/71515883@N05/7022089349/in/photolist-bGw3w2-bx3qYh-ihRuEZ-rbBdmK-48GX6p-8v9yYn-ihRcWQ-bm2XSD-4xwdGq-iYmm1J-bg24LT-bm2XiF-a3Bn2S-93Nop2-26vop-8WYRt7-gzfqd-nWKr1J-nWKBUf-gskEw1-igEiE2-ifEvkJ-5j893c-a6tS79-igHjrX-ejPZgd-odySkB-odwR1r-igJoio-igGiTd-igK5Q5-23CVpg-XrNuw-ifEv5U-igJGtP-9ZboVi-gWYsRA-knRMB9-igJH22-ifEaTg-gsYUwD-2pX6vX-8HCGHt-cF9sYQ-2uHdXQ-ihR5Y3-igJ2De-ifEAUC-aR8k6D-bxjc6m
young woman	creative commons attribution	https://www.flickr.com/photos/enthuan/8597189825/
old woman	creative commons attribution	https://www.flickr.com/photos/robwallace/2193965102/
strange animal 1	creative commons attribution	https://www.flickr.com/photos/53357045@N02/4973030931/in/photolist-8zs5QB-yLWVw5-dTGp3L-9UzVze-9UzUNt-dTGp5j-9uUjTh-9maPRX-4yyTJg-8eAzH7-4fZLpP-eGSNA-nup2Zc-55eMSj-7dhqcP-qqzQDT-5hLzcU-53f3XX-dugF7-nYiFGJ-ibvsUU-9maSRn-9mdSXS-967yMp-dgPiha-9maNMV-9mdRFQ-a2RJeo-49G4ZE-7dm2Qq-55fcfm-8Phen2-4sfpxa-4W8brM-jq8yfe-6KXhiC-55eYaG-711TDL-DNNTY-dnqiy2-andkVn-iXBadD-fnh2Fe-9maNJ8-7dmmGu-8xxbWk-7YYvmV-ofrbnY-7dhdwe-awToiX
strange anima 2	creative commons	https://www.flickr.com/photos/bestrated1/3024509038/
gold fish	CC0 Public Domain	https://pixabay.com/en/gold-fish-nature-water-animal-1022229/
gold	creative commons attribution	https://www.flickr.com/photos/birminghammag/7982539789/in/photolist-daoAYn-qTaRQw-CWz6DY-vHgSuT-4yZkS1-bajeoP-eCrMBt-4s8qKP-cmjE3N-4z1jYe-okB68Y-cmjDFh-bRZ3LR-fm5EF2-fmjPeY-fm5ExM-fkJzSJ-8Ynygy-8PZXjK-fopFjr-5YMFvy-fku1XK-pE2vF-B1Fx2-rTz7Tp-o3dmE3-ojqhL4-hWfp7C-o3dnkm-o3eqXZ-a5dgYy-ojqaPi-o3eqkB-o3d5hf-o3erCB-bvGsQ8-ojEKa9-rfXQsG-o3dj6r-vE6dxu-omsSaz-i89MYy-o3dqK8-ohF6HE-o3douX-bD5hYo-omsYFa-aEDmY5-o3epHp-ojvA1h
spoon	creative commons attribution	https://www.flickr.com/photos/zyada/4690069164/in/photolist-89rQas-89rQHf-DSBGQ-eLGBiL-ehTCpT-gjWBmQ-gjWX6S-8FnE7f-6cj1ko-a4u17i-gjWBcS-t9Rvm-gjXhMc-5QWHVA-5QSqJM-oh8xE2-7a58AG-cEQHPd-9CP86X-5EErvu-bxHpSv-2cV5Gj-bx4EaQ-nZDcji-75zdwK-9TFSq5-dhKsR2-4RZ6MN-7XpKyz-BHBeH-7zL8EE-dCGTti-5QWHV7-75D5Vh-8Cw9ZD-oiTDQc-5n8KCX-nDNEvU-7QgBMi-edaTEr-e1A3R2-nspvGD-85S1Fh-diyGJV-a5wyon-fpfhNk-jD3uky-6h6Dv-9wRnud-rcCeB7
broken leg	creative commons	https://www.flickr.com/photos/danielpaquet/594399093/
dentist	creative commons attribution	https://www.flickr.com/photos/purplemattfish/4012842364/
speaker	FreeImages.com Content License	http://www.freeimages.com/photo/computer-speaker-1499716

bell	FreeImages.com Content License	http://www.freeimages.com/photo/bell-1169784

Table S24
List of C3T auditory stimuli and licenses

Sound	License	Source
meeow 6	creative commons attribution http://www.freesfx.co.uk/info/eula/	http://www.freesfx.co.uk/soundeffects/cats/
meeow 1	creative commons attribution	http://www.freesfx.co.uk/sfx/meow
meeow 2	creative commons attribution	http://www.freesfx.co.uk/sfx/meow
meeow 3	creative commons attribution	http://www.freesfx.co.uk/sfx/meow
meeow 4	creative commons attribution	http://www.freesfx.co.uk/sfx/meow
baby talk 3	creative commons attribution noncommercial http://creativecommons.org/licenses/by-nc/3.0/	https://www.freesound.org/people/Ephemeral_Rift/sounds/77455/
baby talk 1	creative commons attribution	https://www.freesound.org/people/keplar/sounds/139048/
laughter baby 3	creative commons attribution http://creativecommons.org/licenses/by/3.0/	https://www.freesound.org/people/reinsamba/sounds/47370/
laughter baby 1	creative commons attribution	https://www.freesound.org/people/Stevious42/sounds/259625/
laughter baby 2	creative commons attribution	https://www.freesound.org/people/OBXJohn/sounds/242932/
ship	creative commons attribution	https://www.freesound.org/people/milo/sounds/23722/
siren 6	creative commons http://creativecommons.org/publicdomain/zero/1.0/	https://www.freesound.org/people/guitarguy1985/sounds/70938/
siren 1	creative commons	https://www.freesound.org/people/FatLane/sounds/111671/
siren 2	creative commons attribution	https://www.freesound.org/people/CGEffex/sounds/121902/
siren 3	creative commons	https://www.freesound.org/people/conleec/sounds/159754/
siren 4	creative commons attribution noncommercial	https://www.freesound.org/people/ondrosik/sounds/171094/
siren 5	creative commons	https://www.freesound.org/people/guitarguy1985/sounds/58015/
bell6	creative commons	https://www.freesound.org/people/tec%20studios/sounds/99625/
bell 1	creative commons attribution	https://www.freesound.org/people/Zabuhailo/sounds/178646/
bell 2	creative commons attribution	https://www.freesound.org/people/joedeshon/sounds/78506/
bell 3	creative commons	https://www.freesound.org/people/UncleSigmund/sounds/36327/
bell 4	creative commons attribution noncommercial	https://www.freesound.org/people/Robinhood76/sounds/320724/
bell 5	creative commons	https://www.freesound.org/people/ottophokus/sounds/78403/
caughing 1	creative commons attribution	http://www.freesound.org/people/joedeshon/sounds/266019/
caughing 2	creative commons attribution	http://www.freesound.org/people/OwlStorm/sounds/151217/
caughing 3	creative commons	http://www.freesound.org/people/Eelke/sounds/184871/
caughing 4	creative commons	http://www.freesound.org/people/qubodup/sounds/169726/
hammer 5	creative commons attribution	http://www.freesound.org/people/WIM/sounds/17908/
hammer 1	creative commons	http://www.freesound.org/people/olliehahn12/sounds/262000/
hammer 2	creative commons	http://www.freesound.org/people/amsempl/sounds/151949/
hammer 3	creative commons attribution noncommercial	http://www.freesound.org/people/Robinhood76/sounds/106960/
hammer 4	creative commons attribution	http://www.freesound.org/people/kwahmah_02/sounds/250257/
barking 1	creative commons	http://www.freesound.org/people/LittleBigSounds/sounds/163459/
barking 2	creative commons attribution	http://www.freesound.org/people/Anton/sounds/157322/
barking 4	creative commons	http://www.freesound.org/people/felix.blume/sounds/199261/
chainsaw 2	creative commons	http://www.freesound.org/people/esperri/sounds/118972/
chainsaw 1	creative commons	http://www.freesound.org/people/doobit/sounds/65997/
saw 2	creative commons attribution	http://www.freesound.org/people/JoelAudio/sounds/135859/
saw 1	creative commons attribution	http://www.freesound.org/people/Jagadamba/sounds/258055/
drill	creative commons attribution	http://www.freesfx.co.uk/sfx/drill
alarm clock 4	creative commons attribution	http://www.freesound.org/people/bone666138/sounds/198841/
alarm clock 1	creative commons attribution	http://www.freesound.org/people/kwahmah_02/sounds/250629/
alarm clock 2	creative commons	http://www.freesound.org/people/eriklindmanmata/sounds/266668/
alarm clock 3	creative commons attribution noncommercial	http://www.freesound.org/people/zanox/sounds/233645/
horse 2	creative commons	http://www.freesound.org/people/foxen10/sounds/149024/
horse 1	creative commons attribution	http://www.freesound.org/people/dobroide/sounds/18229/
gun 3	creative commons attribution	http://www.freesound.org/people/knufds/sounds/78776/
gun 2	creative commons attribution	http://www.freesound.org/people/joyce137298/sounds/187200/
ambulance	creative commons attribution noncommercial	https://www.freesound.org/people/Robinhood76/sounds/256469/
excitement 3	creative commons	http://www.freesound.org/people/Pep_Molina/sounds/220691/
excitement 1	creative commons	http://www.freesound.org/people/RatSalsa/sounds/170208/
excitement 2	creative commons attribution	http://www.freesound.org/people/unfa/sounds/270301/
brakes 2	creative commons attribution	http://www.freesound.org/people/dobroide/sounds/86933/
brakes 1	creative commons	http://www.freesound.org/people/virelilisoi/sounds/176068/
applause 3	creative commons	http://www.freesound.org/people/looijenga/sounds/277022/
applause 1	creative commons attribution noncommercial	http://www.freesound.org/people/ascap/sounds/242581/
applause 2	creative commons attribution	http://www.freesound.org/people/Halleck/sounds/18665/
car's engine	creative commons	http://www.freesound.org/people/RutgerMuller/sounds/50898/
brakes_car 2	creative commons	http://www.freesound.org/people/RutgerMuller/sounds/104026/
brakes_car 1	creative commons	http://www.freesound.org/people/monnie101/sounds/58150/
automatic saw 2	creative commons attribution	http://www.freesound.org/people/suoitnop/sounds/66264/
automatic saw 1	creative commons attribution	http://www.freesound.org/people/kwahmah_02/sounds/250060/
guitar 3	creative commons attribution	http://www.freesound.org/people/afrodrumming/sounds/187696/
guitar 1	creative commons	http://www.freesound.org/people/spitefuloctopus/sounds/315705/
guitar 2	creative commons	http://www.freesound.org/people/SeryLis/sounds/181425/
piano 2	creative commons	http://www.freesound.org/people/Bradovic/sounds/164718/

piano 1	creative commons attribution	http://www.freesound.org/people/Aiwha/sounds/196103/
piano 3	creative commons attribution	http://www.freesound.org/people/casualsamples/sounds/64829/
scream_child 2	creative commons attribution noncommercial	http://www.freesound.org/people/Robinhood76/sounds/134762/
scream_child 1	creative commons attribution noncommercial	http://www.freesound.org/people/Robinhood76/sounds/168777/
mouse 2	creative commons	https://www.freesound.org/people/AntumDeluge/sounds/188043/
mouse 1	creative commons attribution	http://www.freesfx.co.uk/soundeffects/rodents/
motorbike 2	creative commons attribution	http://www.freesfx.co.uk/soundeffects/motorcycles/?p=1
motorbike 1	creative commons attribution	https://www.freesound.org/people/mikaelfernstrom/sounds/68710/
rooster 2	creative commons attribution	http://www.freesfx.co.uk/soundeffects/birds/?p=5
rooster 1	creative commons attribution	http://www.freesfx.co.uk/sfx/rooster
bird 2	creative commons attribution	http://www.freesfx.co.uk/sfx/birds?p=2
bird 1	creative commons attribution	http://www.freesfx.co.uk/sfx/birds?p=2
bird 3	creative commons attribution	http://www.freesfx.co.uk/sfx/bird
bird singing	creative commons attribution	http://www.freesfx.co.uk/sfx/birds?p=3
burping 2	creative commons attribution	http://www.freesfx.co.uk/sfx/belch
burping 1	creative commons attribution	http://www.freesfx.co.uk/sfx/belch
laughter 1	creative commons attribution	http://www.freesfx.co.uk/sfx/laugh?p=1
laughter man 2	creative commons attribution	http://www.freesfx.co.uk/sfx/laugh?p=2
laughter man 1	creative commons attribution	http://www.freesfx.co.uk/sfx/laugh?p=2
train 3	creative commons attribution	http://www.freesfx.co.uk/sfx/train?p=4
train 1	creative commons attribution	http://www.freesfx.co.uk/sfx/train?p=4
train 2	creative commons attribution	http://www.freesfx.co.uk/sfx/train?p=4
car honking 2	creative commons attribution	http://www.freesfx.co.uk/sfx/horn
car honking 1	creative commons attribution	http://www.freesfx.co.uk/sfx/horn
drums 2	creative commons attribution	http://www.freesfx.co.uk/sfx/drum?p=2
drums 1	creative commons attribution	http://www.freesfx.co.uk/sfx/drum?p=3
cymbal	creative commons	https://www.freesound.org/people/minorr/sounds/104214/
crick	creative commons attribution	http://www.freesfx.co.uk/sfx/cricket
eating chocolate	creative commons attribution	https://www.freesound.org/people/AudioRichter/sounds/169342/
sighing 2	creative commons	https://www.freesound.org/people/SavvahSjuhengof/sounds/325545/
sighing 1	creative commons	https://www.freesound.org/people/benoitburke/sounds/244368/
crying man	creative commons	https://www.freesound.org/people/qubodup/sounds/200428/
crying baby 2	creative commons attribution	http://www.freesfx.co.uk/sfx/crying
crying baby 1	creative commons attribution	http://www.freesfx.co.uk/sfx/crying?p=1
crying woman	creative commons	https://www.freesound.org/people/AderuMoro/sounds/272093/
goat	creative commons	https://www.freesound.org/people/Erokia/sounds/188182/
cow	creative commons attribution	http://www.freesfx.co.uk/soundeffects/cows/
scream man	creative commons attribution	http://www.freesfx.co.uk/sfx/scream?p=2
sheep	creative commons attribution	http://www.freesfx.co.uk/sfx/sheep
beep sound 2	creative commons attribution	http://www.freesfx.co.uk/sfx/beep
beep sound 1	creative commons attribution	http://www.freesfx.co.uk/sfx/beep
applause 4	creative commons attribution	http://www.freesfx.co.uk/sfx/clapping
kiss	creative commons attribution	http://www.freesfx.co.uk/sfx/kiss
fart 2	creative commons attribution	
fart1	creative commons attribution	
laughter people	creative commons attribution noncommercial	https://www.freesound.org/people/andriala/sounds/16200/
laughter woman 2	creative commons attribution	http://www.freesfx.co.uk/sfx/laugh?p=2
laughter woman 1	creative commons attribution	http://www.freesfx.co.uk/sfx/laugh?p=2
shooting 2	creative commons attribution	http://www.freesfx.co.uk/sfx/shooting
shooting 1	creative commons attribution	http://www.freesfx.co.uk/sfx/shooting
sound 3	creative commons	https://www.freesound.org/people/pinkyfinger/sounds/68448/
sound 1	creative commons	https://www.freesound.org/people/anysounds/sounds/35816/
sound 2	creative commons attribution	https://www.freesound.org/people/digifishmusic/sounds/94812/
tractor	creative commons attribution	https://www.freesound.org/people/viertelnachvier/sounds/157822/
wind	creative commons attribution	https://www.freesound.org/people/Benboncan/sounds/84111/
violin 2	creative commons attribution	http://www.freesfx.co.uk/sfx/violin
violin 1	creative commons attribution	http://www.freesfx.co.uk/sfx/violin
alarm	creative commons attribution	http://www.freesfx.co.uk/sfx/danger
car	creative commons attribution	https://www.freesound.org/people/RHumphries/sounds/1930/
white sound	creative commons	https://www.freesound.org/people/theundecided/sounds/165058/
drilling mashine	creative commons attribution	http://www.freesfx.co.uk/sfx/drill?p=2
vomit	creative commons attribution	http://www.freesfx.co.uk/sfx/vomit
tool	creative commons	https://www.freesound.org/people/thearxx08/sounds/273722/
dolphin	standard youtube licence	https://www.youtube.com/watch?v=cN0H0g4pZaY
rain	creative commons attribution	http://www.freesfx.co.uk/download/?type=mp3&id=3548
flute	creative commons attribution	https://www.freesound.org/people/juskiddink/sounds/65510/
sea gull	creative commons	https://www.freesound.org/people/Snapper4298/sounds/166707/
eating chips	creative commons attribution	http://www.freesfx.co.uk/sfx/eating
crying baby 3	creative commons attribution	http://www.freesfx.co.uk/sfx/baby?p=4
klarinet	creative commons attribution	http://www.freesfx.co.uk/sfx/clarinet
clarinet	creative commons	https://www.freesound.org/people/Rudmer_Rotteveel/sounds/316920/
hiccups	creative commons attribution	http://www.freesfx.co.uk/sfx/hiccup
march music	creative commons attribution noncommercial	http://www.freesound.org/people/zagi2/sounds/182311/
scream woman	creative commons attribution	http://www.freesfx.co.uk/sfx/scream?p=3
scream woman 2	creative commons attribution	http://www.freesfx.co.uk/sfx/scream?p=2

xylophone	creative commons attribution	http://www.freesfx.co.uk/sfx/xylophone
barking 3	creative commons attribution	http://www.freesfx.co.uk/sfx/bark
fox	creative commons attribution	http://www.freesfx.co.uk/sfx/fox
pig	creative commons attribution	http://www.freesfx.co.uk/download/?type=mp3&id=10236
wolf	creative commons	http://www.orangefreesounds.com/wolf-howl-sound/
eating ice cream	creative commons	https://www.freesound.org/people/yummie/sounds/176798/
sea	creative commons attribution	http://www.freesfx.co.uk/sfx/sea?p=1
fly	creative commons attribution	http://www.freesfx.co.uk/sfx/fly
storm	creative commons attribution	http://www.freesfx.co.uk/sfx/storm
laughter children	creative commons attribution	http://www.freesfx.co.uk/sfx/baby?p=4
ouu_yeah_sound	creative commons attribution	https://www.freesound.org/people/snaginneb/sounds/120591/
dog	creative commons attribution	http://www.freesfx.co.uk/sfx/dog
dog 2	creative commons attribution	https://www.freesound.org/people/juskiddink/sounds/121565/
song 1	creative commons attribution	http://www.freesfx.co.uk/sfx/song?p=3
song 2	creative commons attribution	http://www.freesfx.co.uk/sfx/song?p=3
song 3	creative commons attribution	http://www.freesfx.co.uk/sfx/song?p=3
song 4	creative commons attribution	http://www.freesfx.co.uk/sfx/song?p=3
song 5	creative commons attribution	http://www.freesfx.co.uk/sfx/song?p=5
song 6	creative commons attribution	Mind & Brain Laboratory
song 7	creative commons attribution	Mind & Brain Laboratory
song 8	creative commons attribution	Mind & Brain Laboratory
gun1	creative commons attribution	http://www.freesfx.co.uk/sfx/gun
rat	creative commons attribution	http://www.freesfx.co.uk/sfx/rat
breaking window	creative commons attribution	http://www.freesfx.co.uk/sfx/window?p=4
saxophone	creative commons attribution	https://www.freesound.org/people/juskiddink/sounds/77685/
synthetic sound	creative commons attribution	http://www.freesfx.co.uk/sfx/scifi?p=2
synthetic sound 2	creative commons attribution	http://www.freesfx.co.uk/sfx/scifi?p=2
synthetic sound 3	creative commons attribution	http://www.freesfx.co.uk/sfx/scifi?p=2
synthetic sound 4	creative commons attribution	http://www.freesfx.co.uk/sfx/scifi?p=2
synthetic sound 5	creative commons attribution	http://www.freesfx.co.uk/sfx/scifi?p=2
synthetic sound 6	creative commons attribution	http://www.freesfx.co.uk/sfx/scifi?p=2
synthetic sound 7	creative commons attribution	http://www.freesfx.co.uk/sfx/scifi?p=2
synthetic sound 8	creative commons attribution	http://www.freesfx.co.uk/sfx/scifi?p=2
crunching	creative commons attribution	http://www.freesfx.co.uk/sfx/creak?p=2
laughter baby 4	creative commons attribution	http://www.freesfx.co.uk/sfx/laugh?p=3
laughter people2	creative commons attribution	http://www.freesfx.co.uk/sfx/laughing
deer	creative commons attribution noncommercial	https://www.freesound.org/people/MikelRNieto/sounds/201174/
sound 4	creative commons	https://www.freesound.org/people/uEffects/sounds/208055/
tiger	creative commons attribution	http://www.freesfx.co.uk/sfx/tiger
typing	creative commons attribution	http://www.freesfx.co.uk/sfx/typing?p=2
tornado	creative commons attribution	https://www.freesound.org/people/CGEffex/sounds/93101/
truck	creative commons attribution	http://www.freesfx.co.uk/sfx/truck?p=3
trumpet	creative commons attribution	https://www.freesound.org/people/Harbour11/sounds/194624/
trumpet 2	creative commons attribution	zhttp://www.freesfx.co.uk/sfx/horn?p=1
ukulele	creative commons attribution noncommercial	https://www.freesound.org/people/turkitron/sounds/110529/
watch	creative commons attribution	http://www.freesfx.co.uk/sfx/clock?p=1
water	creative commons attribution	http://www.freesfx.co.uk/sfx/water?p=2
happy birthday	creative commons attribution	https://www.freesound.org/people/Percy%20Duke/sounds/23270/
sighing 3	creative commons	https://www.freesound.org/people/Reitanna/sounds/242897/
scream	creative commons attribution noncommercial	https://www.freesound.org/people/Jagadamba/sounds/254337/
party	creative commons attribution noncommercial	https://www.freesound.org/people/Robinhood76/sounds/76453/
